# Supplementary material for: Women’s Involvement in Steady Exercise (WISE): Study Protocol for a Randomized Controlled Trial
Source: Healthcare (Basel). 2023 Apr 29;11(9):1279. doi: 10.3390/healthcare11091279 (PMC10177760; doi:10.3390/healthcare11091279)
Supplement: Supplementary file 1 [file healthcare-11-01279-s001.zip › Table S1_WISE Exercise Protocol.pdf]

# WISE EXERCISE PROTOCOL

Women's Involvement In Steady Exercise

# Index

Index ........ 1
HIIT training protocol ........ 4
Month 1 ........ 5
Common Part ........ 5
Dynamic Warm-up ........ 5
Stretching ........ 9
Main part ........ 11
Week 1 - Session 1 ........ 11
Week 1 - Session 2 ........ 13
Week 2 - Session 1 ........ 14
Week 2 - Session 2 ........ 15
Week 3 - Session 1 ........ 16
Week 3 - Session 2 ........ 17
Week 4 - Session 1 ........ 19
Week 4 - Session 2 ........ 20
Month 2 ........ 21
Common Part ........ 21
Dynamic Warm-up ........ 21
Stretching ........ 24
Main part ........ 25
Week 1 - Session 1 ........ 25
Week 1 - Session 2 ........ 26
Week 2 - Session 1 ........ 27
Week 2 - Session 2 ........ 28
Week 3 - Session 1 ........ 29
Week 3 - Session 2 ........ 31
Week 4 - Session 1 ........ 33
Week 4 - Session 2 ........ 34
Month 3 ........ 36
Common Part ........ 36

Dynamic Warm-up ... 36
Stretchin ... 38
Main part ... 40
Week 1 - Session 1 ... 40
Week 1 - Session 2 ... 41
Week 2 - Session 1 ... 43
Week 2 - Session 2 ... 45
Week 3 - Session 1 ... 46
Week 3 - Session 2 ... 47
Week 4 - Session 1 ... 48
Week 4 - Session 2 ... 49
Month 4 ... 50
Common Part ... 50
Dynamic Warm-up ... 50
Stretching ... 52
Main part ... 53
Week 1 - Session 1 ... 53
Week 1 - Session 2 ... 55
Week 2 - Session 1 ... 56
Week 2 - Session 2 ... 58
Week 3 - Session 1 ... 60
Week 3 - Session 2 ... 62
Week 4 - Session 1 ... 63
Week 4 - Session 2 ... 64
Month 5 ... 66
Common Part ... 66
Dynamic Warm-up ... 66
Stretching ... 68
Main part ... 70
Week 1 - Session 1 ... 70
Week 1 - Session 2 ... 71
Week 2 - Session 1 ... 72
Week 2 - Session 2 ... 73
Week 3 - Session 1 ... 74

Week 3 - Session 2 ........ 75
Week 4 - Session 1 ........ 78
Week 4 - Session 2 ........ 79
Month 6 ........ 82
Common Part ........ 82
Dynamic Warm-u ........ 82
Stretching ........ 84
Main part ........ 85
Week 1 - Session 1 ........ 85
Week 1 - Session 2 ........ 88
Week 2 - Session 1 ........ 90
Week 2 - Session 2 ........ 93
Week 3 - Session 1 ........ 96
Week 3 - Session 2 ........ 98
Week 4 - Session 1 ........ 101
Week 4 - Session 2 ........ 102
Week 5 - Session 1 ........ 105
Week 5 - Session 2 ........ 107

# HIIT training protocol

- **Duration of the protocol**: 6 months
- **Trainings days**: twice a week, Monday and Wendsday
- **Video duration** about 20 minutes, warm up 5 minutes, cool down 5 minutes, HIIT protocol 10-15 minutes.
- **Time sets**: 20’’ exercise - 20’’ rest (1st month), 20’’ exercise - 15’’ rest (2nd month), 20’’ exercise - 10’’ rest (3rd month), 30’’ exercise - 20’’ rest (4th month), 40’’ exercise - 15’’ rest (5th and 6th month)
- Exercises are different every day, and the intensity of the exercises is increased between sessions in the same week. Exercise load, warm-up and stretching are changed every 4 weeks

| HIIT | Time sets | Nº Exercises | Duration | Sets | Rest between sets |
|---|---|---|---|---|---|
| 1st month | 20/20 | 4 | 20 min. | 4 | 1 min. |
| 2nd month | 20/15 | 4 | 20 min. | 4 | 1 min. |
| 3rd month | 20/10 | 4 | 17 min. | 4 | 1 min. |
| 4th month | 30/20 | 4 | 20 min. | 4 | 1 min. |
| 5th month | 40/15 | 4 | 20 min. | 4 | 1 min. |
| 6th month | 40/15 | 4 | 20 min. | 4 | 1 min. |

# Month 1 (20" of work - 20" of recovery)

## Common Part

*Dynamic Warm-up (At the beginning of each session before the main part) (Link)*

| Exercise | Photograph |
| --- | --- |
| Neck flexion and extension | 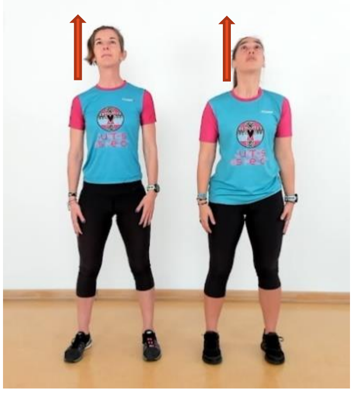 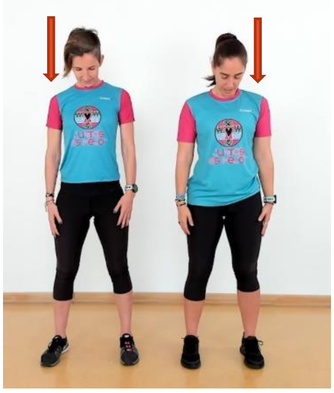 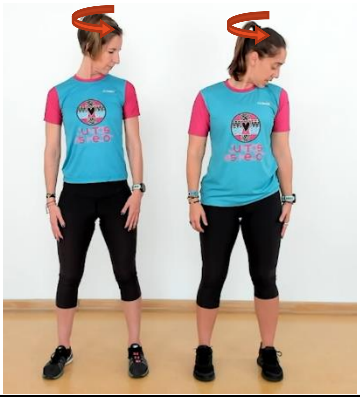 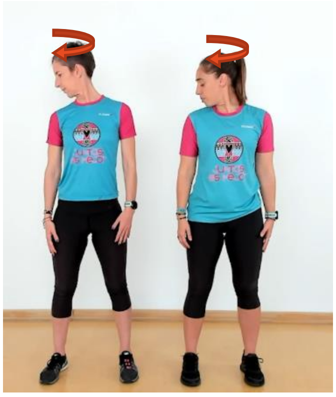 |
| Shoulder rotation with outstretched arms | 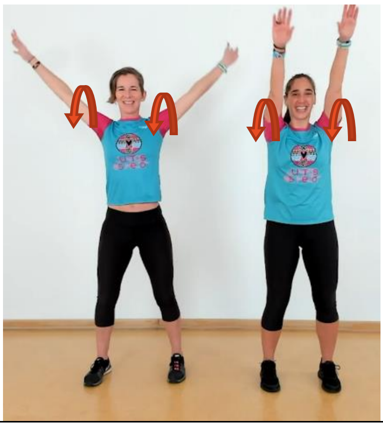 |

| | | |
|---|---|---|
| Hip rotation | 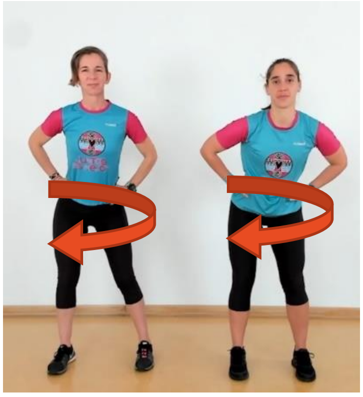 | 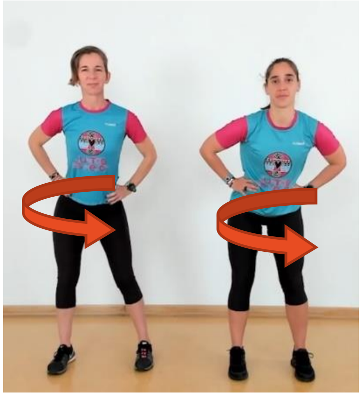 |
| Knee flexion and extension | 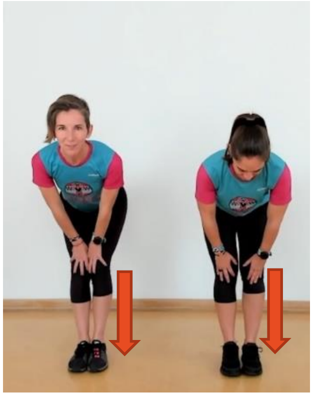 | 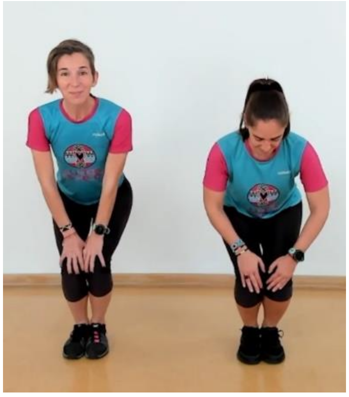 |
| Ankle rotation | 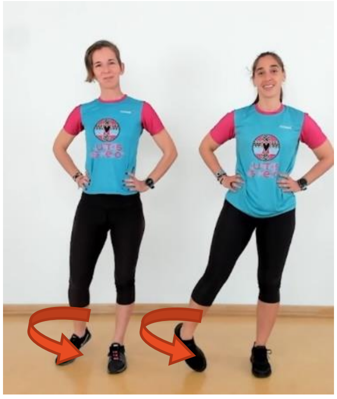 | 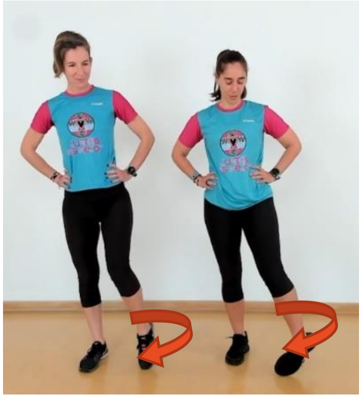 |

| | |
|---|---|
| Taps: front, back, side, side. | 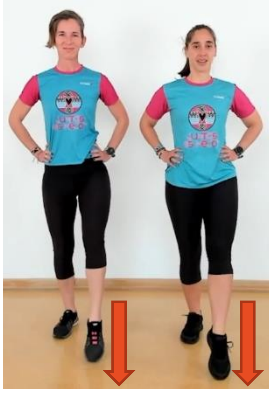 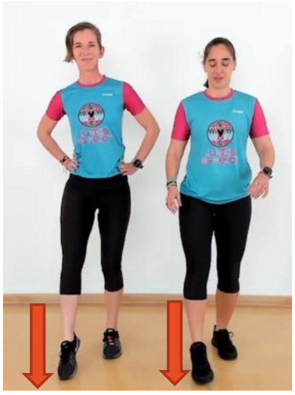 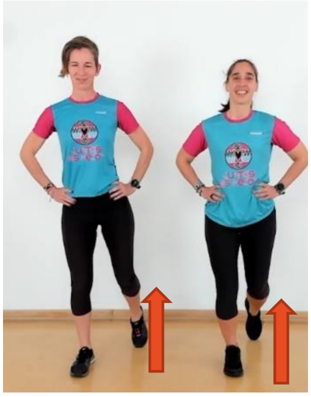 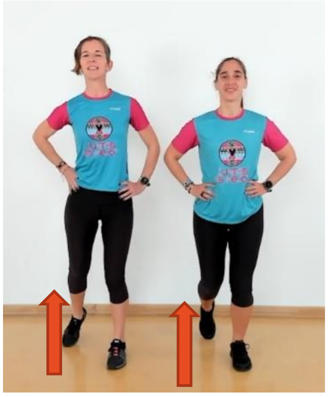 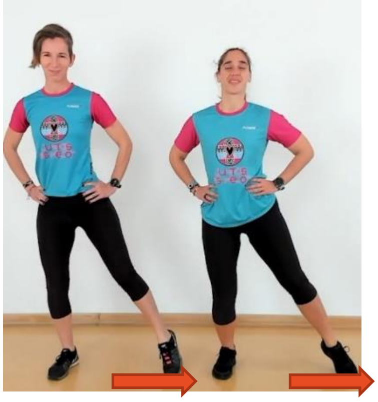 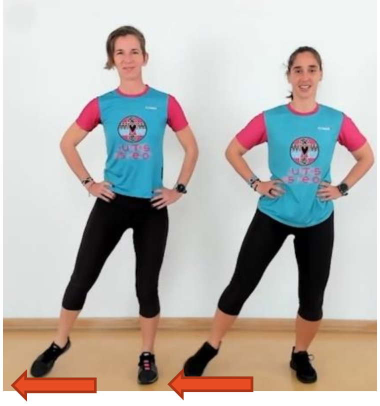 |
| Knees up | 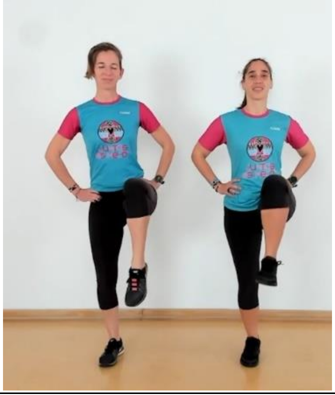 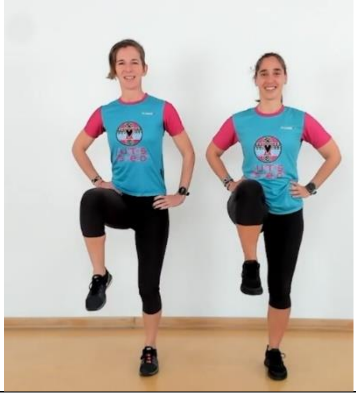 |

| | |
|---|---|
| Heels up | 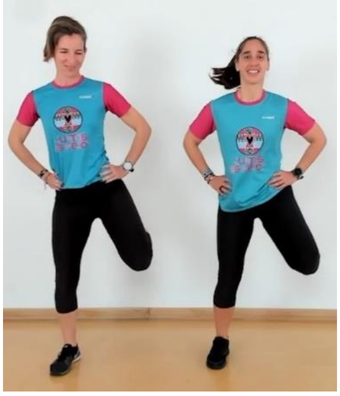 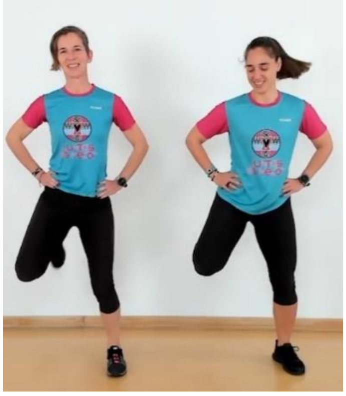 |

## *Stretching (At the end of each session after the main part) (Link)*

| Stretch | Photograph |
|---|---|
| Lateral and frontal neck lift | 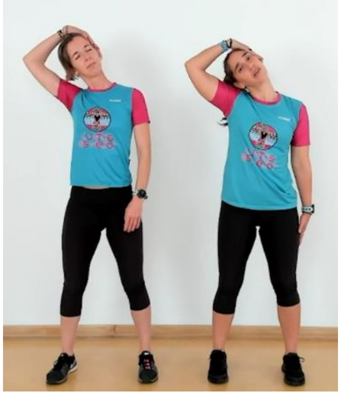 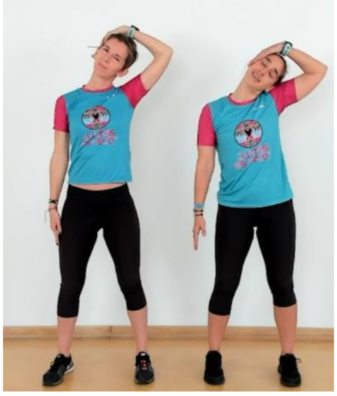 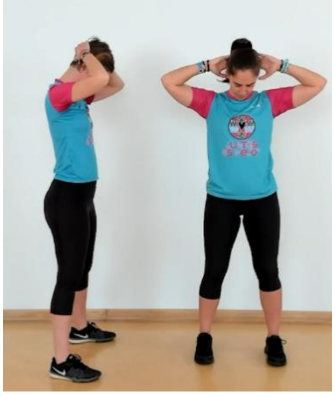 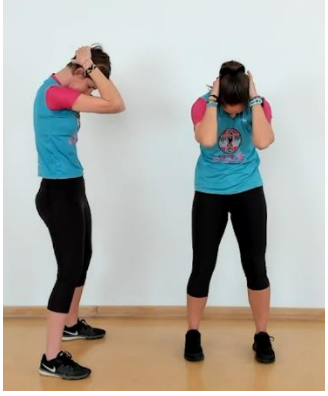 |
| Lateral tilt of spine with hands behind the neck | 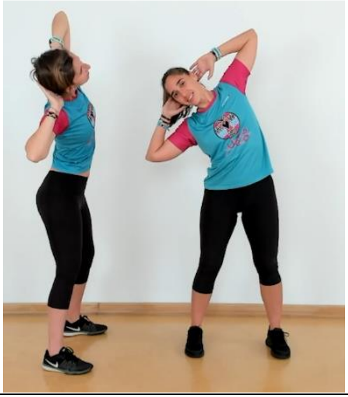 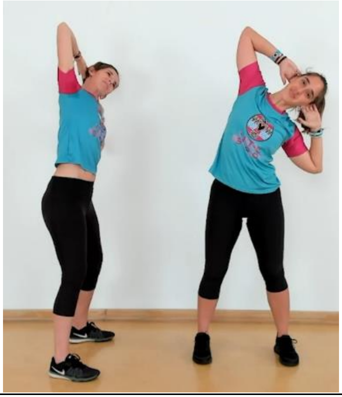 |
| Forearm stretch | 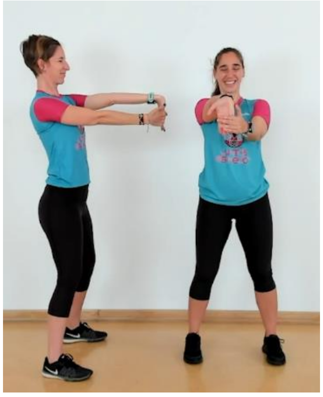 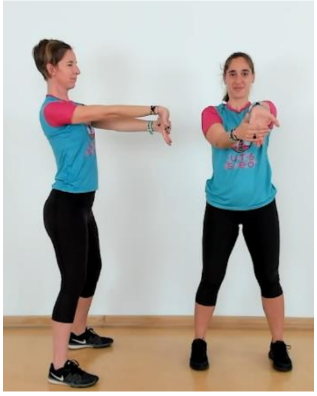 |

| | |
|---|---|
| Flexion and extension of the spine | 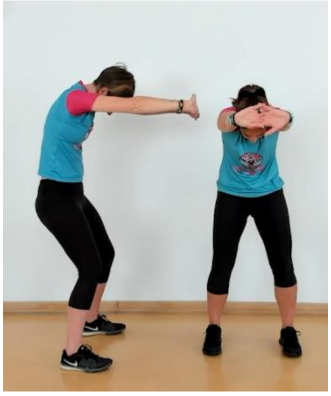 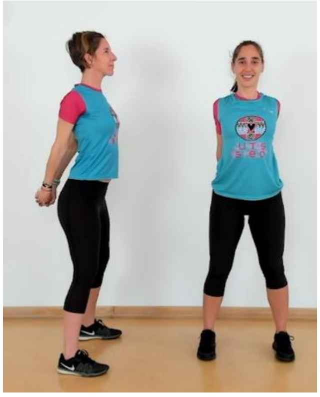 |
| Stretching of biceps femoris going up in flexion of the spine | 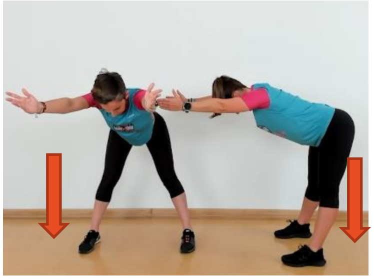 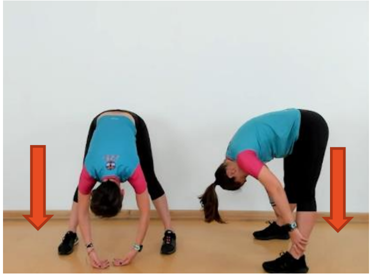 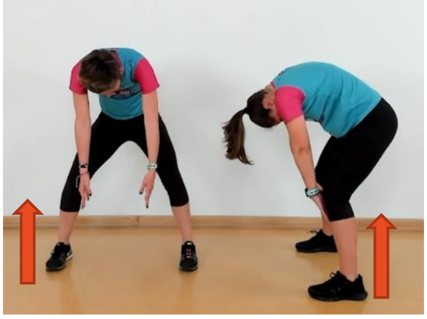 |
| Abductor stretch<br><br>Stretching of biceps femoris and triceps sural. | 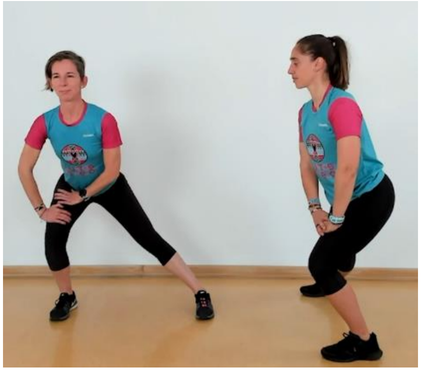 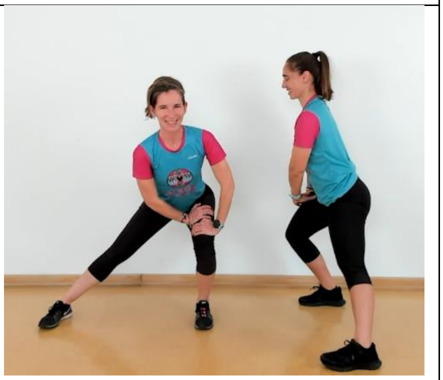 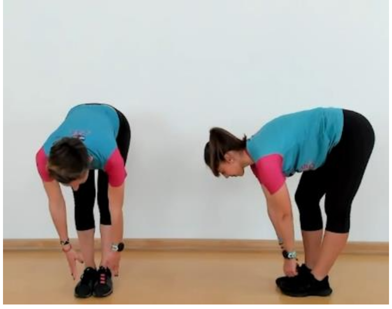 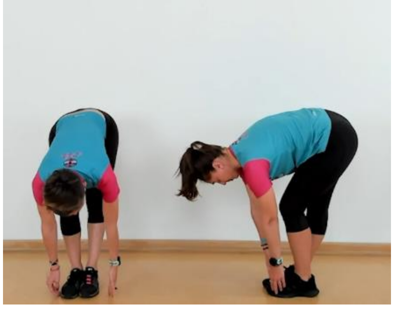 |

# Main part (4 rounds of 4 exercises with 1’ rest in between rounds) (20’’ Work – 20’’ Rest)

*Week 1 - Session 1 (Link)*

| Exercise | Photo |
|---|---|
| Skipping | 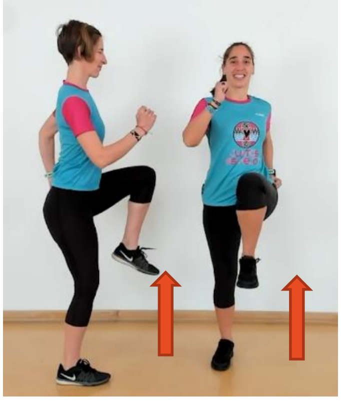 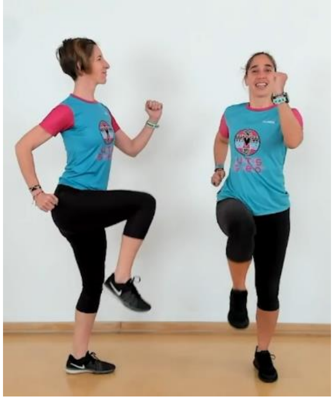 |
| Squat | 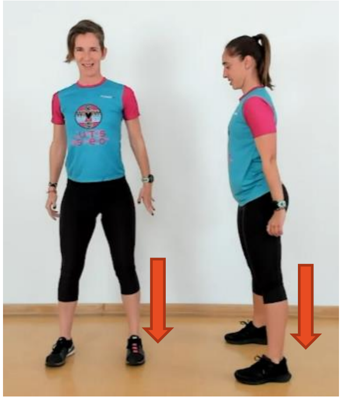 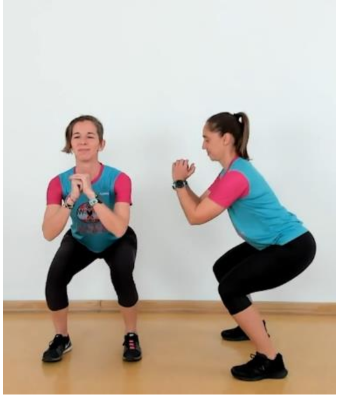 |
| Press militar | 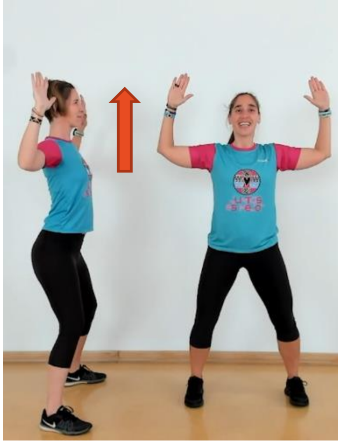 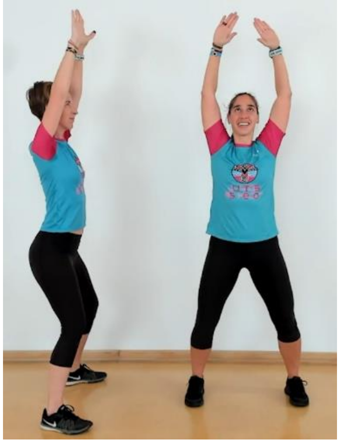 |

| | |
|---|---|
| Lateral tilt (4 to one side 4 to the other) | 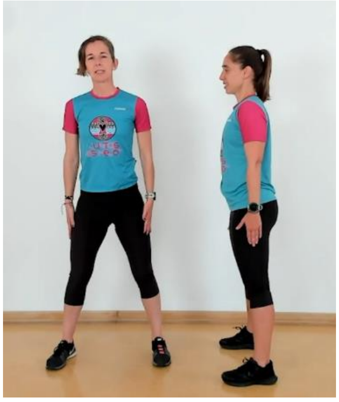 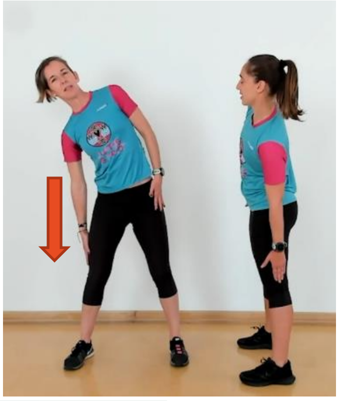 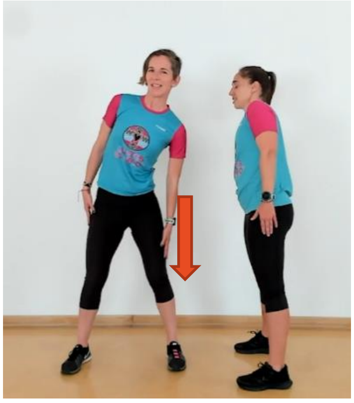 |

## *Week 1 - Session 2 (Link)*

| Exercise | Photo |
|---|---|
| Skipping + arm extension | 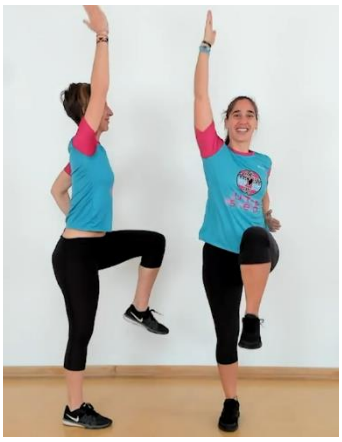 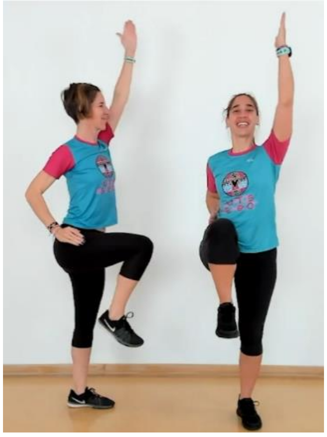 |
| Squat + rise up on your toes | 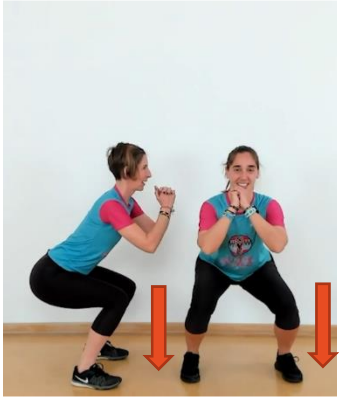 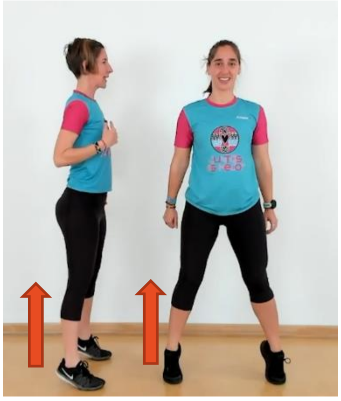 |
| Shoulder extension with outstretched arms | 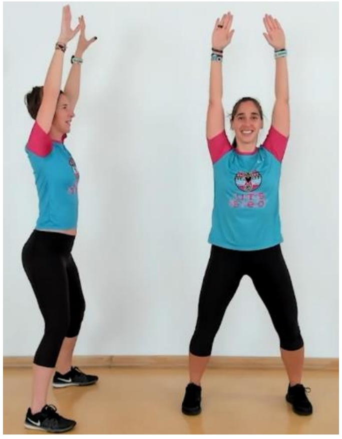 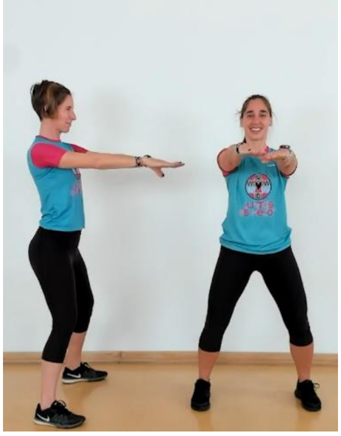 |
| Double tilt | 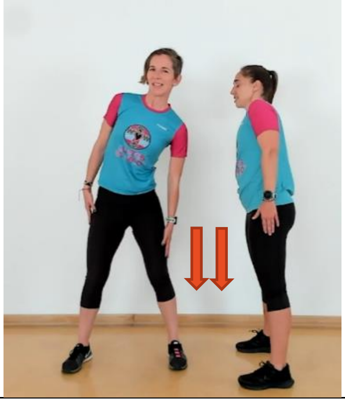 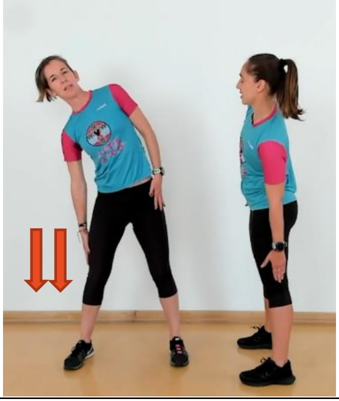 |

## *Week 2 - Session 1 (Link)*

| Exercise | Photo |
|---|---|
| Tap side to side | 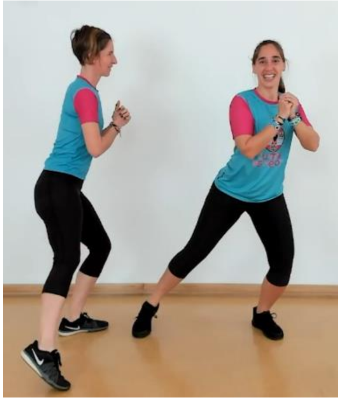 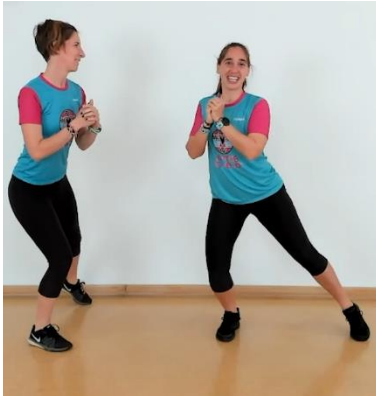 |
| Lunge | 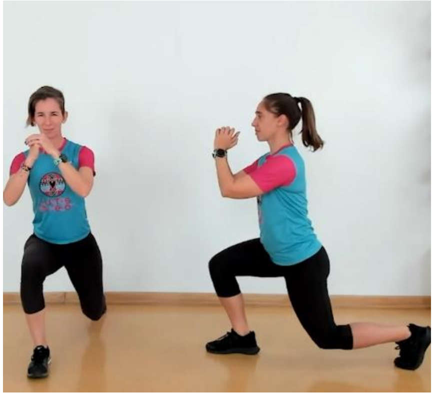 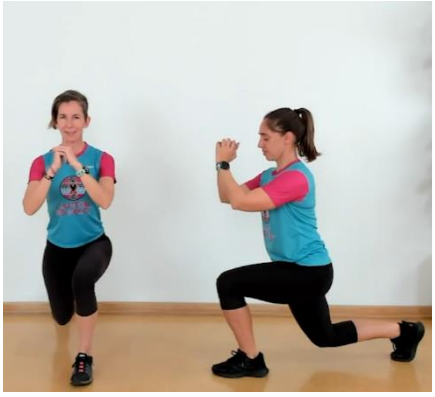 |
| Arms Rotation | 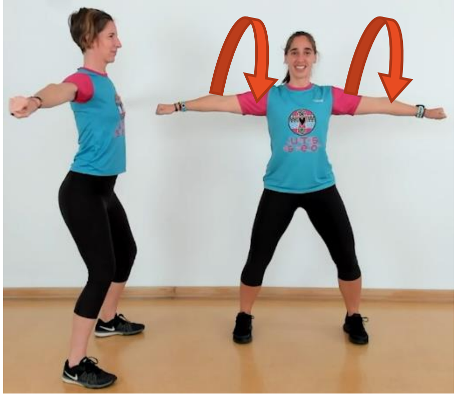 |
| Side shrinks | 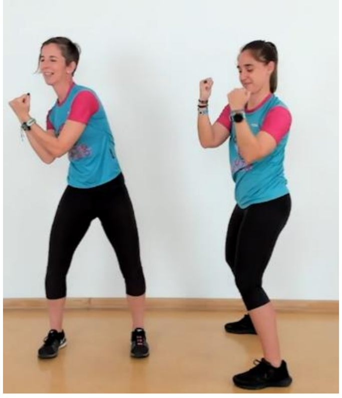 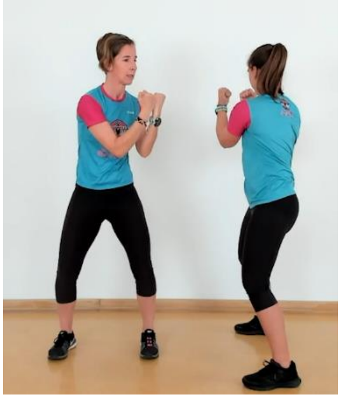 |

## *Week 2 - Session 2 (Link)*

| Exercise | Photo |
|---|---|
| Dynamic tap side to side | 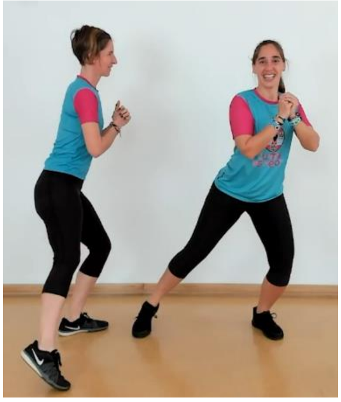 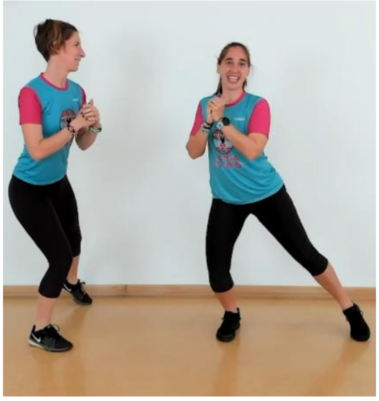 |
| Double Lunge | 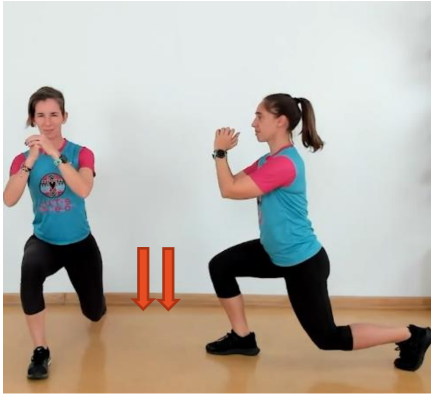 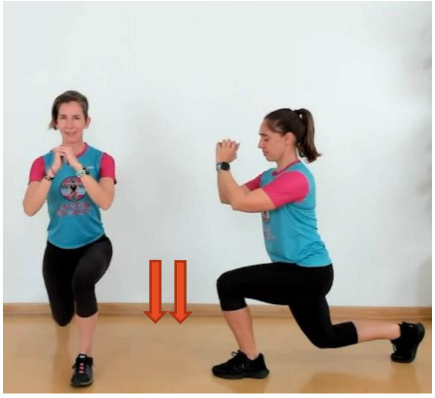 |
| Arms Rotation - Forward and backwards | 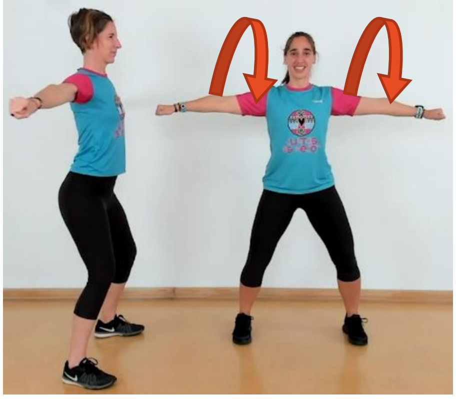 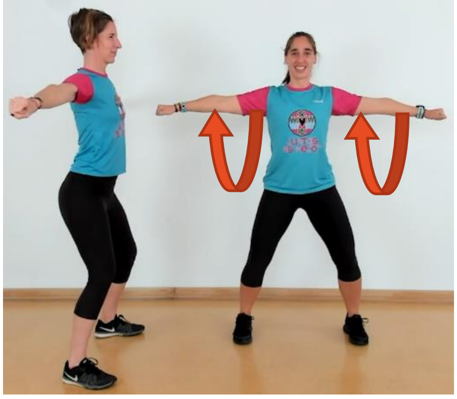 |
| Double side shrinks | 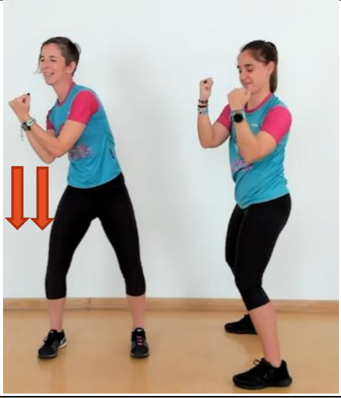 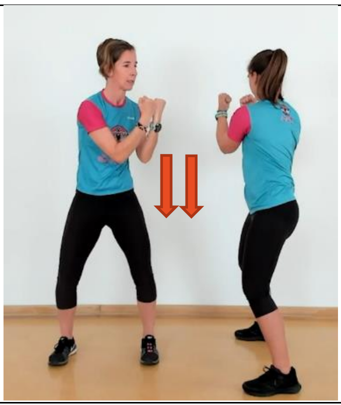 |

## *Week 3 - Session 1 (Link)*

| Exercise | Photo |
|---|---|
| Kicks | 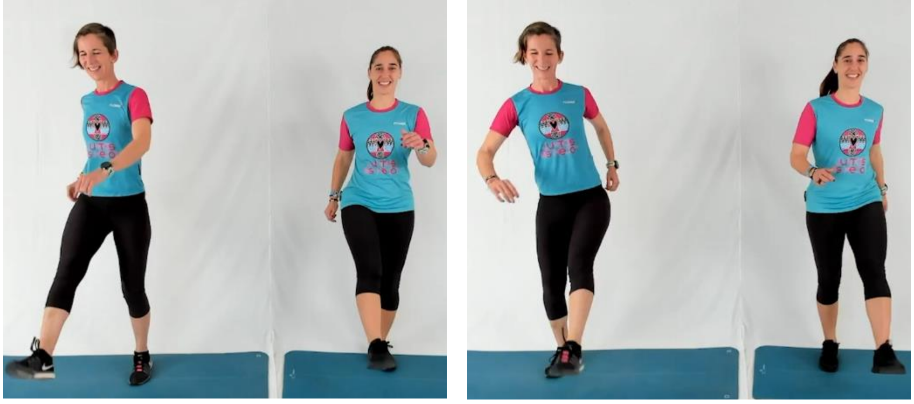 |
| Double Squat | 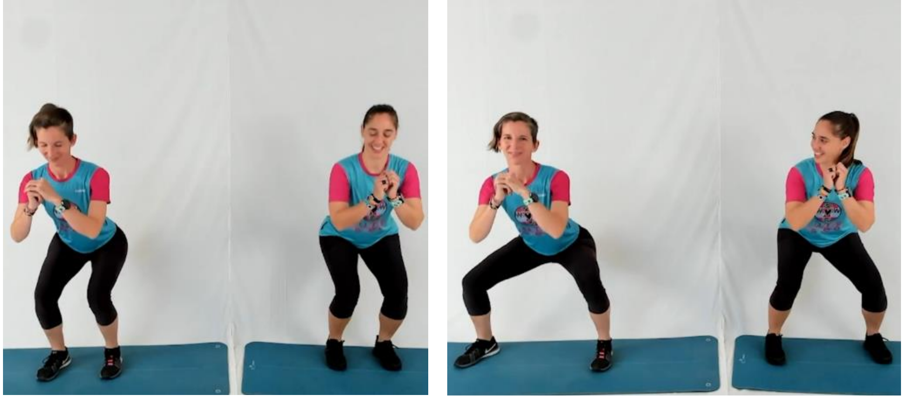 |
| Arms cross | 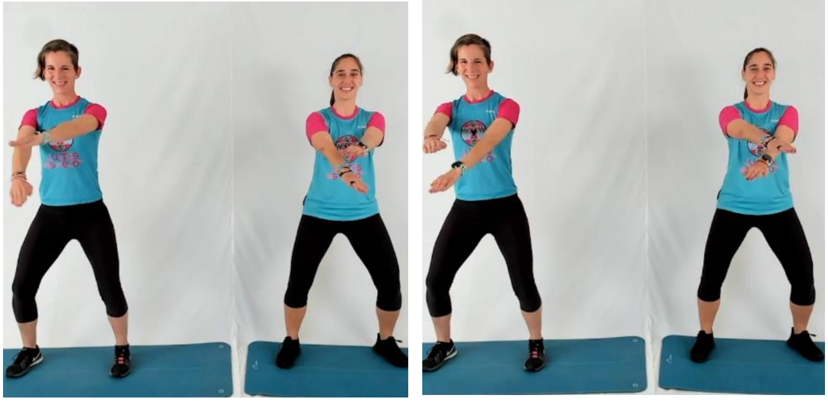 |
| Leg twist | 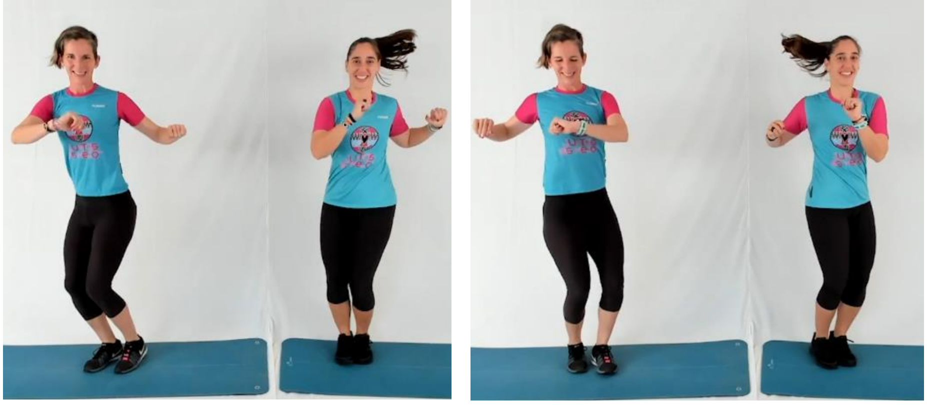 |

## *Week 3 - Session 2 (Link)*

| Exercise | Photo |
|---|---|
| Knee and kick | 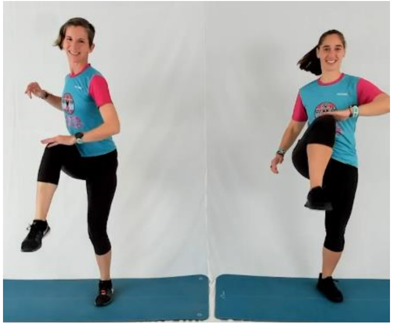 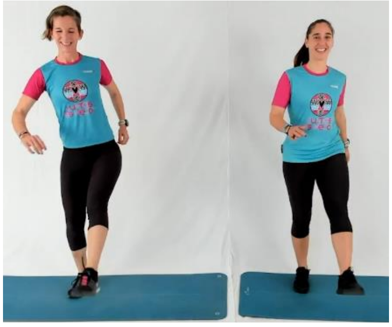 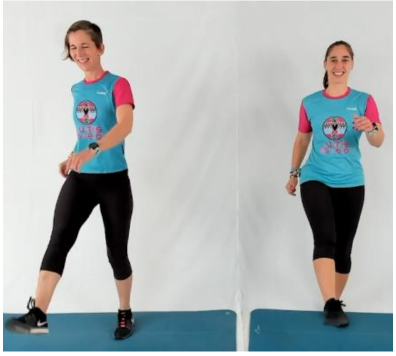 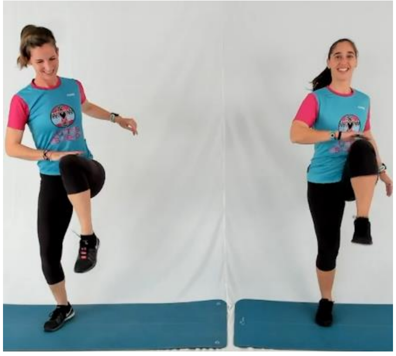 |
| 3 timed squats | 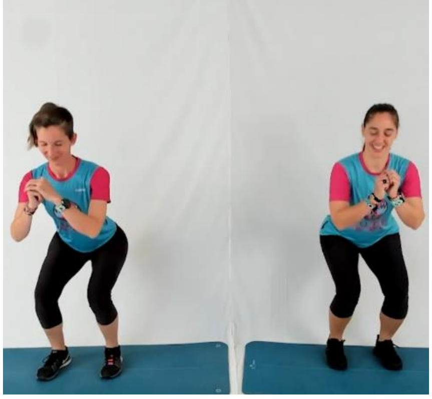 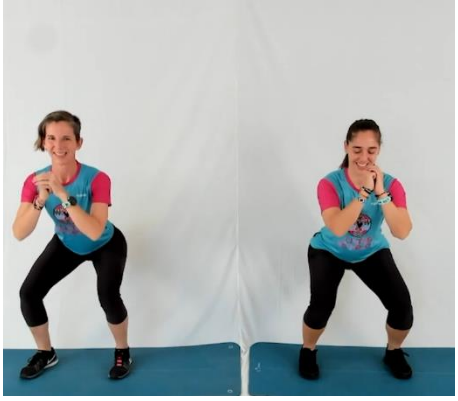 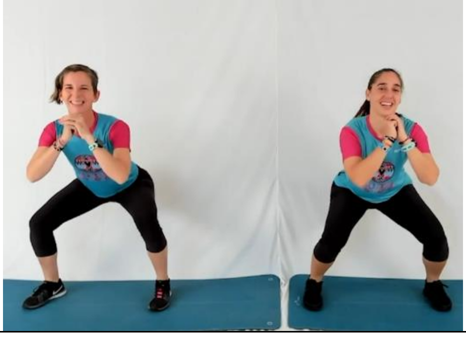 |

| | |
|---|---|
| Up and down - Arms cross | 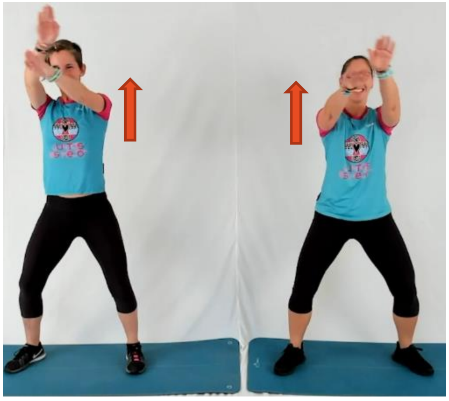 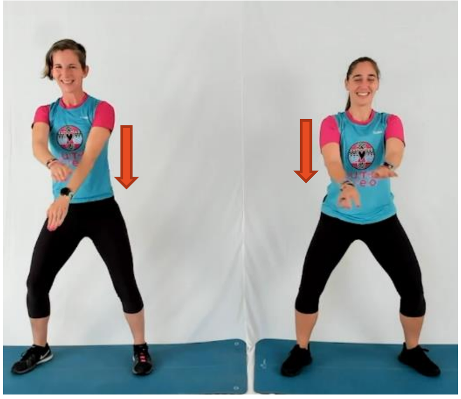 |
| Jumping Leg twist | 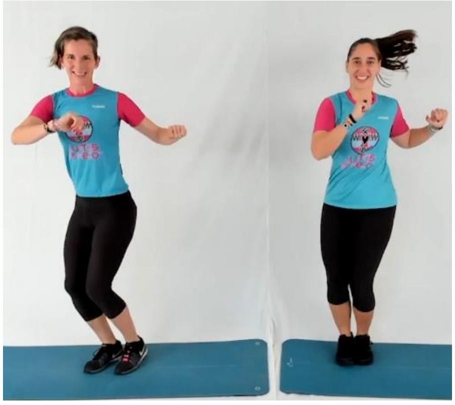 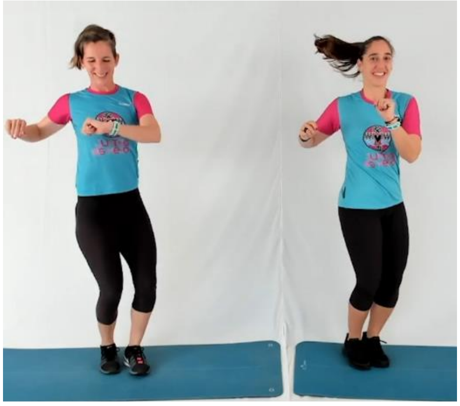 |

## *Week 4 - Session 1 ([Link](Link))*

| Exercise | Photo |
|---|---|
| Skipping | 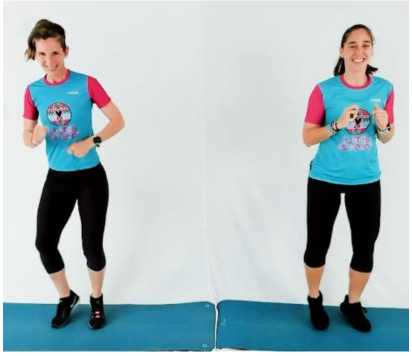 |
| Squat and heel tap forward | 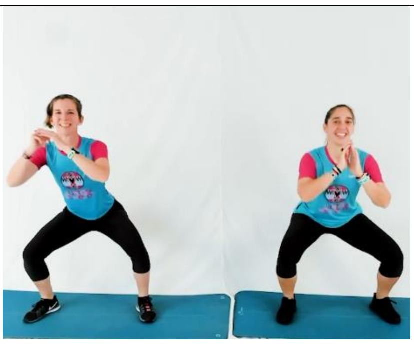 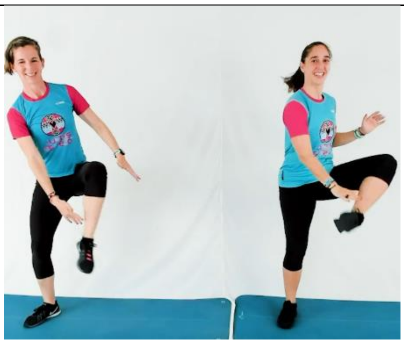 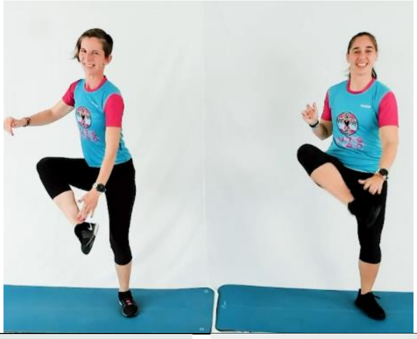 |
| Punches | 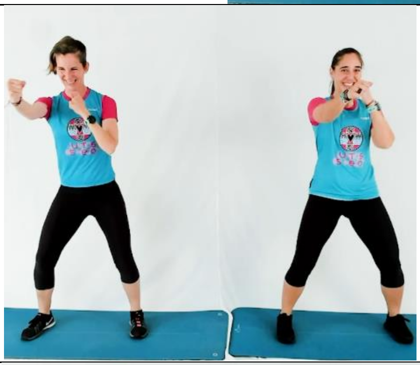 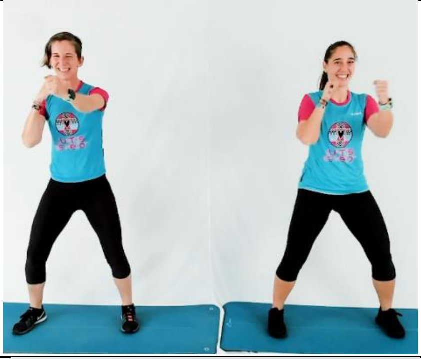 |
| Side crunches | 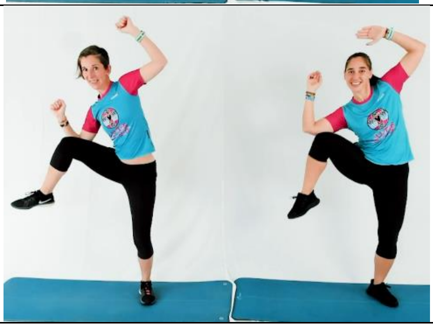 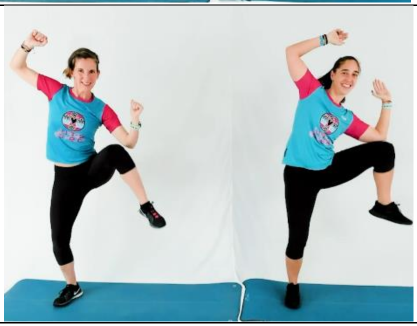 |

## *Week 4 - Session 2 (Link)*

| Exercise | Photo |
|---|---|
| In and out Skipping | 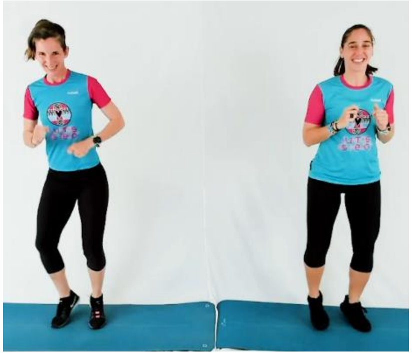 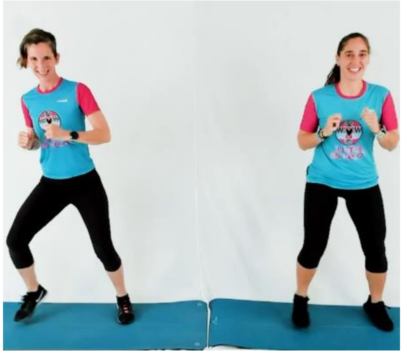 |
| Squat forward and backward heel tap (once with each leg) | 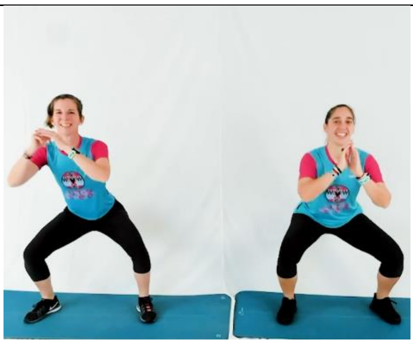 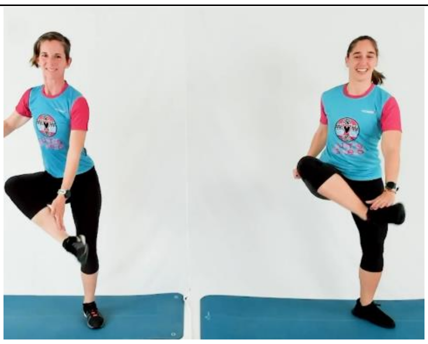 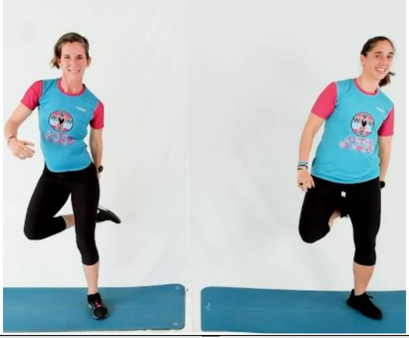 |
| Double punches | 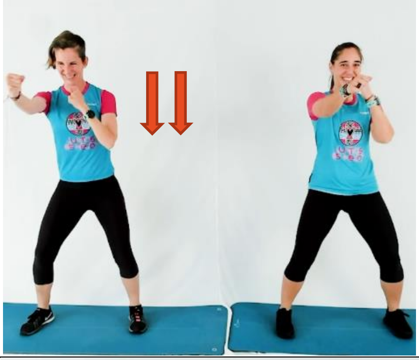 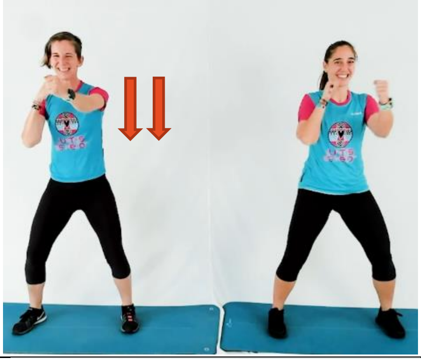 |
| Double side crunches | 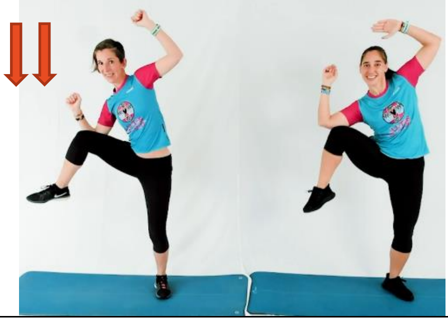 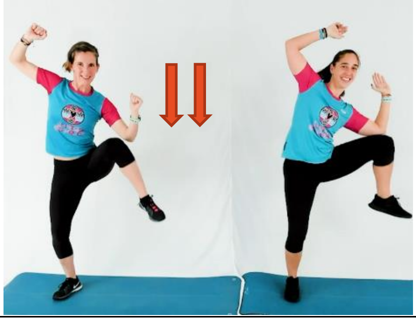 |

# Month 2 (20" of work - 15" of recovery)

## Common Part

*Dynamic Warm-up (At the beginning of each session before the main part) (Link)*

| Exercise | Photograph |
|---|---|
| Neck twisting | 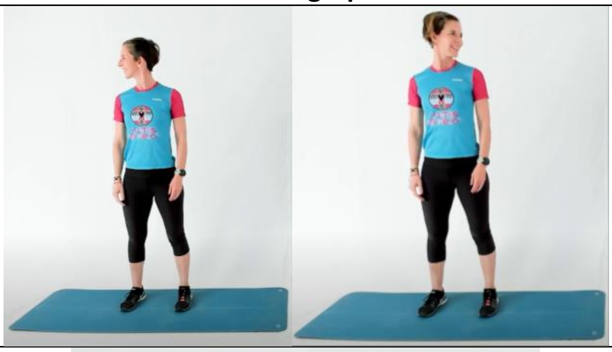 |
| Neck flexion and extension | 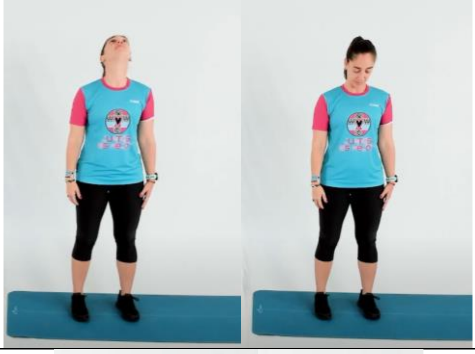 |
| Front and back shoulder rotation | 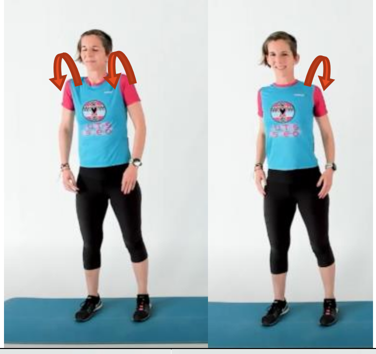 |
| Shoulder rotation | 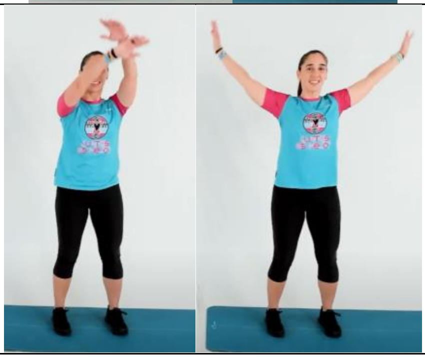 |

| | |
|---|---|
| Abduction and adduction of arms (ballistic) | 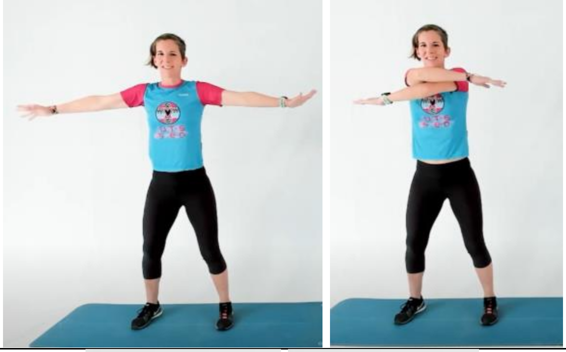 |
| Hip rotation | 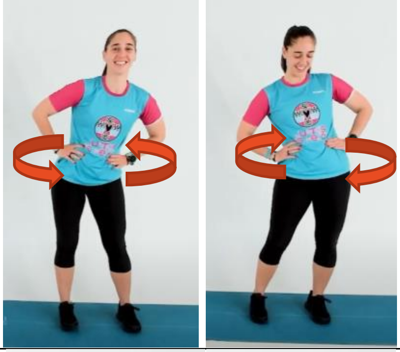 |
| knee flexion and extension | 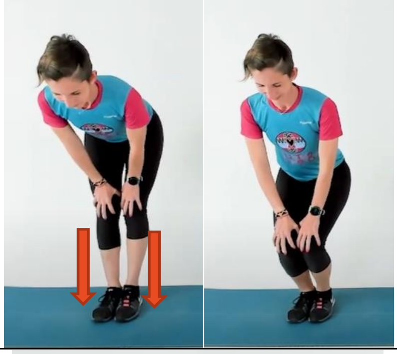 |
| Ankle rotation | 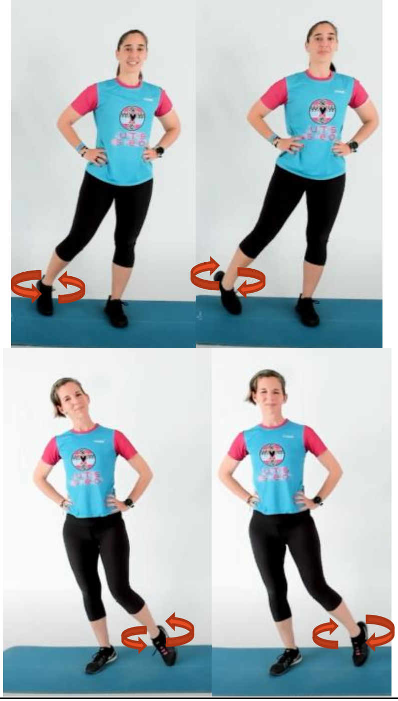 |

| | |
|---|---|
| Taps: front, back, left, right | 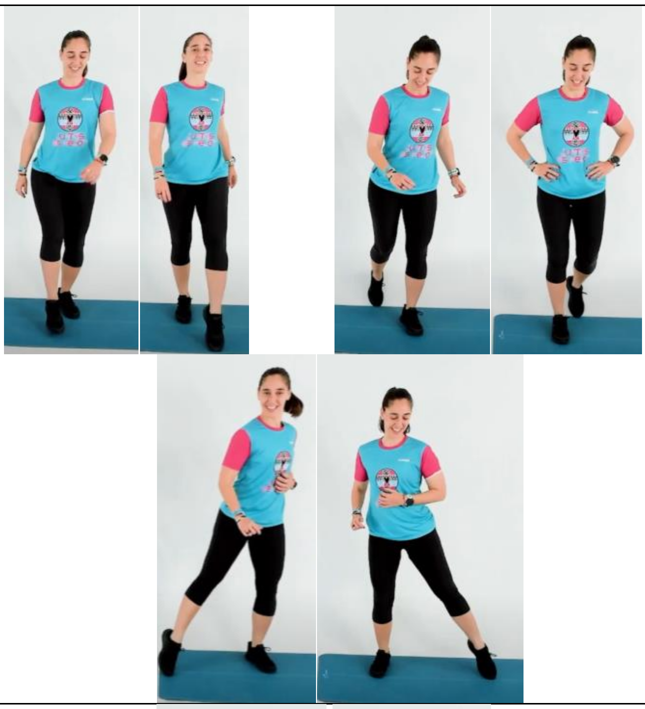 |
| Knee lift | 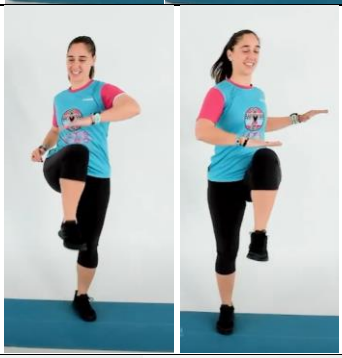 |
| Back heels | 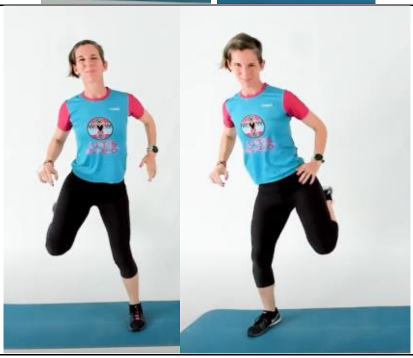 |

*Stretching (At the end of each session after the main part) (Link)*

| Stretch | Photograph |
|---|---|
| Stretching with bending + twisting | 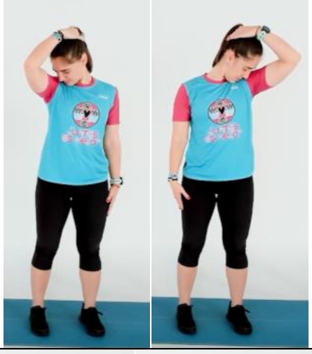 |
| Assisted shoulder stretch with crossed arm in front | 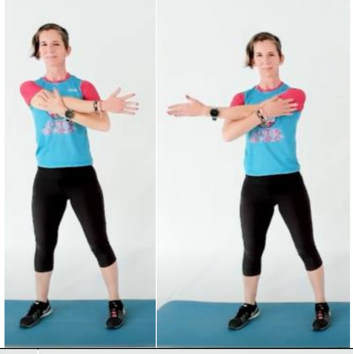 |
| Lateral spine tilt with arms extended | 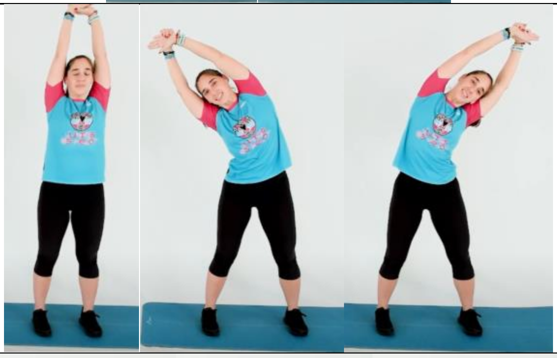 |
| Posterior chain stretch: Hip flexion + spinal rotation with extended arm | 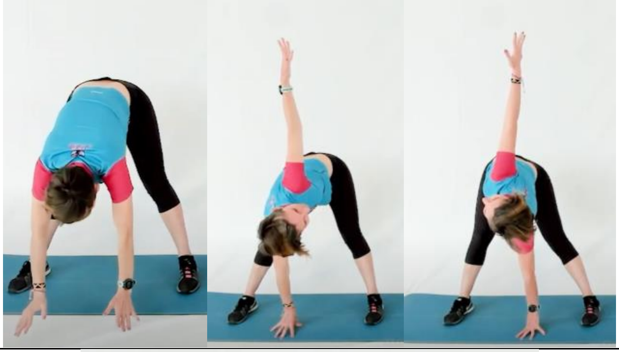 |
| Cat Camel with hands on knee | 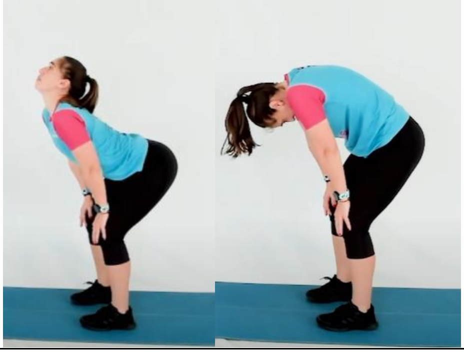 |

## Main part (4 rounds of 4 exercises with 1’ rest in between rounds) (20’’ Work – 15’’ Rest)

### *Week 1 - Session 1 (Link)*

| Exercise | Photo |
|---|---|
| Taps | 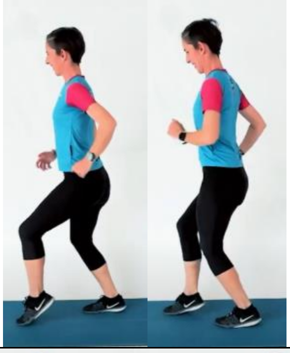 |
| Skater | 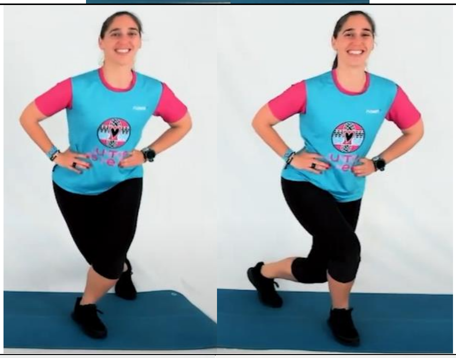 |
| Shoulder back rotation with extended arm | 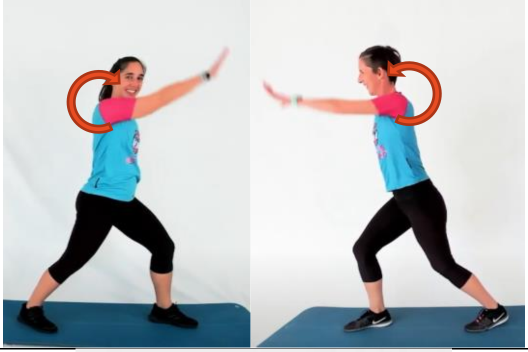 |
| Lateral shrinkage of abdomen with arms in front | 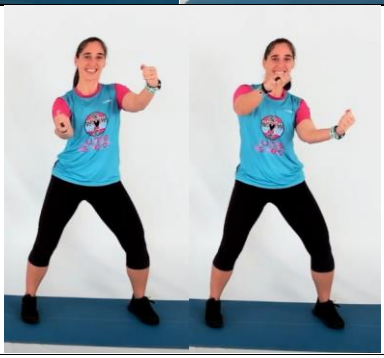 |

## *Week 1 - Session 2 (Link)*

| Exercise | Photo |
| --- | --- |
| Scissor lunge | 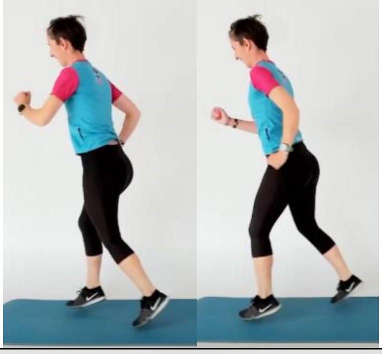 |
| Skater + abduction with extended leg | 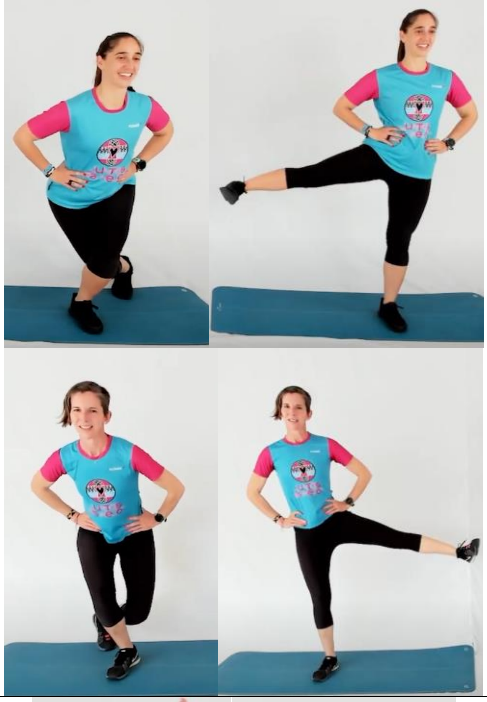 |
| Shoulder flexion and extension with extended arm | 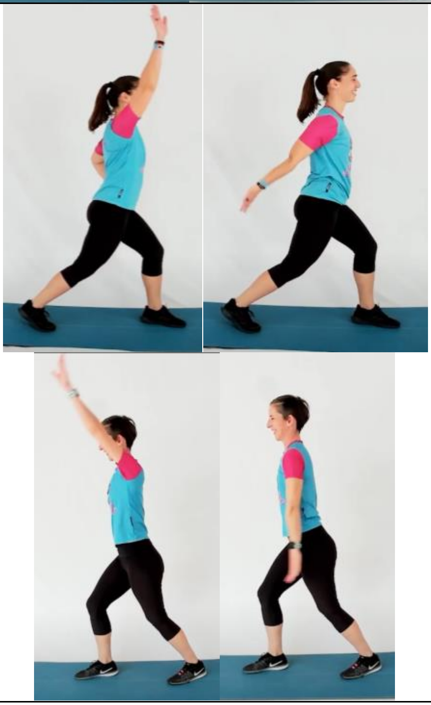 |

| | |
|---|---|
| Lateral Abdominal Shrinkage + Knee Lift | 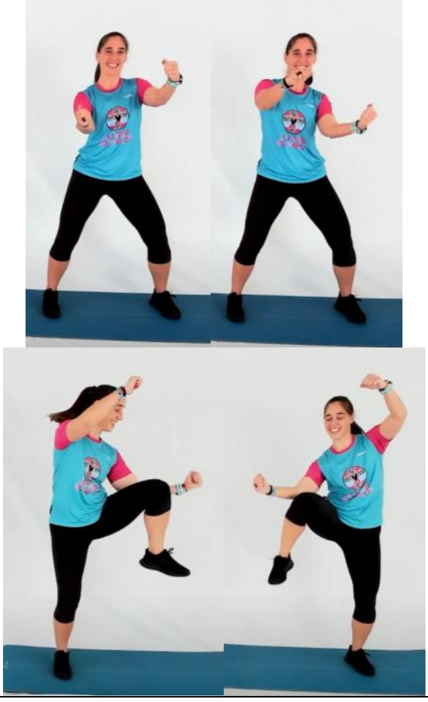 |

*Week 2 - Session 1 (Link)*

| Exercise | Photo |
|---|---|
| Jumping | 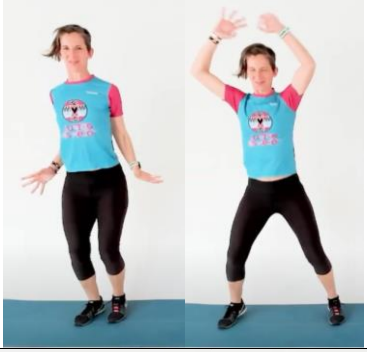 |
| Lunge + Front knee lift with jump | 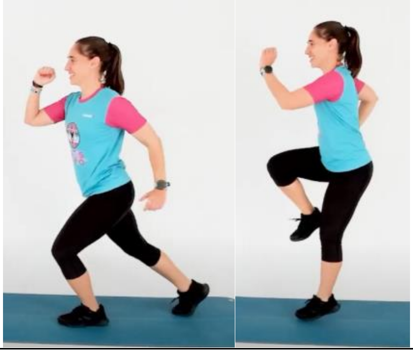 |

| | |
|---|---|
| Arm opening with 90º angle | 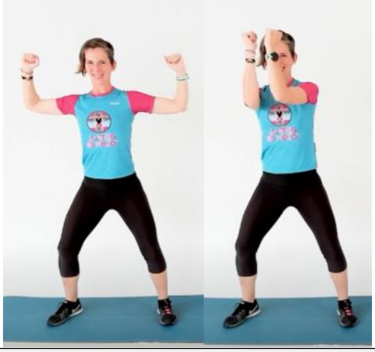 |
| Plank | 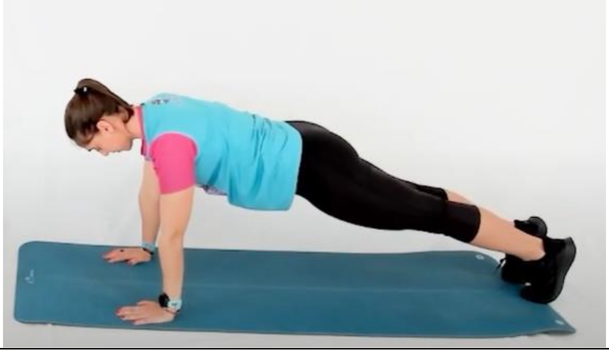 |

## *Week 2 - Session 2 (Link)*

| Exercise | Photo |
|---|---|
| JUMPING +3 Rebounds | 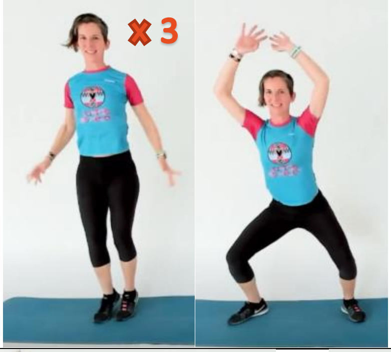 |
| Scissor + lunge + Frontal knee lift | 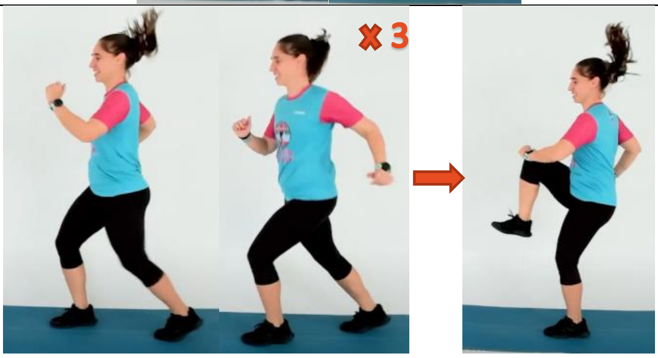 |

| | |
|---|---|
| | 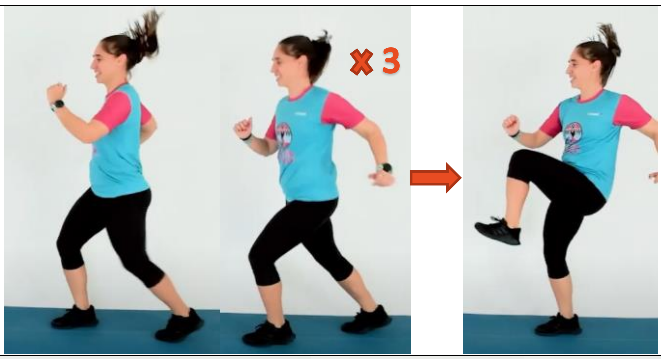 |
| Arm opening with 90º angle + front arm extension | 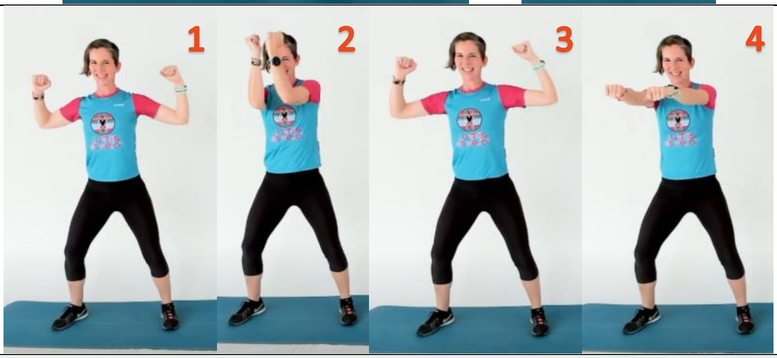 |
| Elbow Plank | 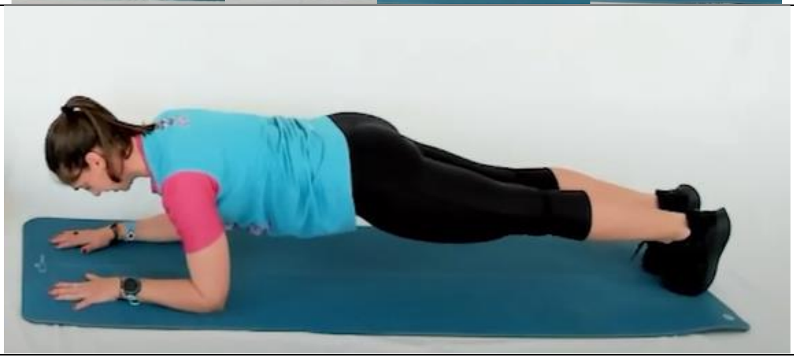 |

## *Week 3 - Session 1 (Link)*

| Exercise | Photo |
|---|---|
| Frontal kick + Frontal knee lift | 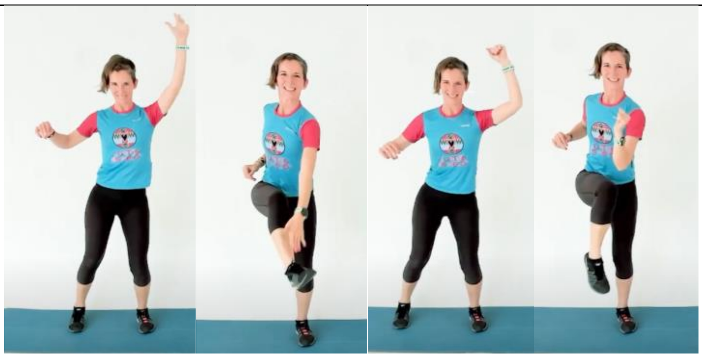 |

| | |
|---|---|
| | 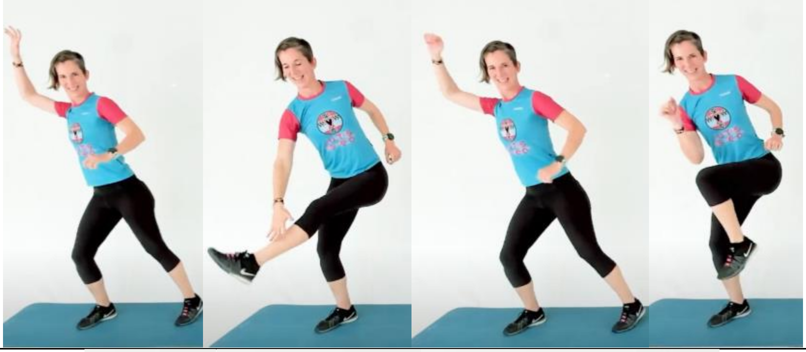 |
| Lunge in two times | 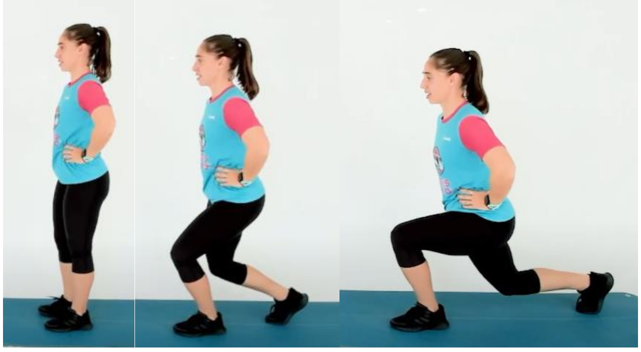 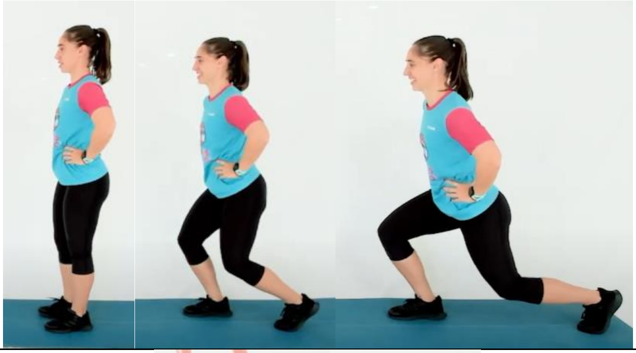 |
| Shoulder flexion and extension with extended arms | 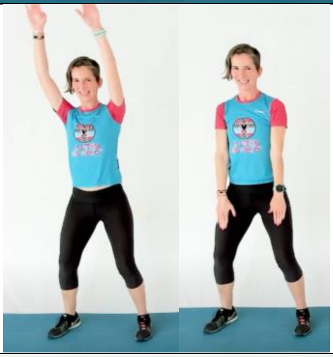 |
| Lateral plank + up-down hip | 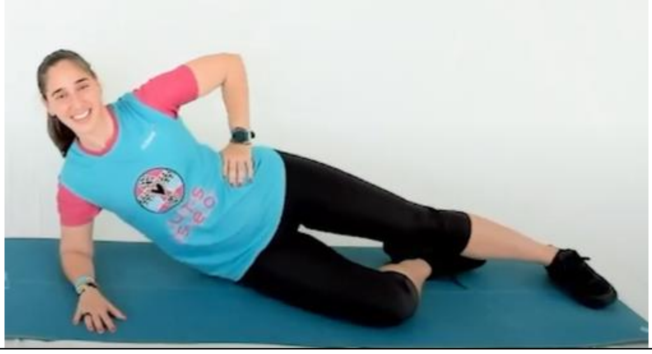 |

| | |
|---|---|
| | 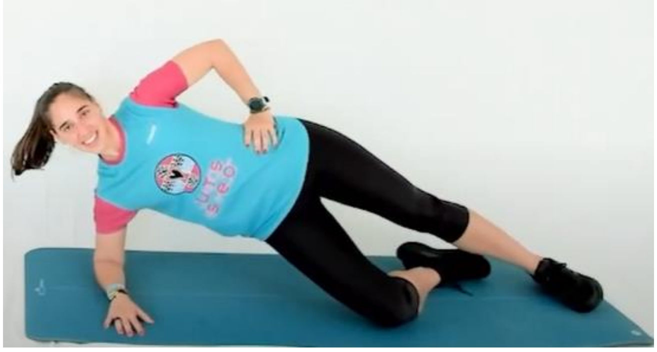 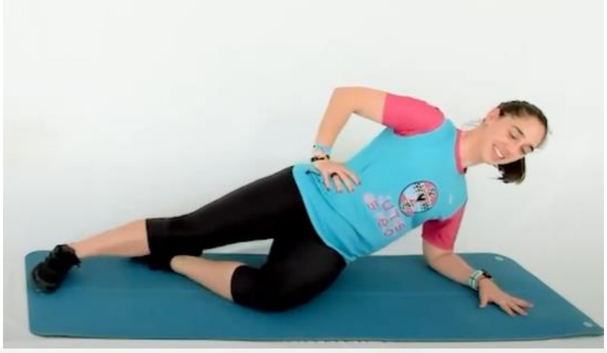 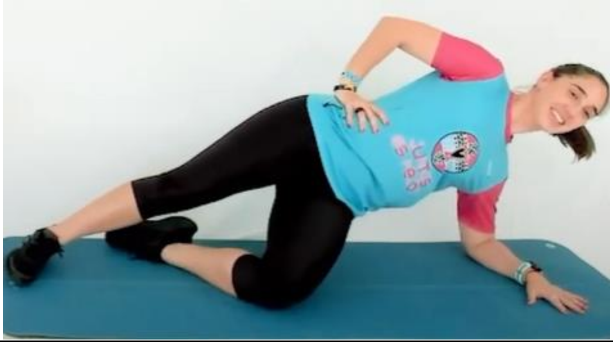 |

## *Week 3 - Session 2 (Link)*

| Exercise | Photo |
|---|---|
| Kick+ front knee lift + sprint | 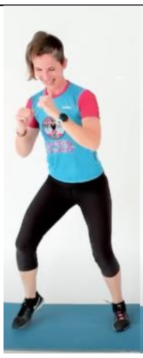 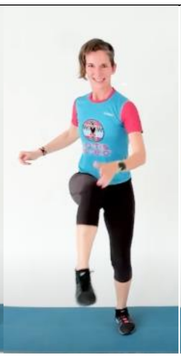 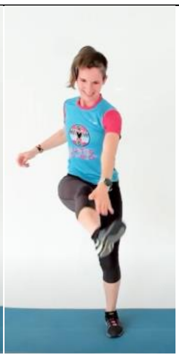 |

| | |
|---|---|
| | 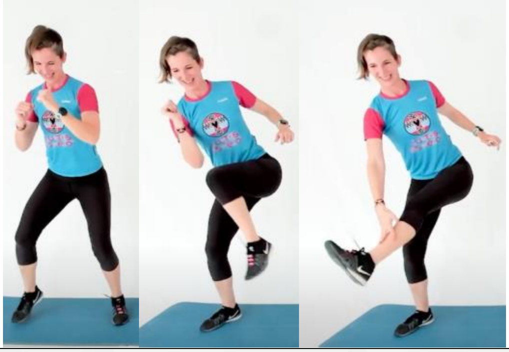 |
| Lunge 2 times go-2 times back | 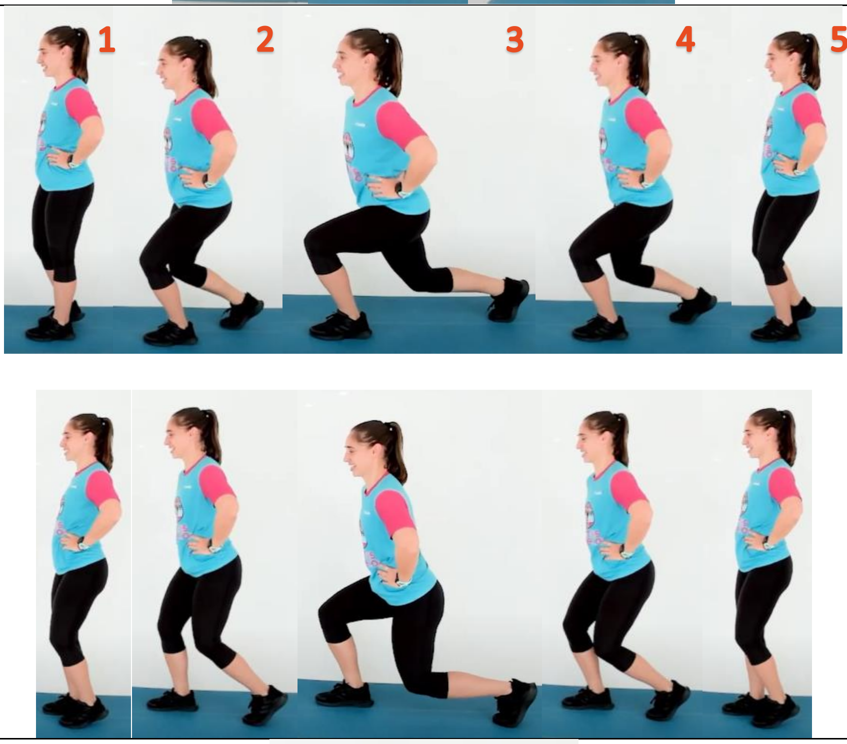 |
| Shoulder extension with extended arms + elbow flexion | 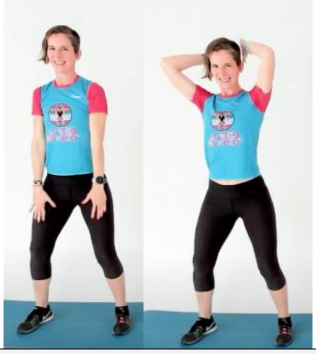 |
| Isometric side plank | 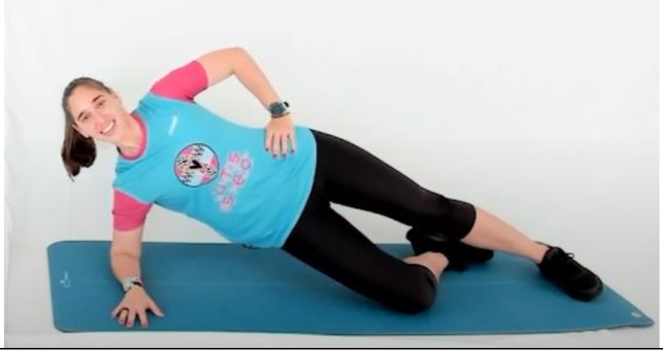 |

| | |
|---|---|
| | 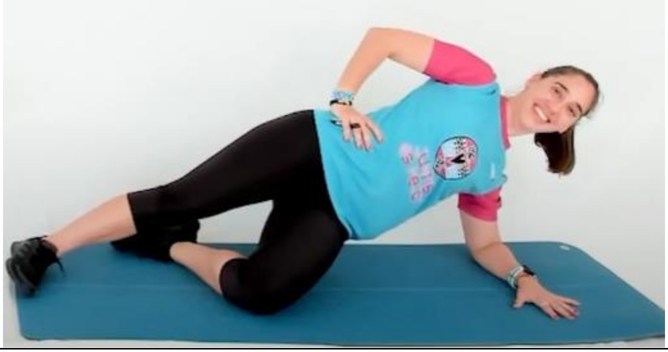 |

## *Week 4 - Session 1 (Link)*

| Exercise | Photo |
|---|---|
| Lateral sprint | 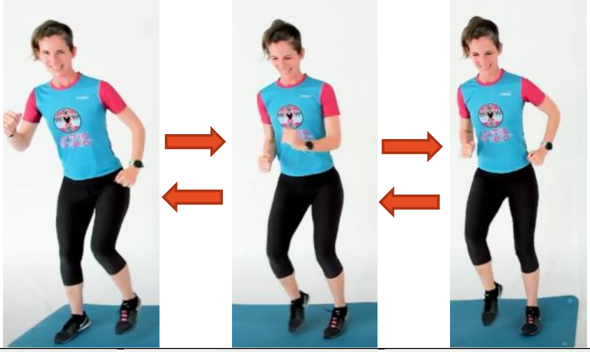 |
| Sumo squat + Alternate heel lift | 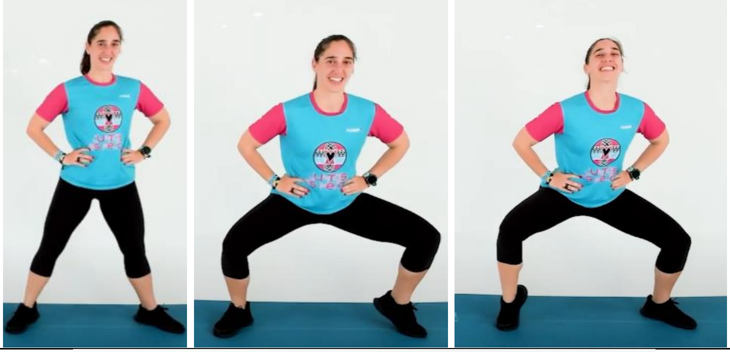 |
| Push up from four-footed position | 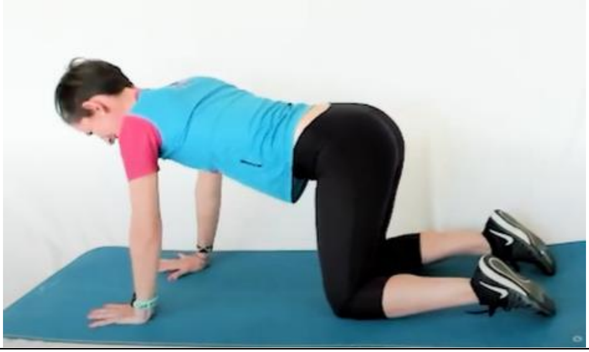 |

| | |
|---|---|
| | 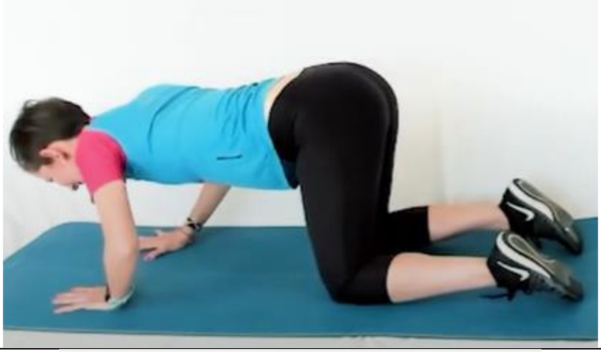 |
| Plank + Downward facing dog | 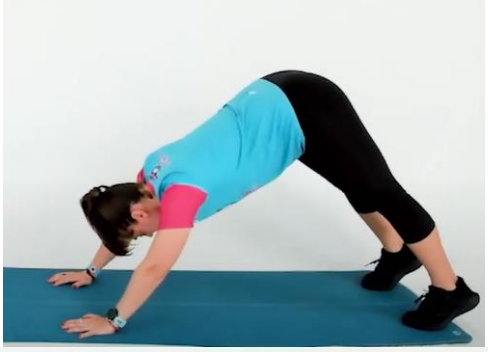 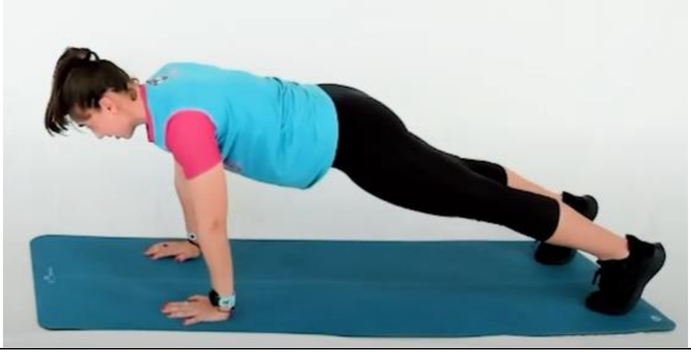 |

## *Week 4 - Session 2 (Link)*

| Exercise | Photo |
|---|---|
| Lateral sprint + knee lift | 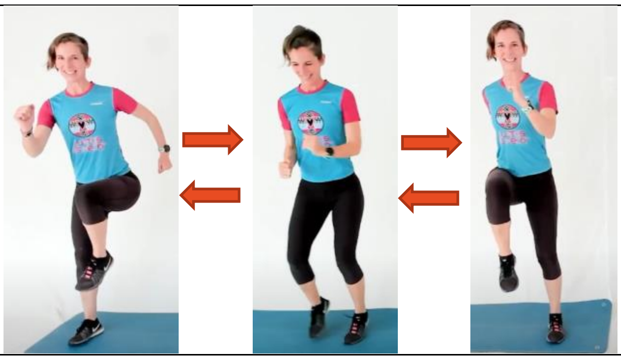 |

| | |
|---|---|
| Sumo Squat+ 2 heel lifts | 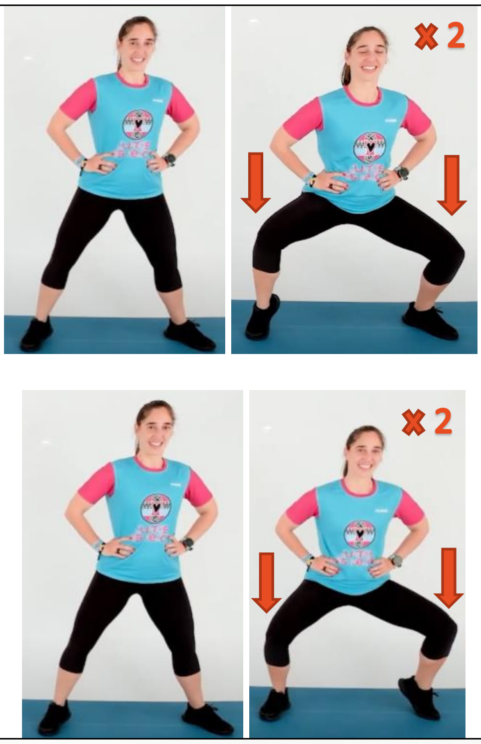  |
| Push up with extended leg from four-footed position | 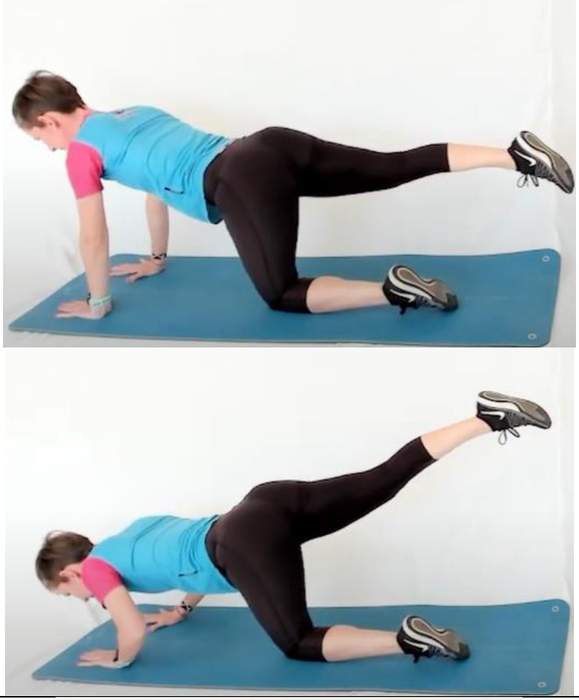 |
| Plank + Mountain climbing+ Downward facing dog | 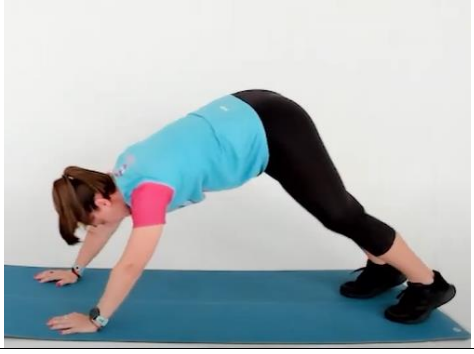 |

| | |
|---|---|
| | 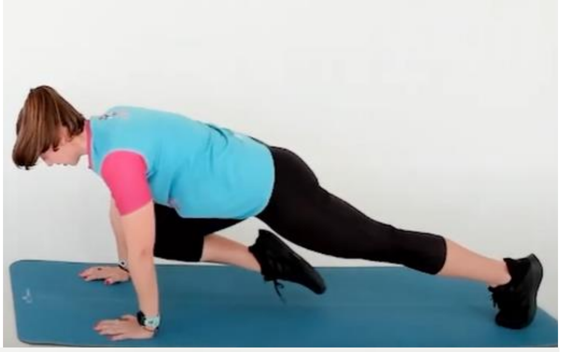 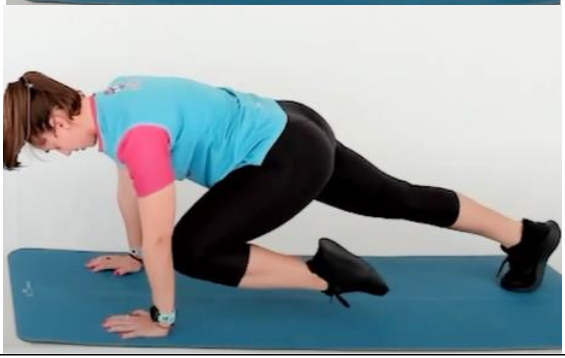 |

# Month 3 (20" of work - 10" of recovery)

## Common Part

*Dynamic Warm-up (At the beginning of each session before the main part) (Link)*

| Exercise | Photograph |
|---|---|
| Hip and spine flexion + hip extension + spine lift from lumbar to cervical | 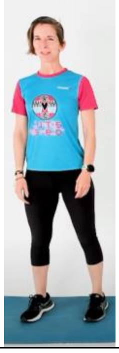 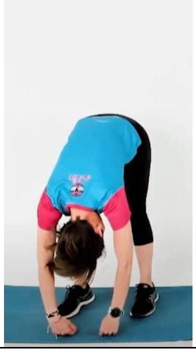 |

| | |
|---|---|
| Front-back shoulder rotation | 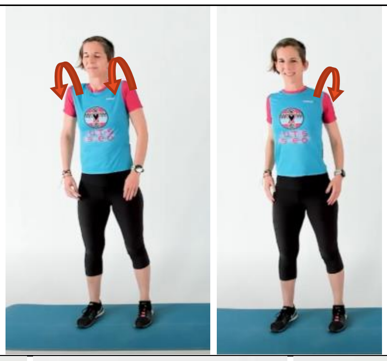 |
| Lateral swing with outstretched arms | 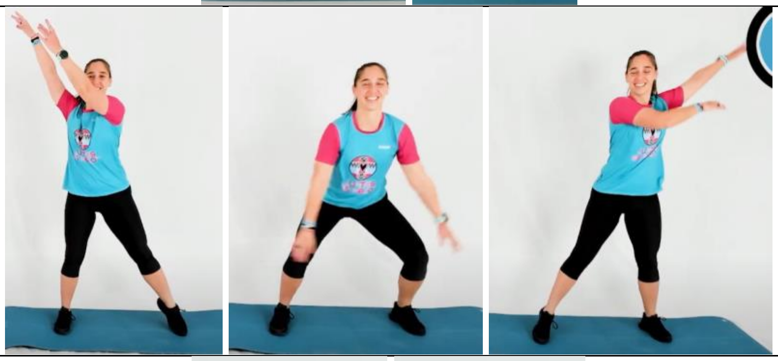 |
| Hip rotation | 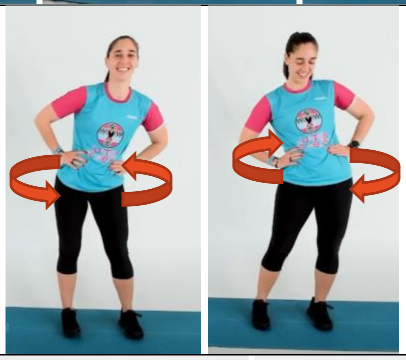 |
| Abduction + hip adduction with extended leg (ballistic) | 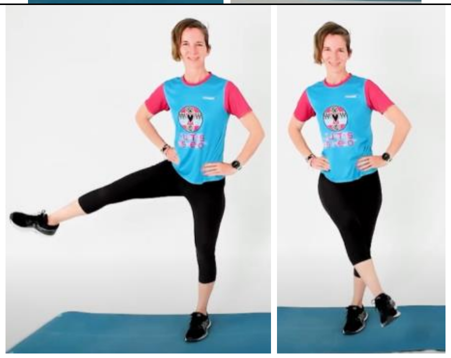 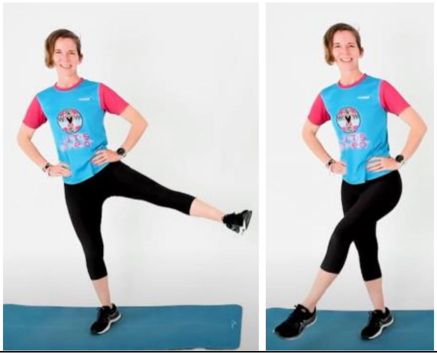 |

| | |
|---|---|
| March opening and closing | 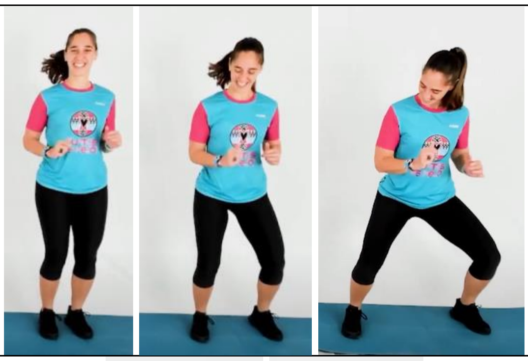 |
| Cross tap in front | 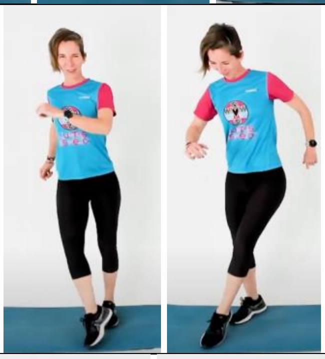 |
| Cross tap behind | 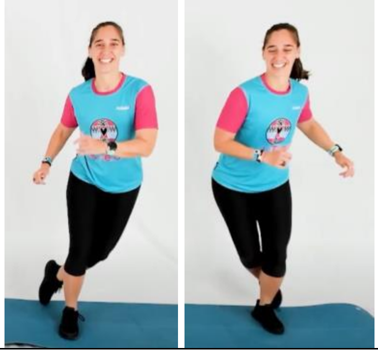 |

*Stretching (At the end of each session after the main part) (Link)*

| Stretch | Photograph |
|---|---|
| Triceps | 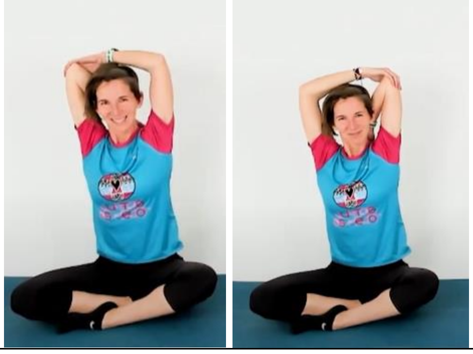 |

| | |
|---|---|
| Cat camel from sitting position | 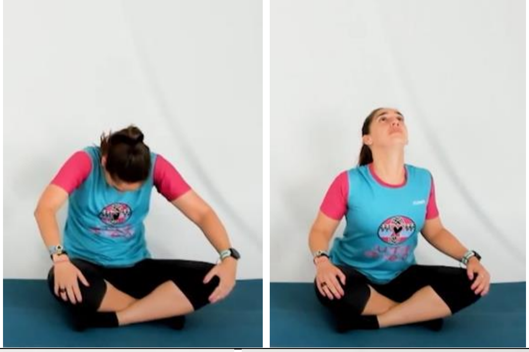 |
| Spinal twisting with one leg extended | 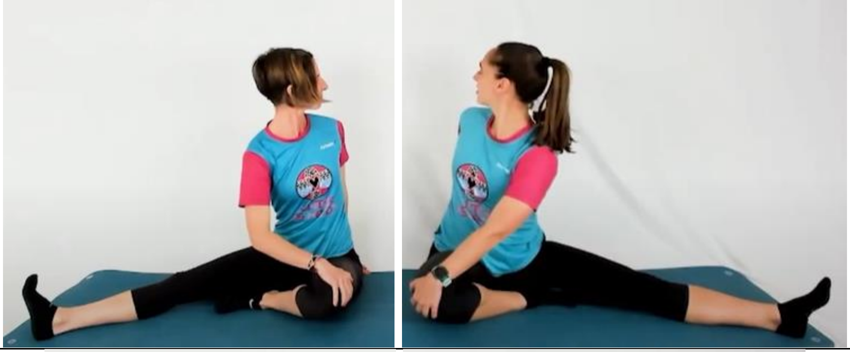 |
| Lateral tilt with one leg extended | 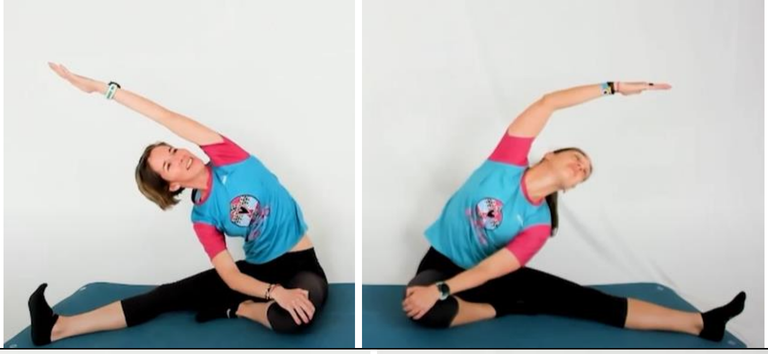 |
| Hip flexion with one leg extended | 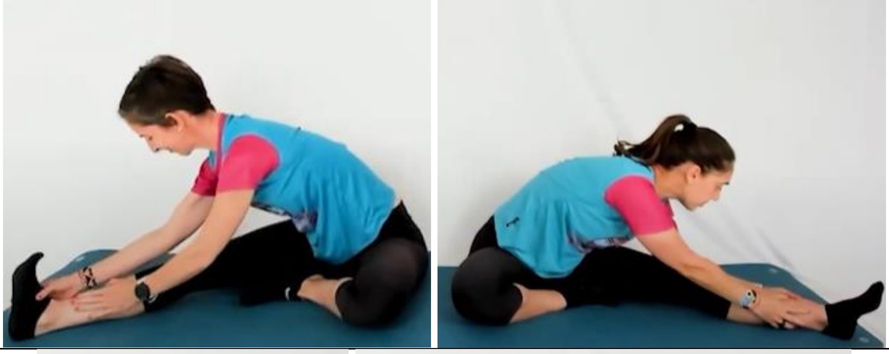 |
| Hip flexion with both legs extended | 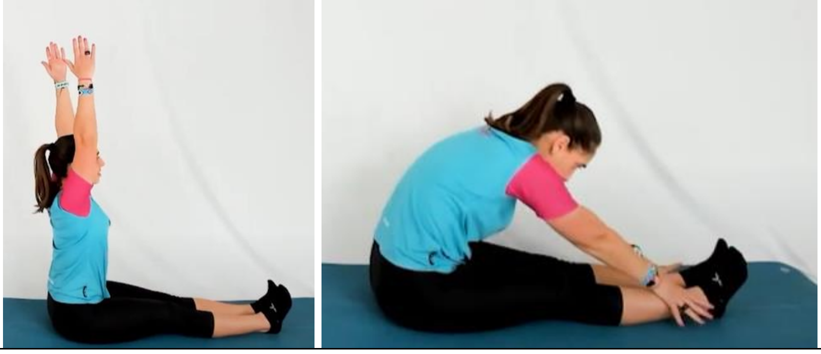 |

# Main part (4 rounds of 4 exercises with 1’ rest in between rounds) (20’’ Work – 10’’ Rest)

*Week 1 - Session 1 (Link)*

| Exercise | Photo |
|---|---|
| Scissor + jump | 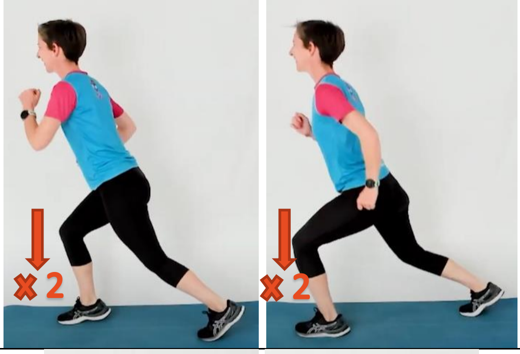 |
| Sumo squat | 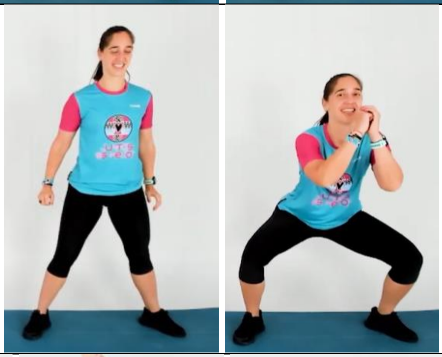 |
| Military press crossing arms above the head | 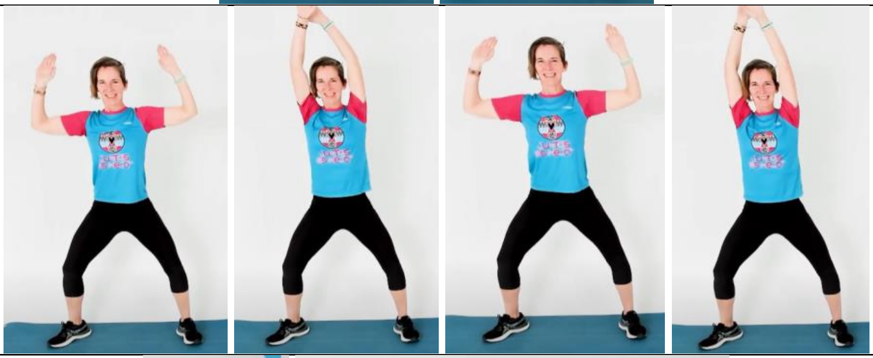 |
| Walking with your hands + plank | 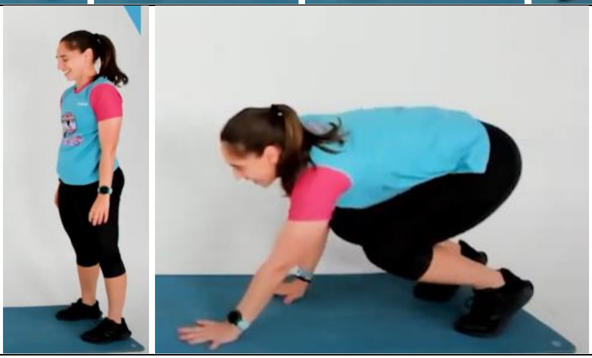 |

| | |
|---|---|
| | 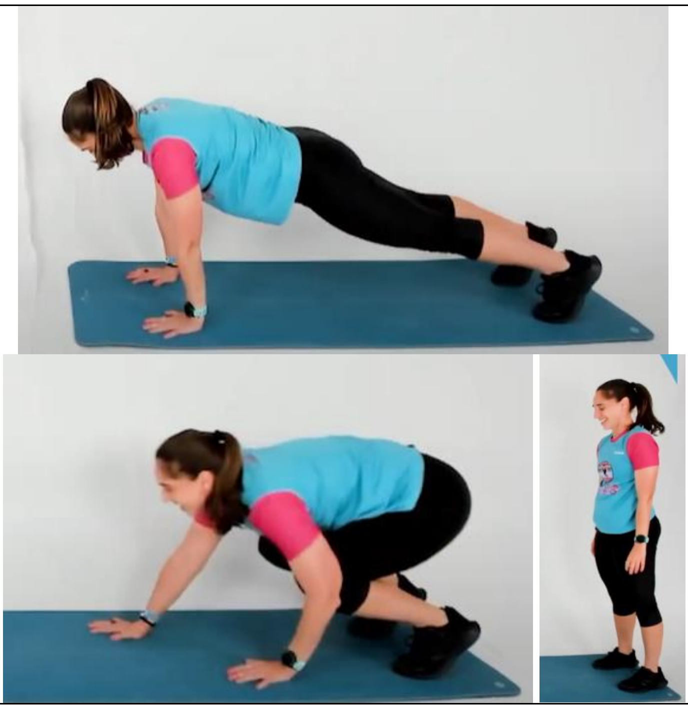 |

## *Week 1 - Session 2 (Link)*

| Exercise | Photo |
|---|---|
| Scissor + lateral arm lift | 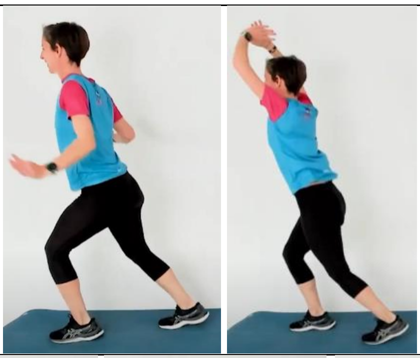 |
| Sumo squat + jump | 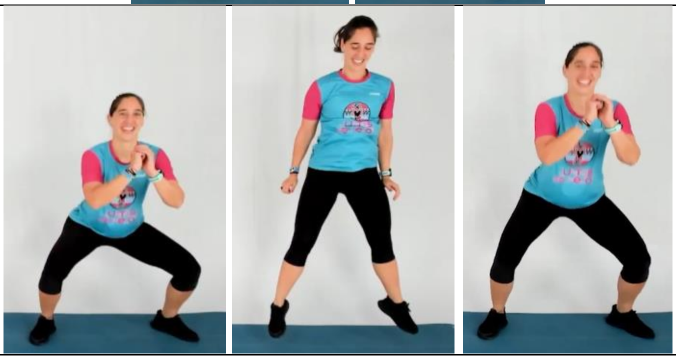 |

| | |
|---|---|
| Press militar + Double arms crossing | 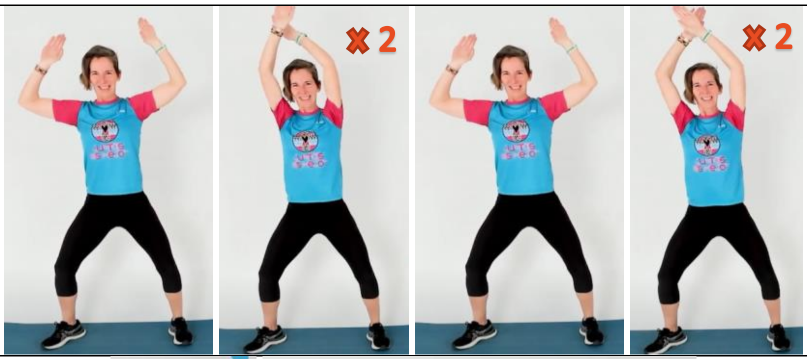 |
| Walking with your hands + plank + mountain climber | 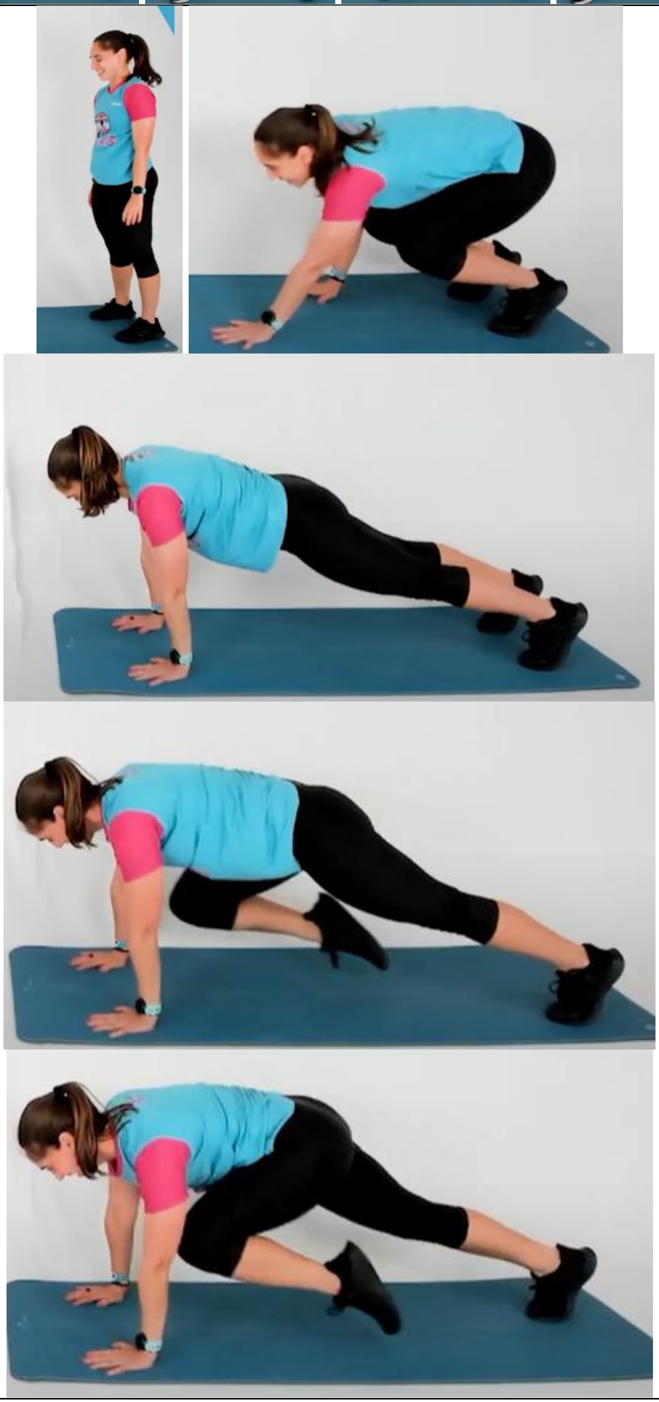 |

| | |
|---|---|
| | 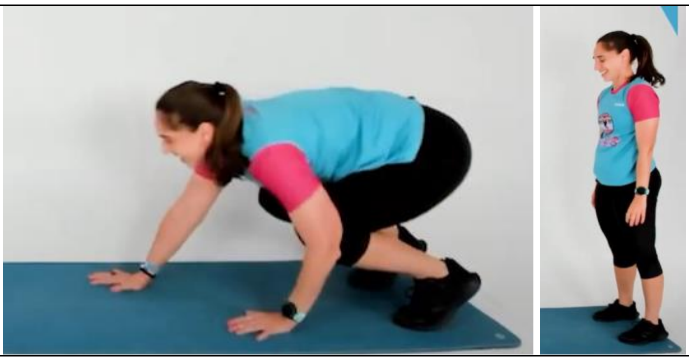 |

*Week 2 - Session 1 (Link)*

| Exercise | Photo |
|---|---|
| Skater jump | 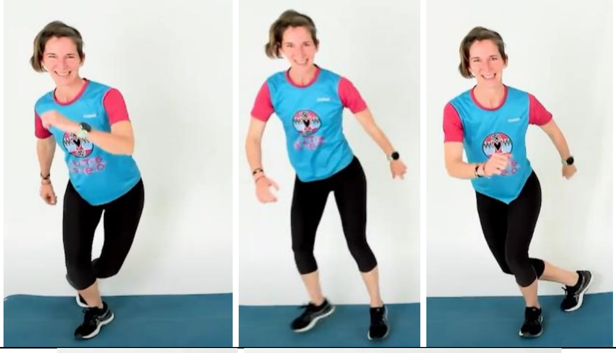 |
| Right stride behind | 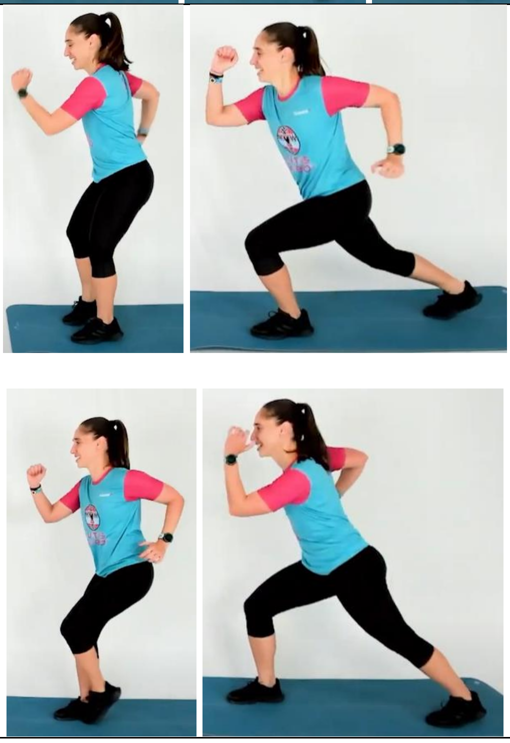 |

| | |
|---|---|
| Alternate tractions | 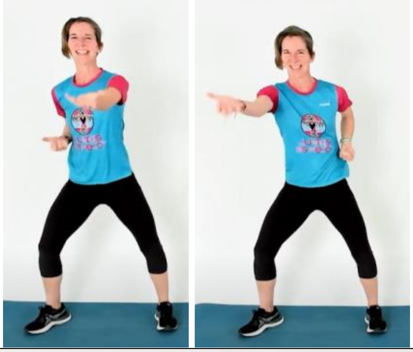 |
| Plank tap with diagonal hand | 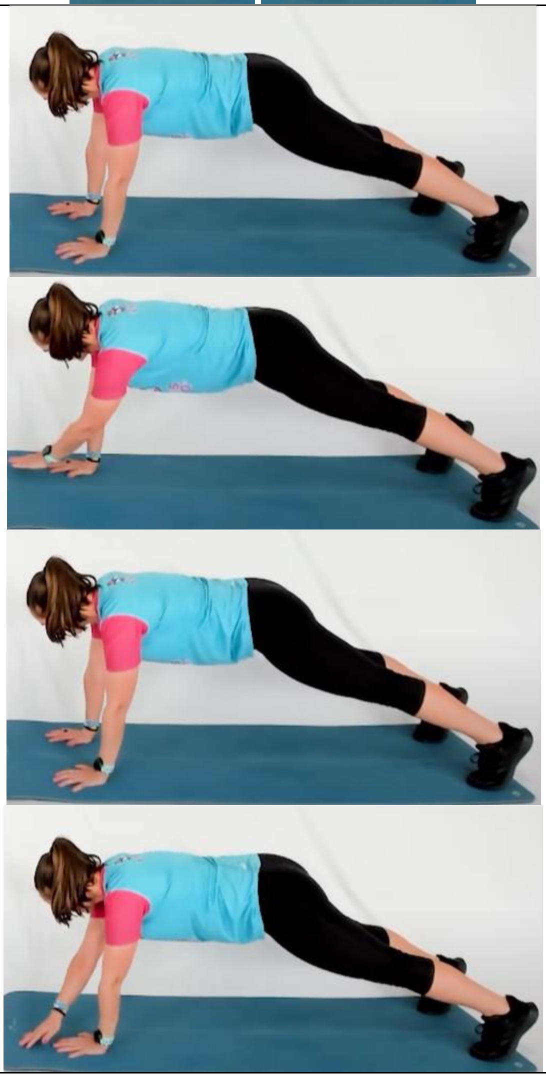 |

## *Week 2 - Session 2 (Link)*

| Exercise | Photo |
|---|---|
| Skater jump with hand down | 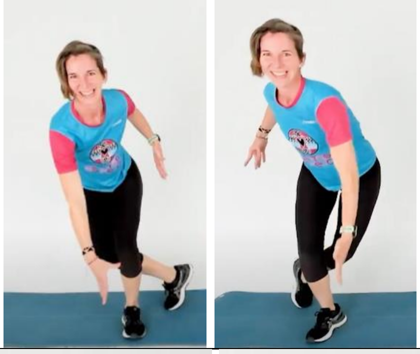 |
| From stride, kick in front | 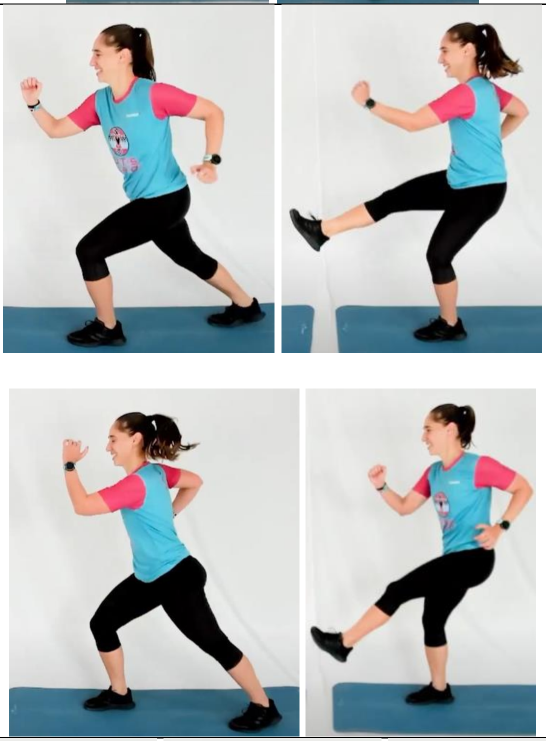 |
| Double tracctions | 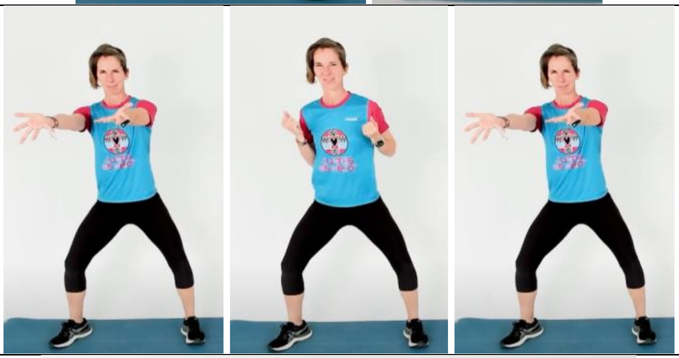 |
| Elbow plank tap, diagonal hand tap | 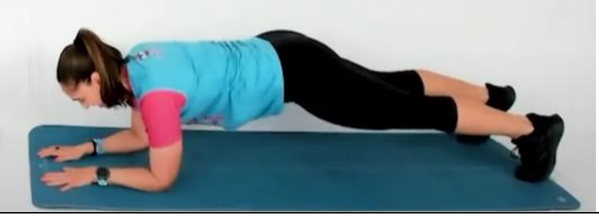 |

| | |
|---|---|
| | 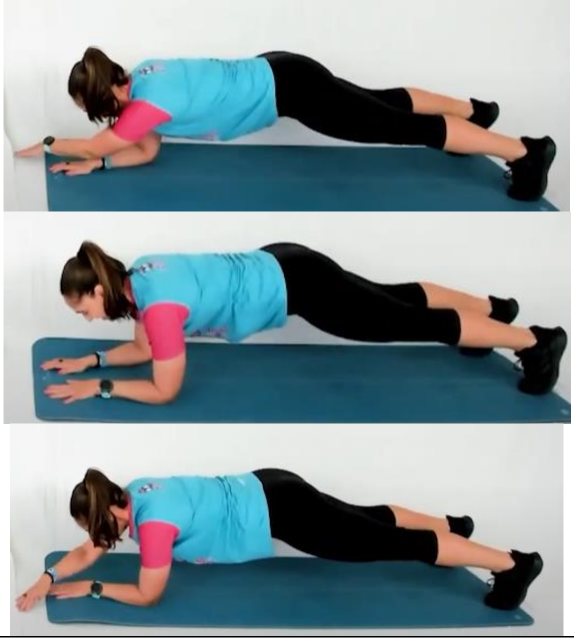 |

## *Week 3 - Session 1 (Link)*

| Exercise | Photo |
|---|---|
| Skipping | 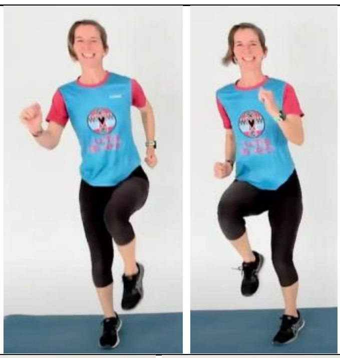 |
| Lunge jump | 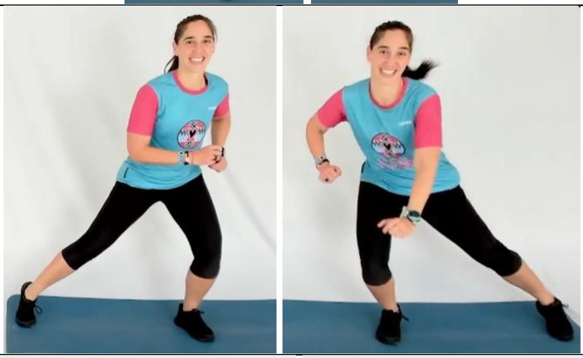 |
| Small semicircle of arms with extended elbows | 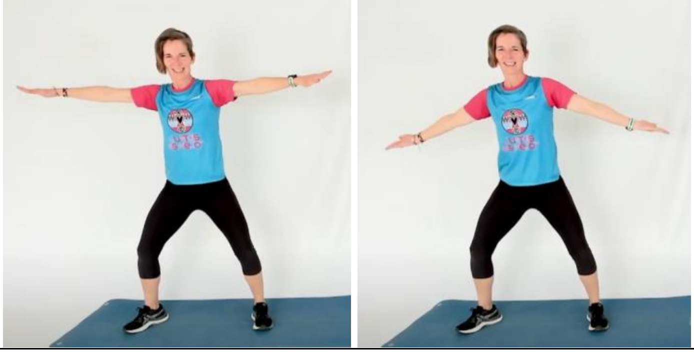 |

| | |
|---|---|
| From four-leg position, hand touches opposite knee | 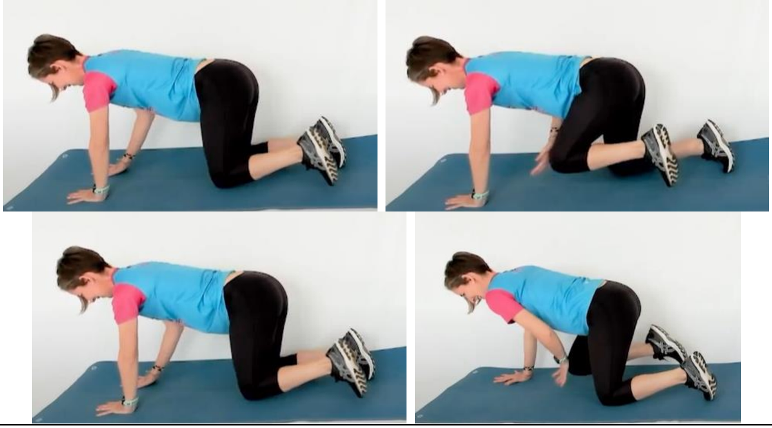 |

## *Week 3 - Session 2 (Link)*

| Exercise | Photo |
|---|---|
| Skipping + Rope movement with the arms | 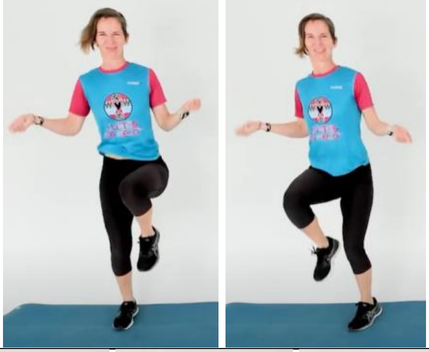 |
| Lunge jump with hand sliding down to the opposite side | 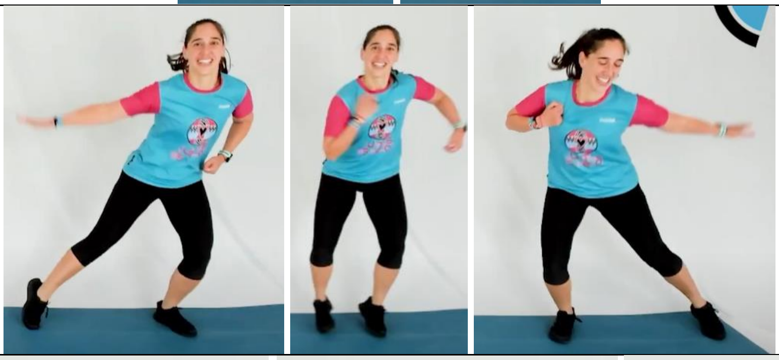 |
| Large semicircle of arms with extended elbows | 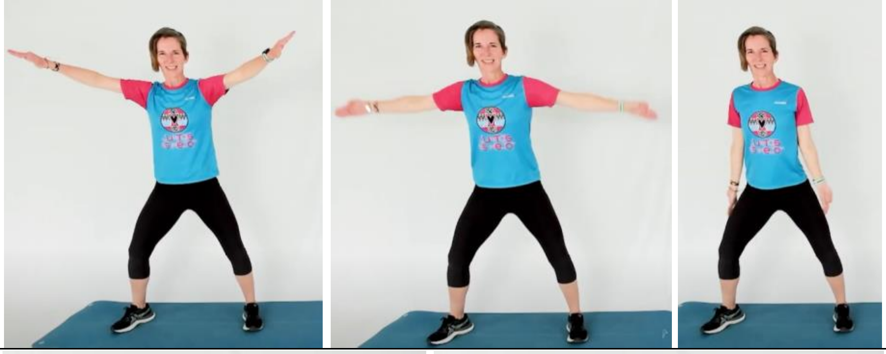 |
| From four-leg position with leg extended behind, hand touches opposite knee | 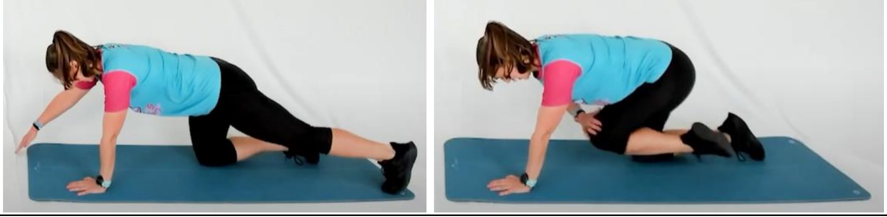 |

| | |
|---|---|
| | 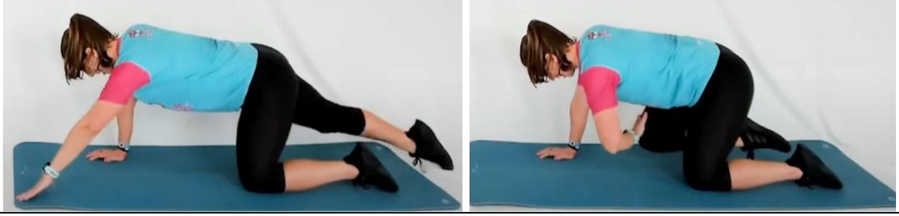 |

## *Week 4 - Session 1 (Link)*

| Exercise | Photo |
|---|---|
| Jumping hands on waist | 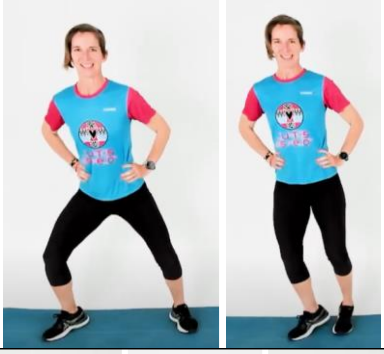 |
| Tiptoe walking | 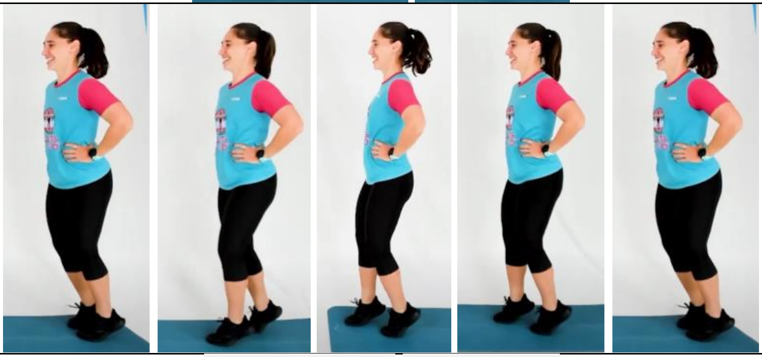 |
| Pumping of arms extended back | 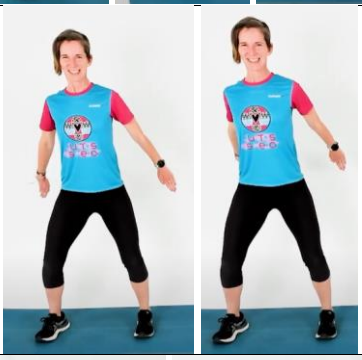 |
| Knee elevation from four-footed position | 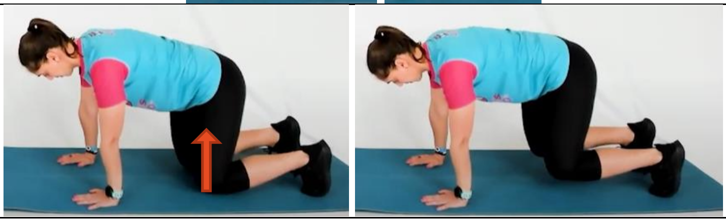 |

## *Week 4 - Session 2 (Link)*

| Exercise | Photo |
|---|---|
| Jumping fists up | 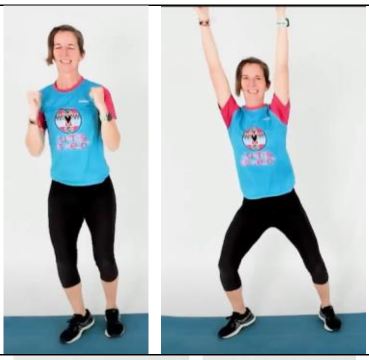 |
| Flexo-extension of ankles | 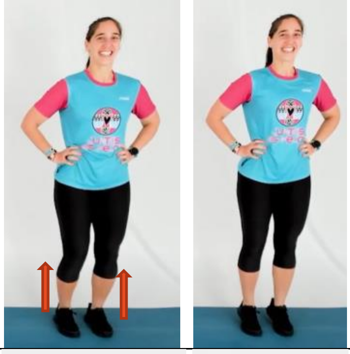 |
| 4X pumping of arms extended back + cross arms in front | 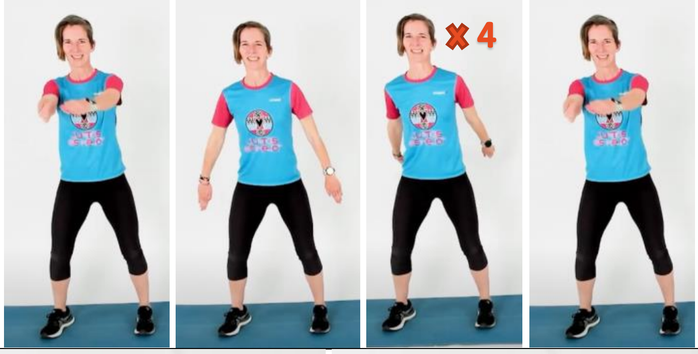  |
| Knee elevation from four-footed position and semi-extension back | 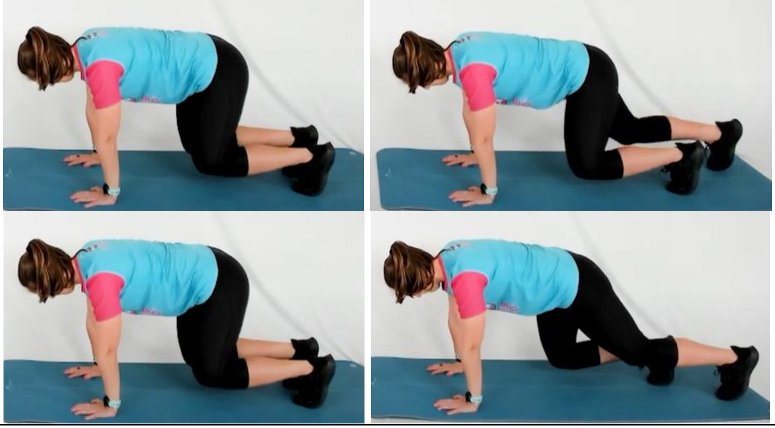 |

# Month 4 (40" of work - 15" of recovery)

## Common Part

*Dynamic Warm-up (At the beginning of each session before the main part) (Link)*

| Exercise | Photograph |
| --- | --- |
| Semicircle rotation of the neck | 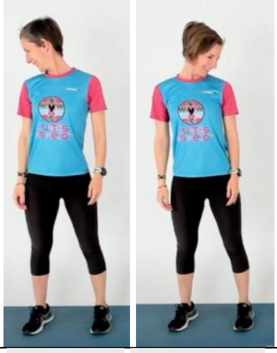 |
| Shoulder lift | 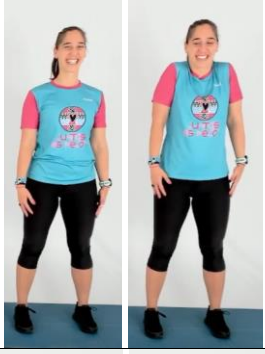 |
| Extension and flexion of fingers | 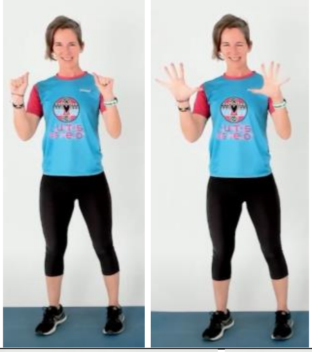 |
| Cross arms and elbow flexo-extension | 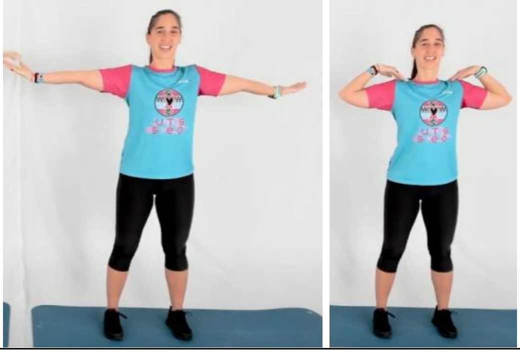 |

| | |
|---|---|
| Shoulder rotation with hands on shoulders | 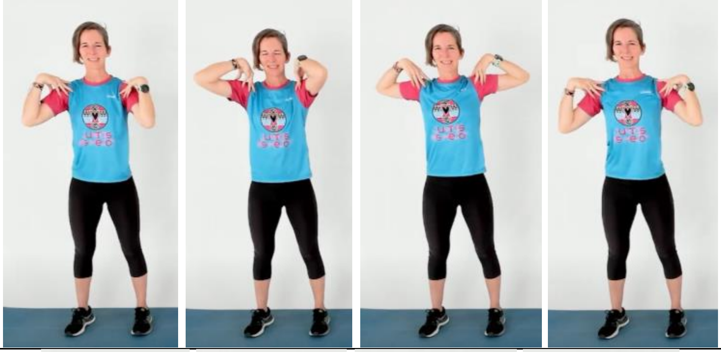 |
| Leg swinging, knee flexes in front and extends behind | 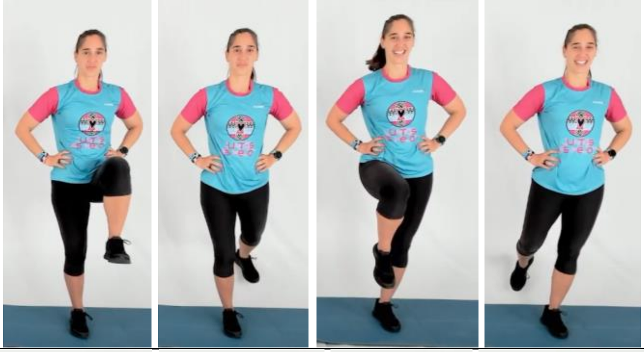 |
| In balance flexo-extension of the knee | 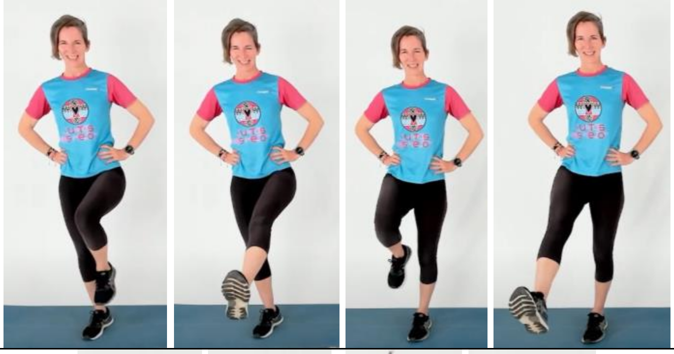 |
| 3X jump side to side | 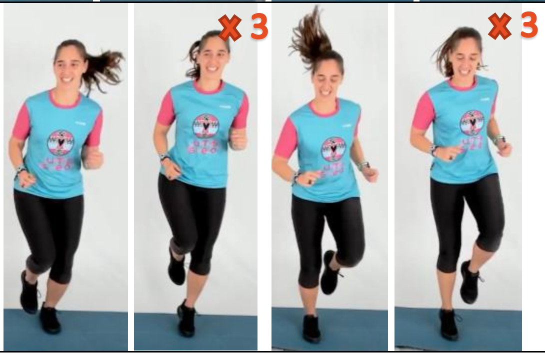 |

## *Stretching (At the end of each session after the main part) (Link)*

| Stretch | Photograph |
| --- | --- |
| Neck tilt with hand aid | 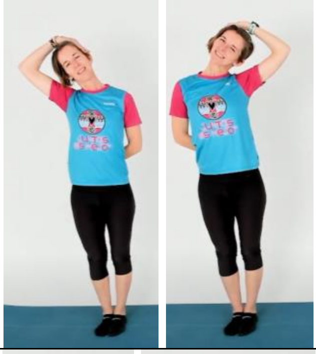 |
| Hands behind the neck and cat camel | 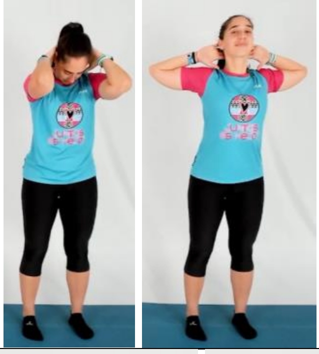 |
| Open legs, lateral tilt | 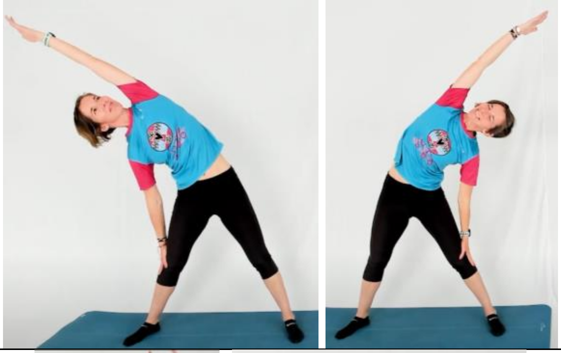 |
| One leg in front of another. Hip flexion | 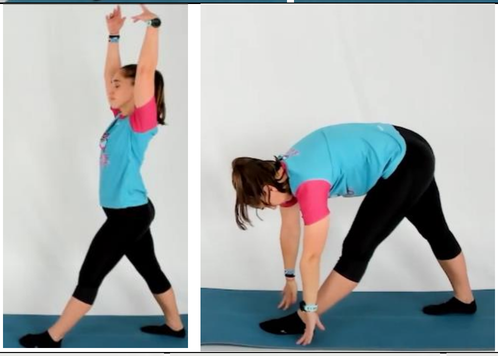 |
| Low lunge | 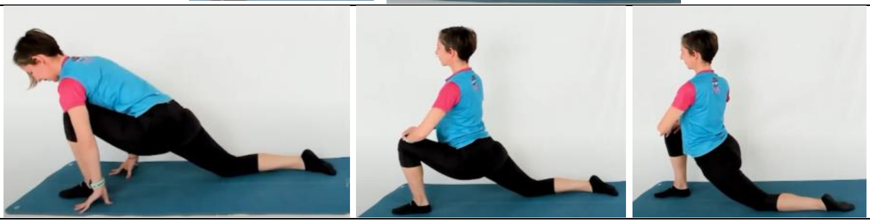 |

| | |
|---|---|
| Low lunge + twist | 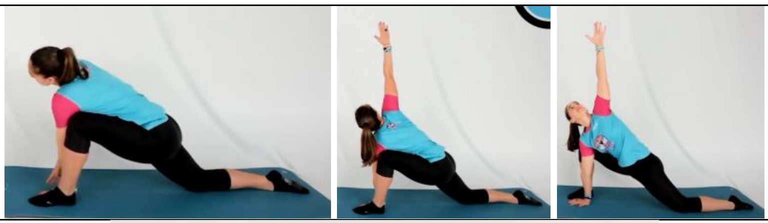 |
| From sitting position, legs extended and abduction with hip flexion | 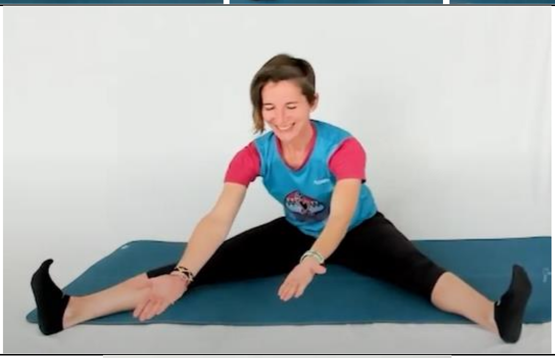 |
| Bound angle pose | 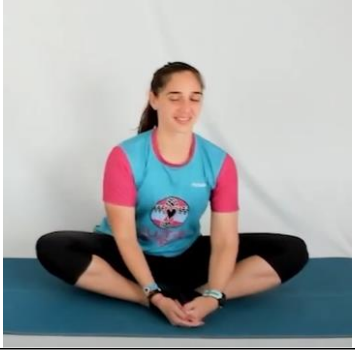 |

## Main part (4 rounds of 4 exercises with 1’ rest in between rounds) (40’’ Work – 15’’ Rest)

### *Week 1 - Session 1 (Link)*

| Exercise | Photo |
|---|---|
| Jump front-back | 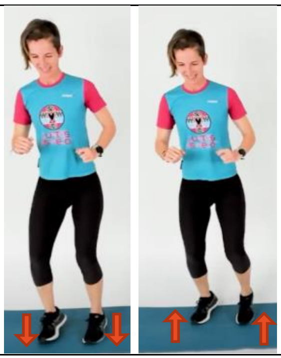 |

<table>
<tr><td>Sumo squat with alternating heel lift</td><td>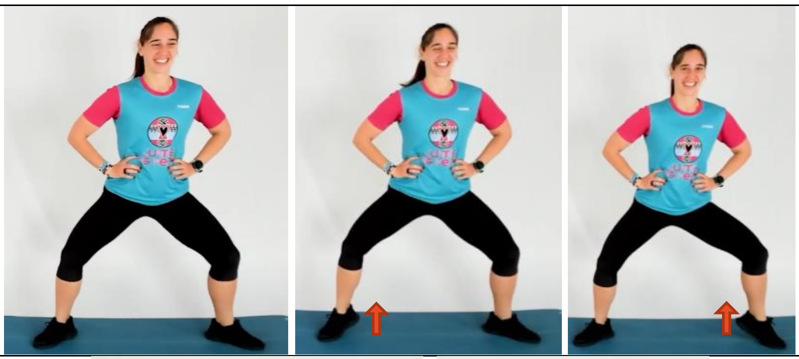</td></tr>
<tr><td>From arm cross position abduction-adduction</td><td>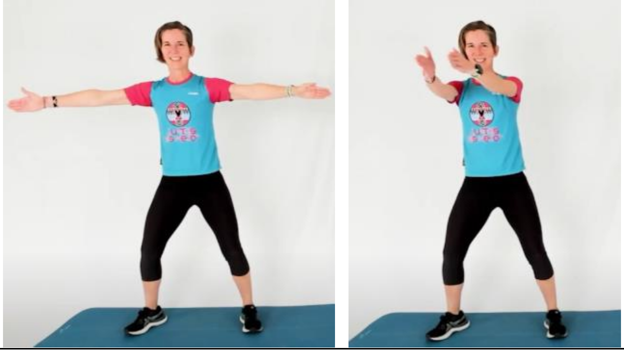</td></tr>
<tr><td rowspan="4">Plank touch opposite elbow</td><td>Level 1</td></tr>
<tr><td>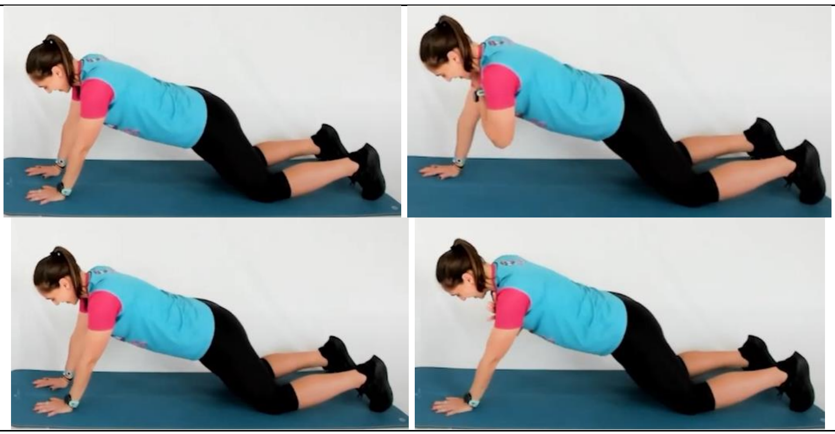</td></tr>
<tr><td>Level 2</td></tr>
<tr><td>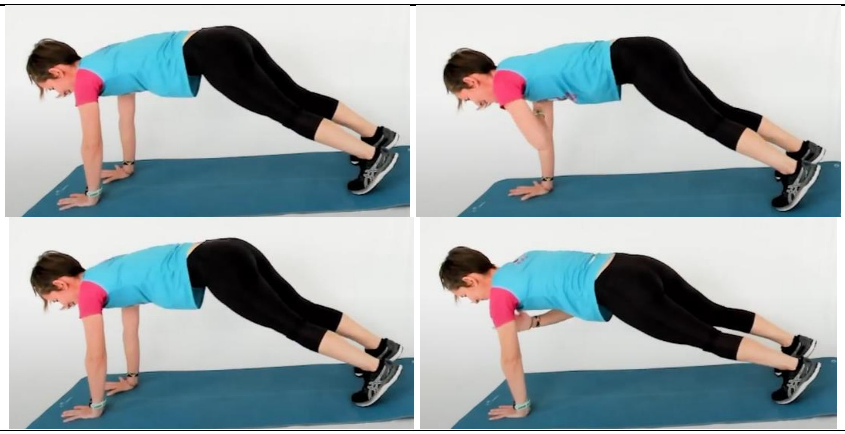</td></tr>
</table>

## Week 1 - Session 2 (Link)

| Exercise | Photo |
|---|---|
| Sprint in front-sprint behind | 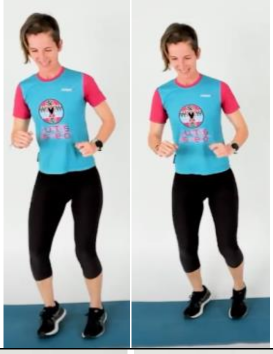 |
| Sumo squat with heel lift | 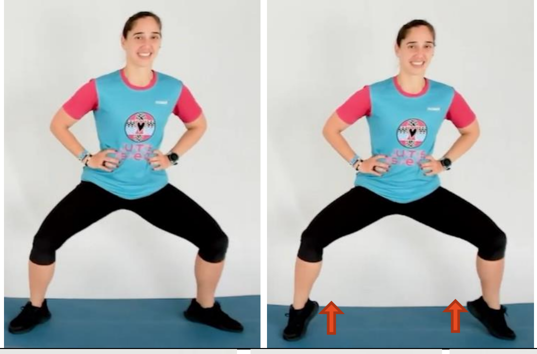 |
| Arms cross + Abduction-adduction crossing in front | 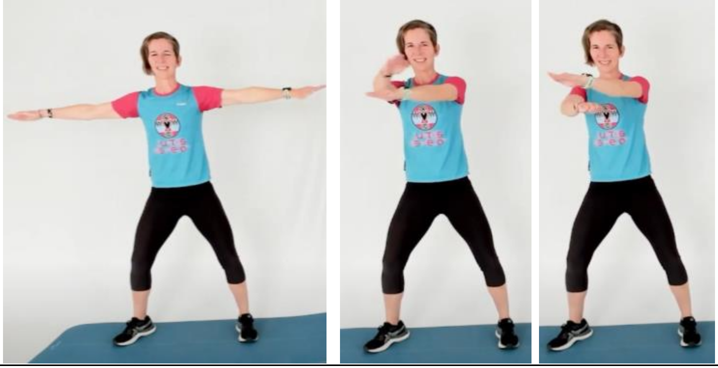 |
| Plank touch opposite hip | **Level 1** |
| | 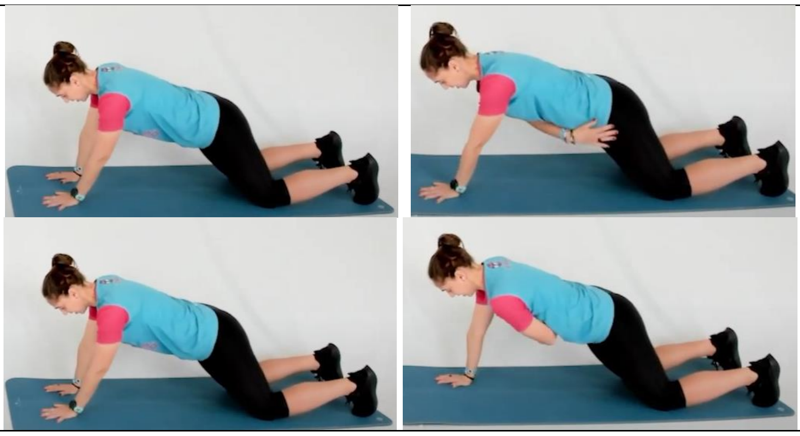 |
| | **Level 2** |
| | 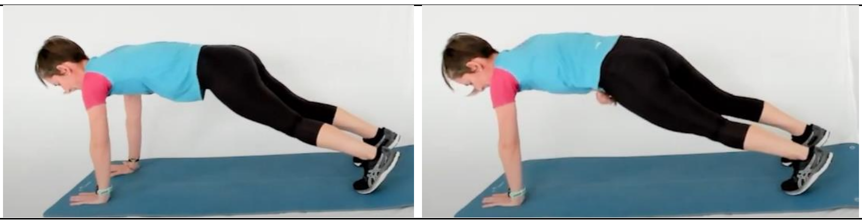 |

| | |
|---|---|
| | 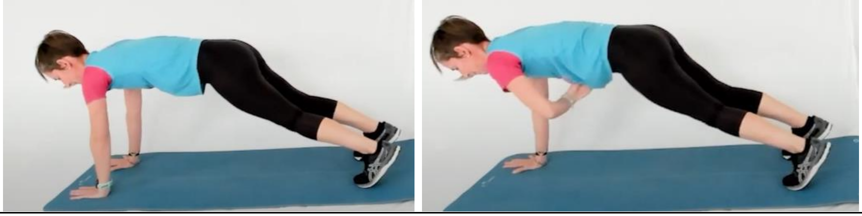 |

*Week 2 - Session 1 (Link)*

| Exercise | Photo |
|---|---|
| Cross jumping | **Level 1** 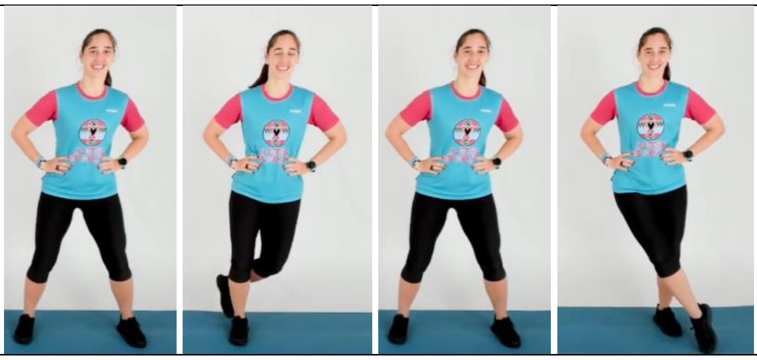 **Level 2** 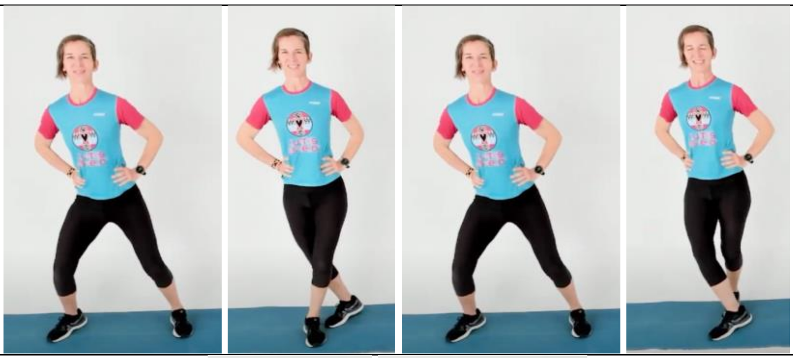 |
| Hip rotation with leg lift | 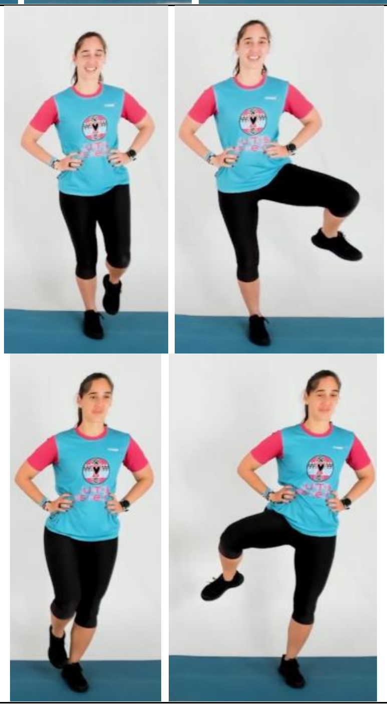 |

| | |
|---|---|
| Four-footed and tap in front of alternate hands | 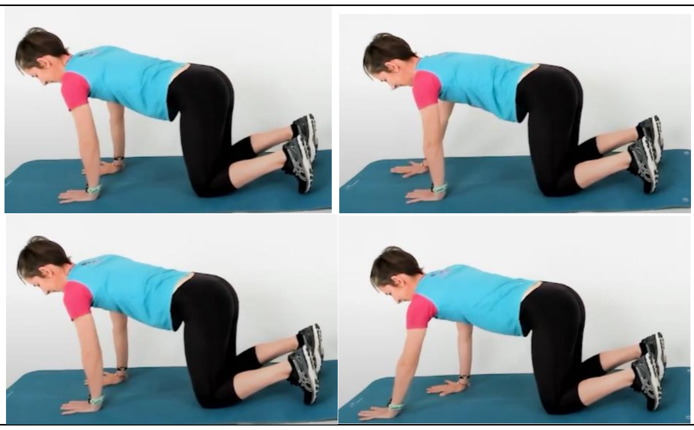 |
| Elbow plank and ankle flexo-extension | **Level 1**<br>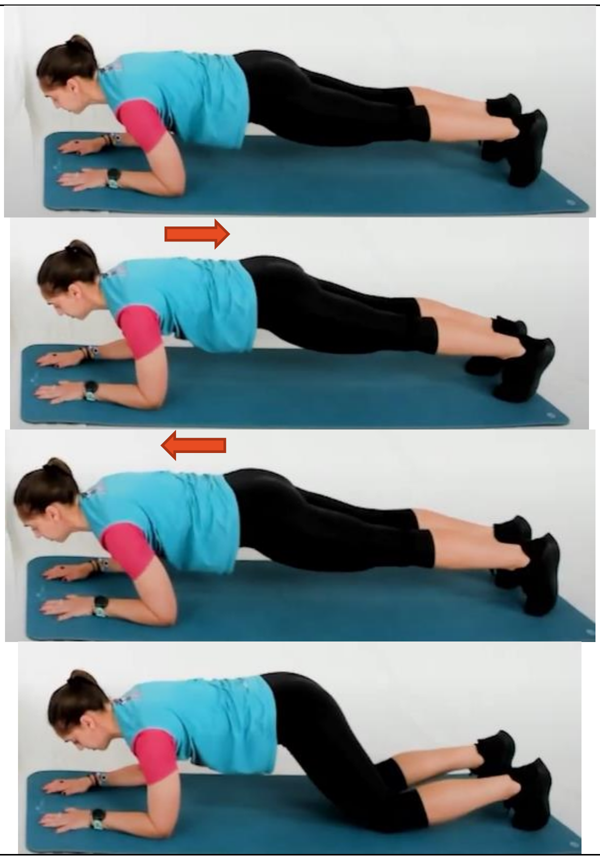<br>**Level 2**<br>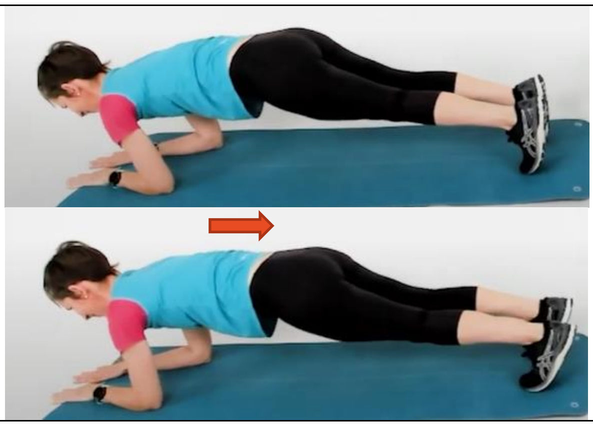 |

| | |
|---|---|
| | 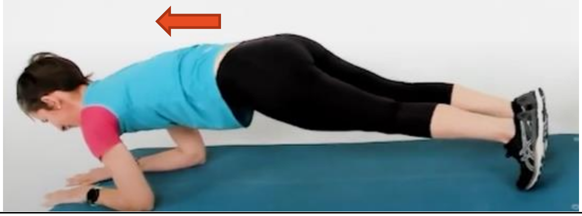 |

## *Week 2 - Session 2 (Link)*

| Exercise | Photo |
|---|---|
| | **Level 1** |
| | 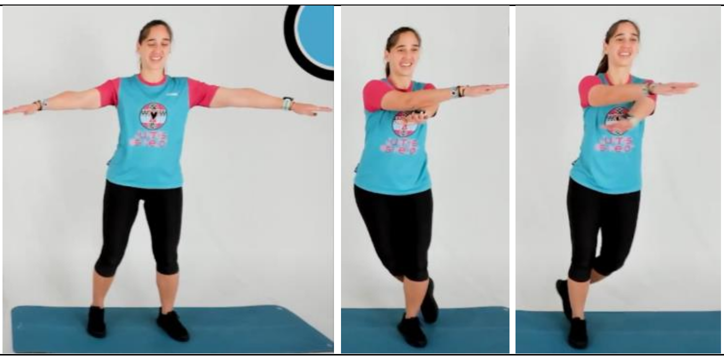 |
| Cross jumping + cross arms | **Level 2** |
| | 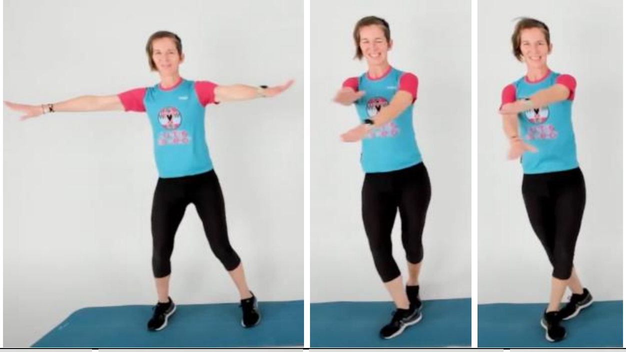 |
| Abduction with skater | 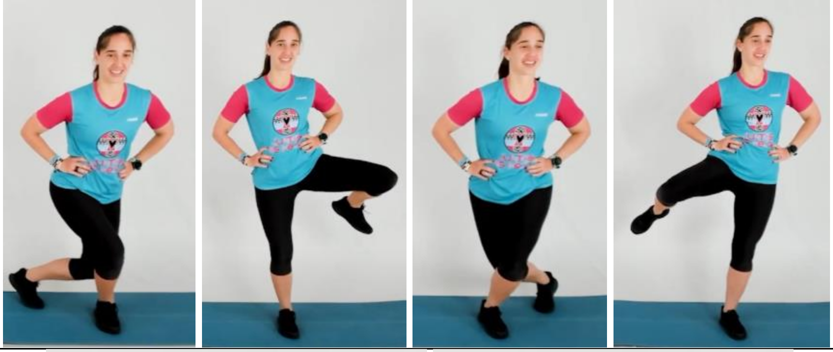 |
| Four-footed position and tap in front of hands | 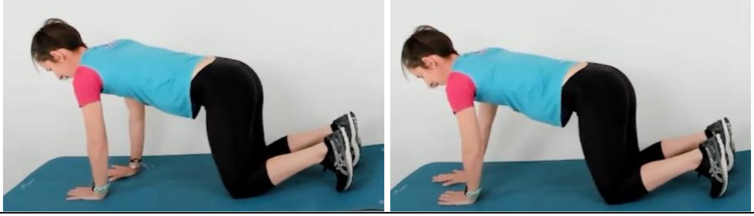 |

| | |
|---|---|
| | 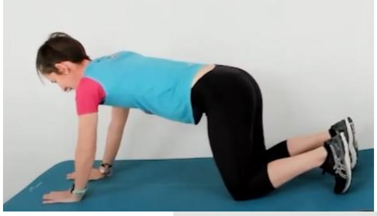 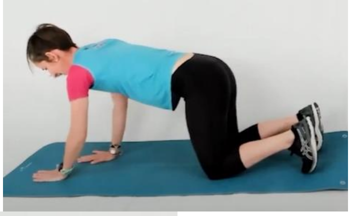 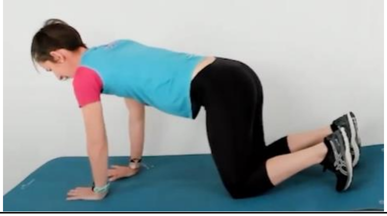 |
| Elbow plank with hip rotation | **Level 1** |
| | 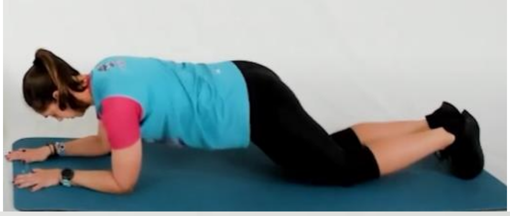 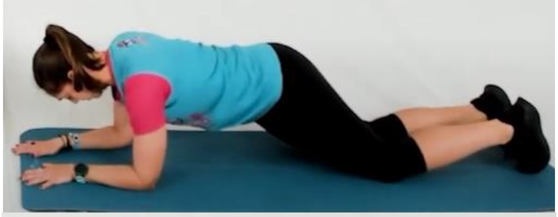 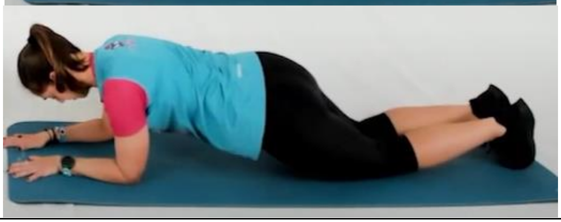 |
| | **Level 2** |
| | 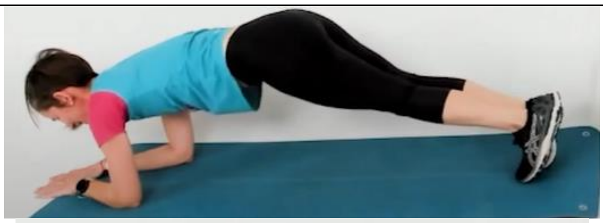 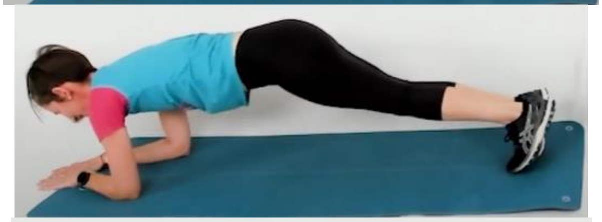 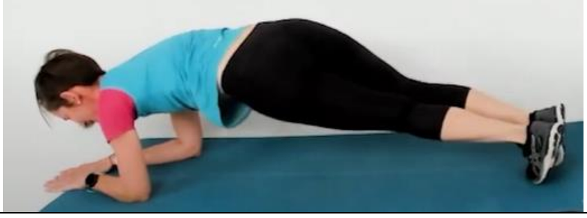 |

*Week 3 - Session 1 (Link)*

| Exercise | Photo |
|---|---|
| Sky side to side | 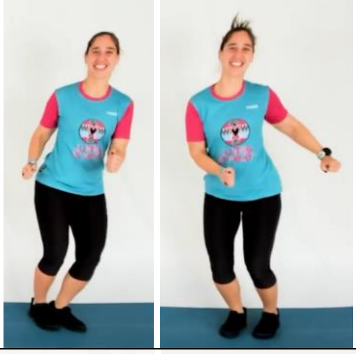 |
| Sumo squat + leg abduction | 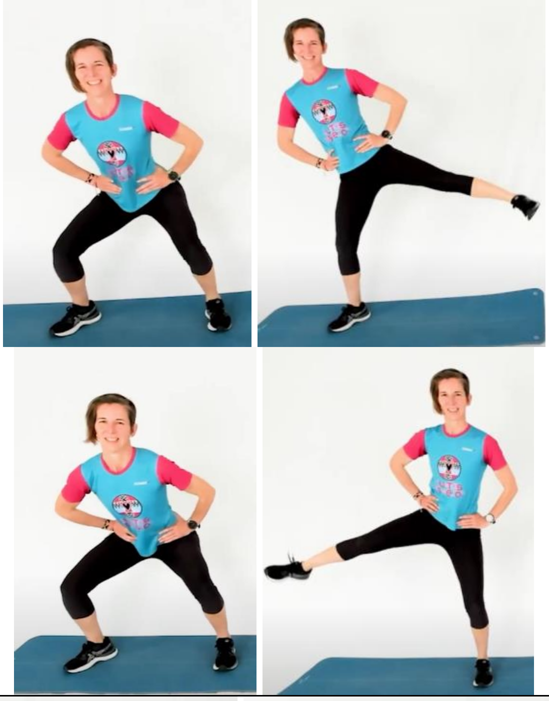 |
| Four-footed position + bending an elbow | 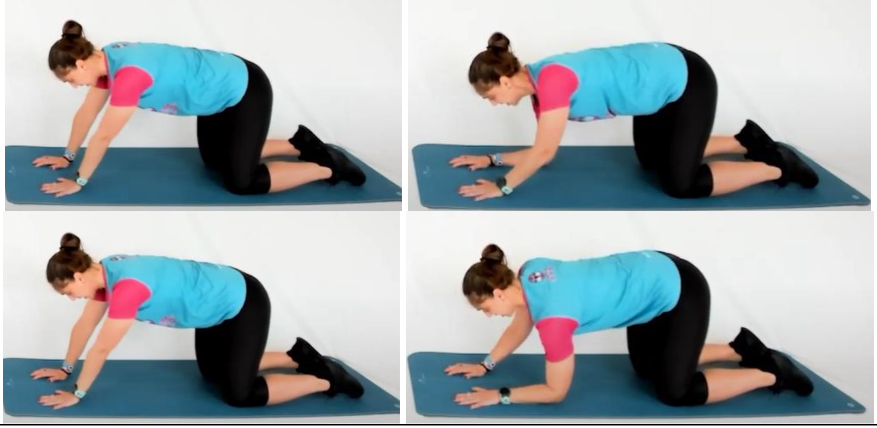 |
| | **Level 1** |
| Mountain climber | 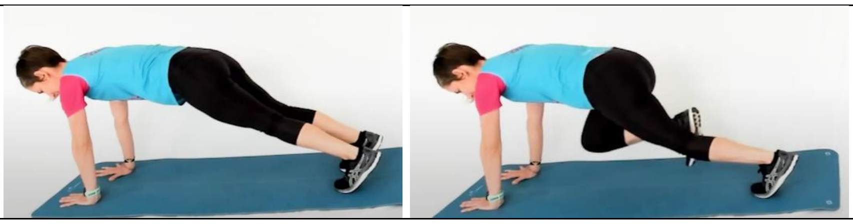 |

| | |
|---|---|
| | 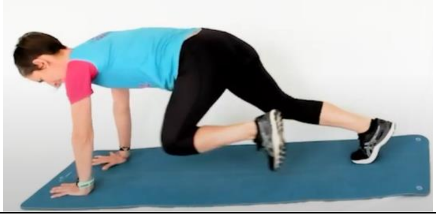 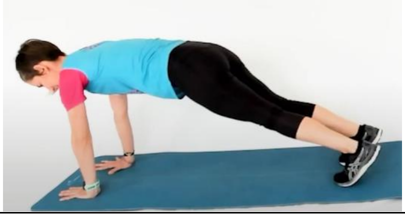 |
| | **Level 2** |
| | 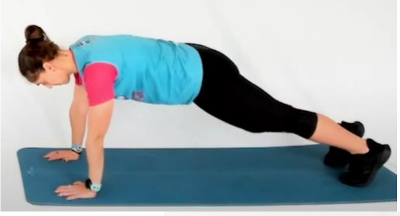 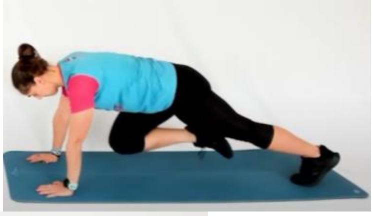 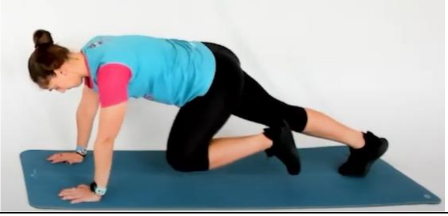 |

## Week 3 - Session 2 (Link)

| Exercise | Photo |
|---|---|
| Sky side to side x2 | 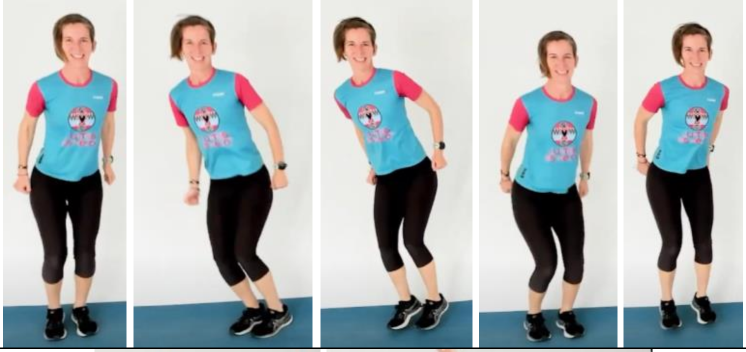 |
| Sumo squat + leg abduction + extended arm lift | 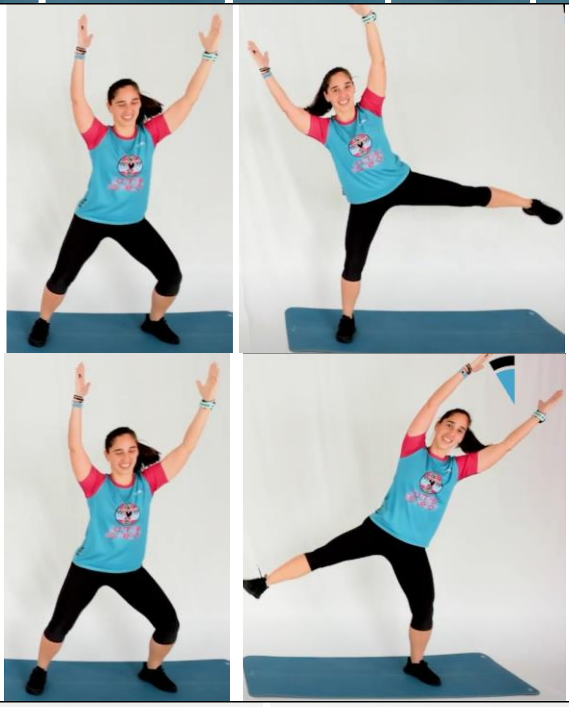 |
| Four footed leg + bending two elbows | 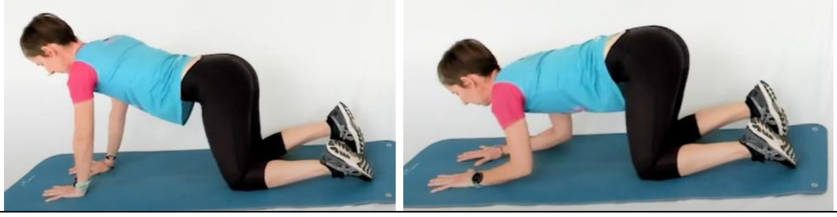 |
| Cross body mountain climber | **Level 1**<br>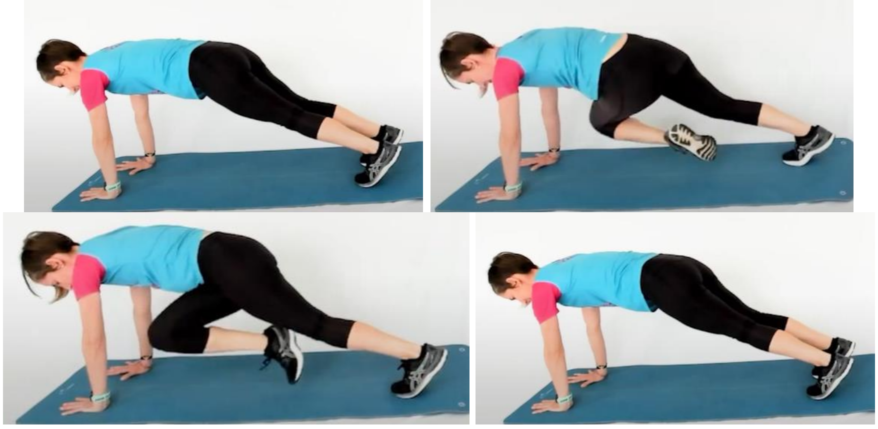 |

| | Level 2 |
|---|---|
| | 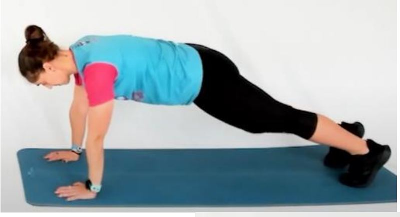 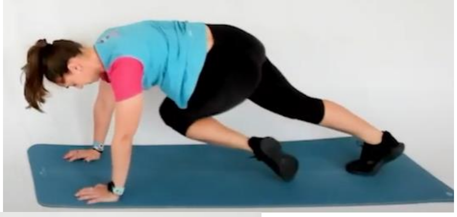 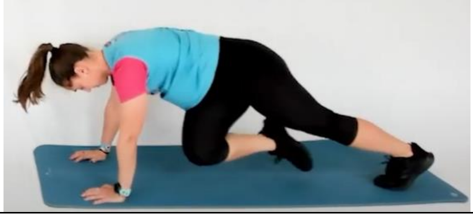 |

## *Week 4 - Session 1 (Link)*

| Exercise | Photo |
|---|---|
| Skipping | 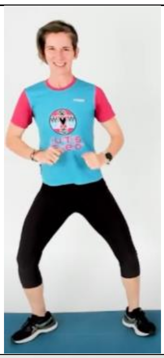 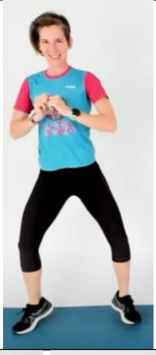 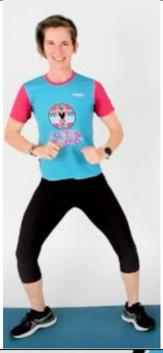 |
| Squat + glute kick | 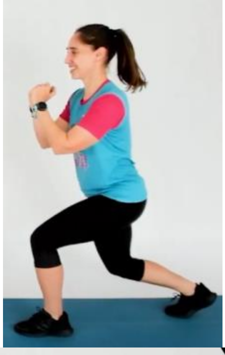 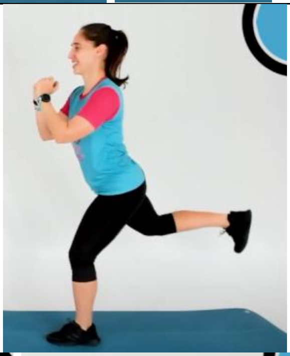 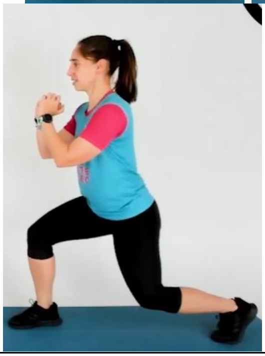 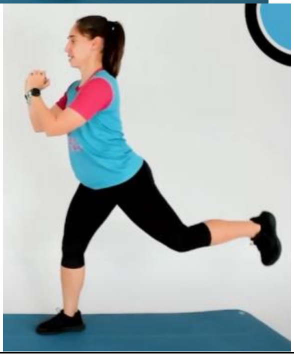 |

| | |
|---|---|
| Cuadrup touch opposite elbow | 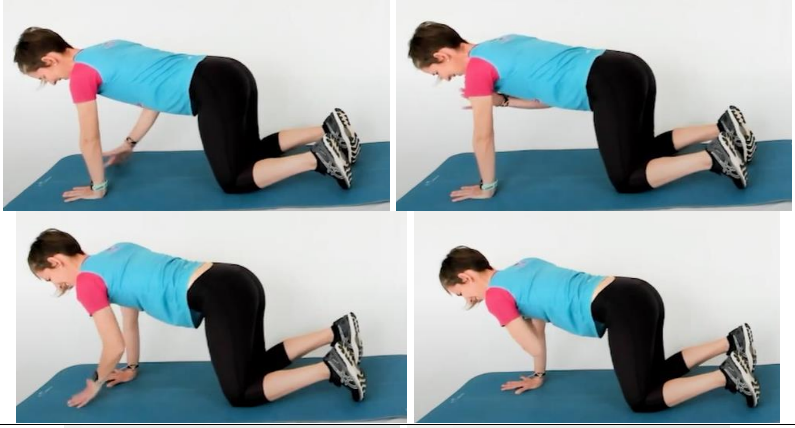 |
| Sit down + torsion side to side | 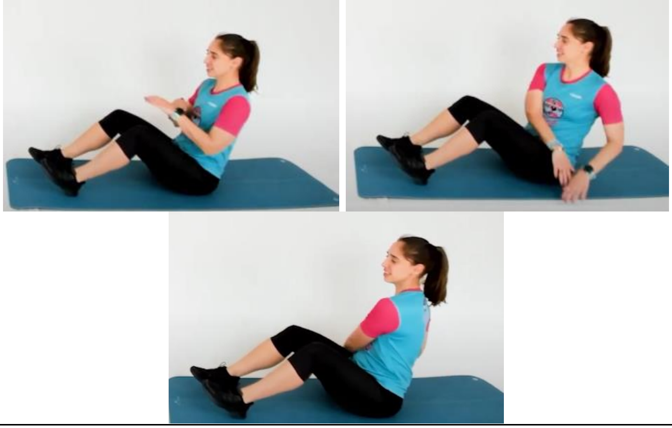 |

## *Week 4 - Session 2 (Link)*

| Exercise | Photo |
|---|---|
| Skipping | 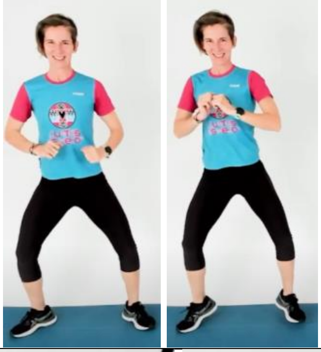 |
| Lunge + leg lift back + knee lift in front | 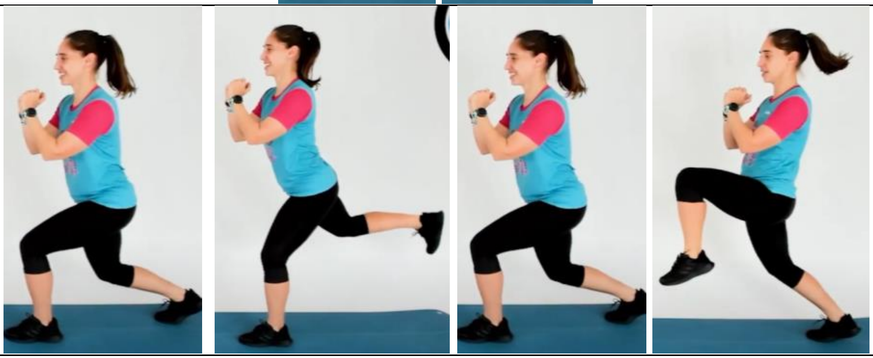 |

| | |
|---|---|
| | 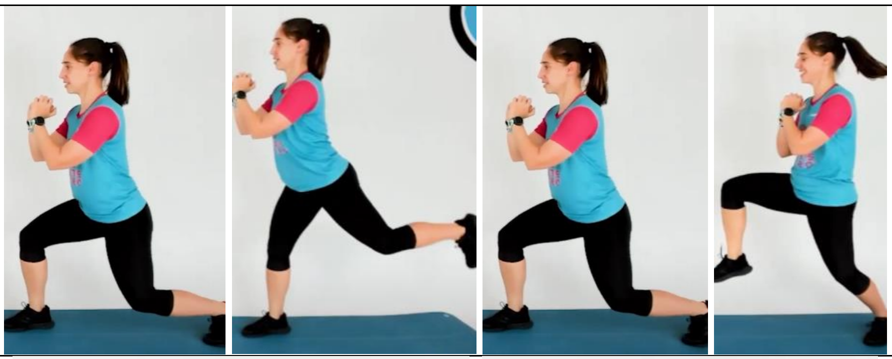 |
| From four-footed push-up + touch the opposite shoulder | 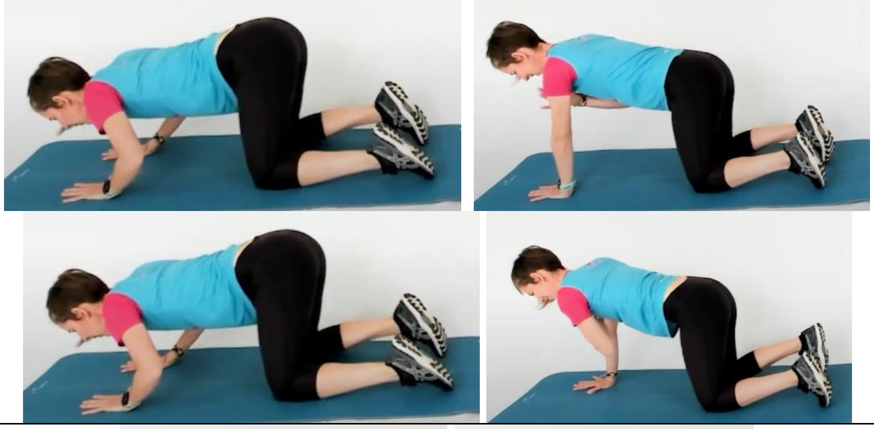 |
| Sit down: side to side twist with legs up | 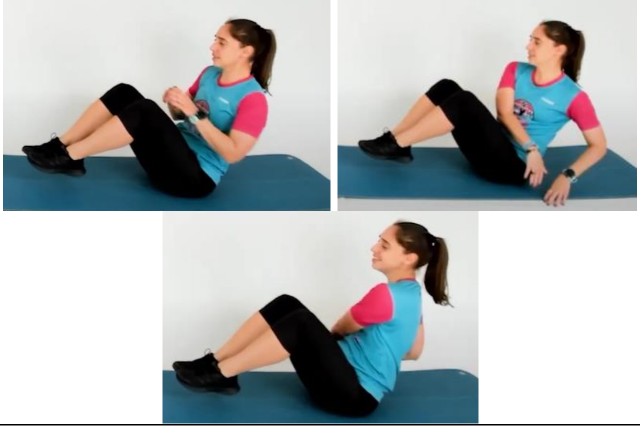 |

# Month 5 (40" of work - 15" of recovery)

## Common Part

*Dynamic Warm-up (At the beginning of each session before the main part) (Link)*

| Exercise | Photograph |
|---|---|
| Flex-extension + neck rotation | 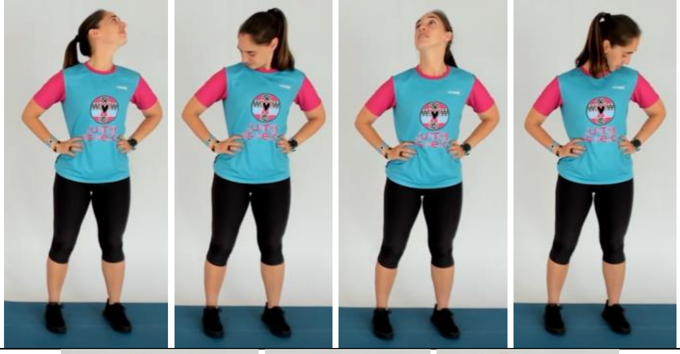 |
| With hands on shoulders, shoulder rotation | 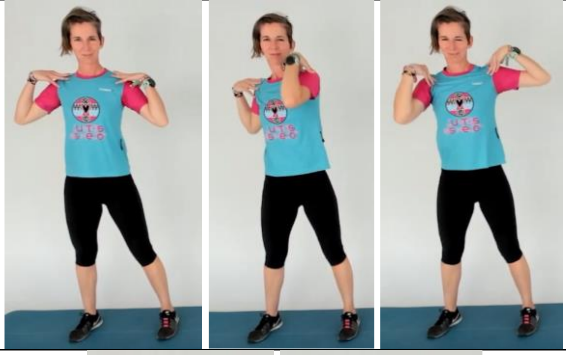 |
| Semisquat + column rotation side to side | 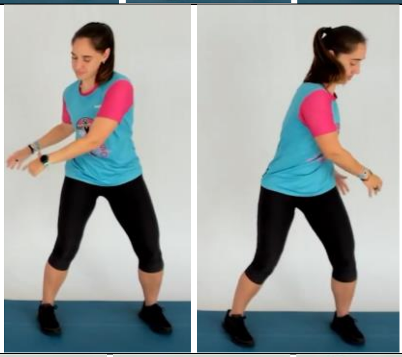 |
| Hip circunduction | 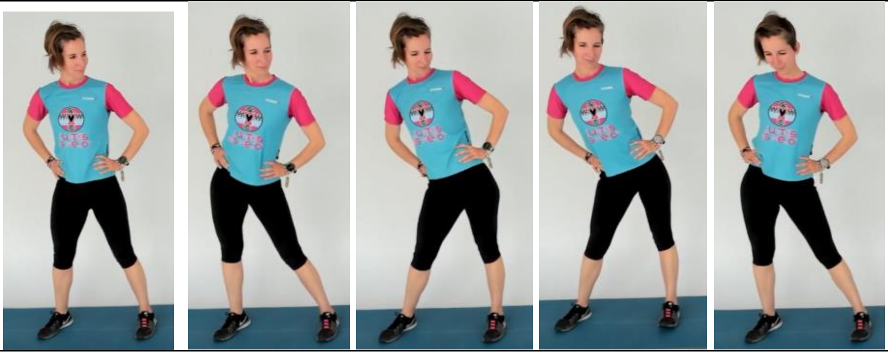 |

| | |
|---|---|
| Hip rotation | 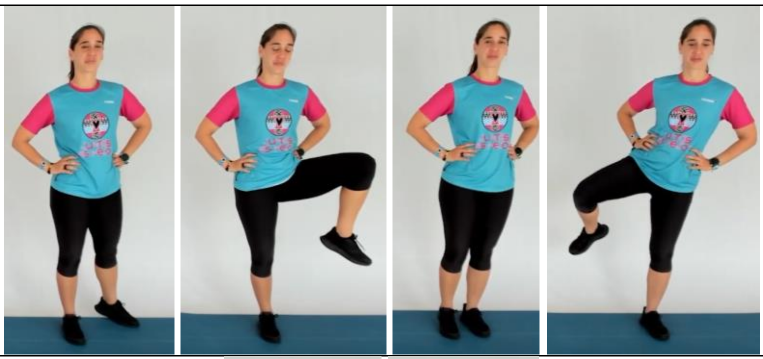 |
| March on site | 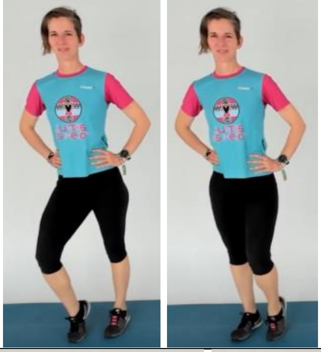 |
| Lateral movement + lateral kick | 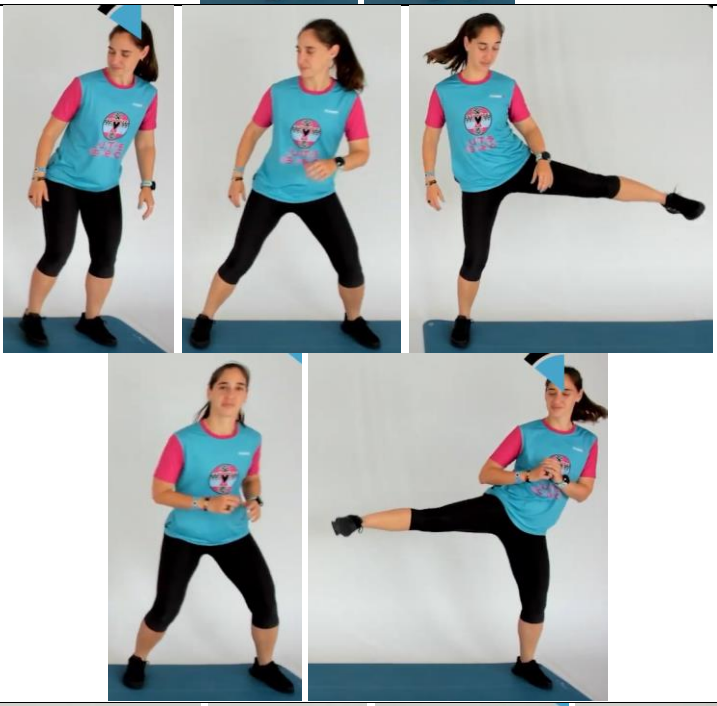 |
| Lateral movement + skater | 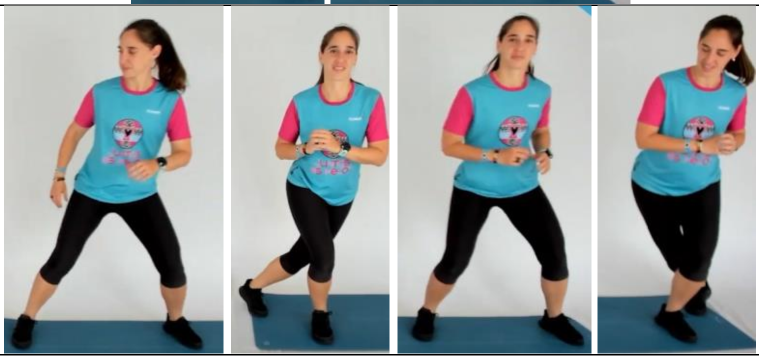 |

| | |
|---|---|
| Lateral movement + knee lift to touch opposite hand | 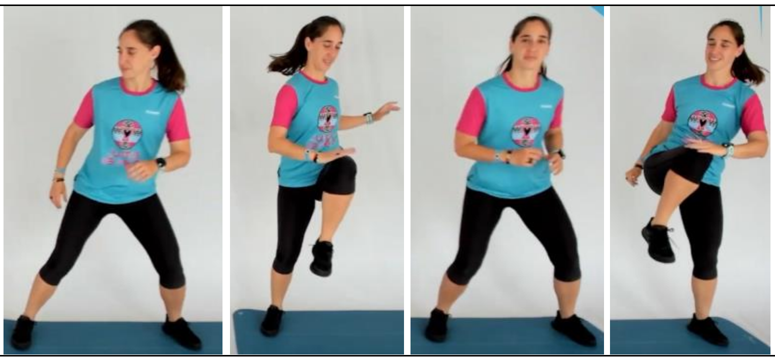 |

## *Stretching (At the end of each session after the main part) (Link)*

| Stretch | Photograph |
|---|---|
| Neck lateral assisted stretching | 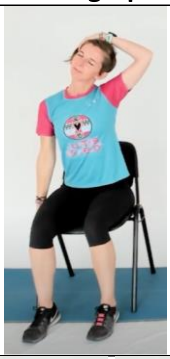 |
| Column torsion sitting in chair | 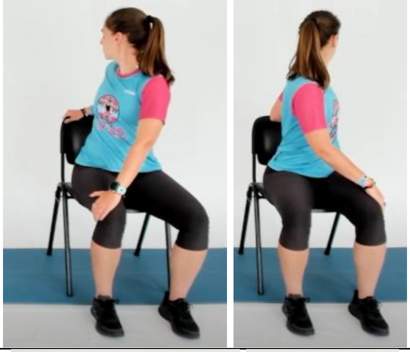 |
| Lateral inclination of the spine + arm extension up | 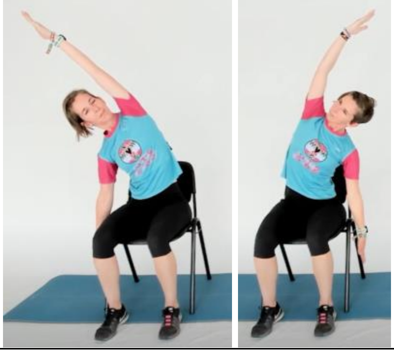 |

| | |
|---|---|
| Cat-camel sitting in a chair | 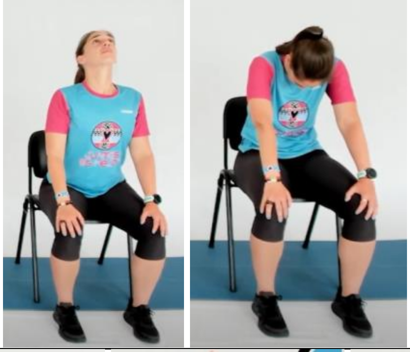 |
| Leg abduction + trunk flexion sitting in chair + twisting with arm lift | 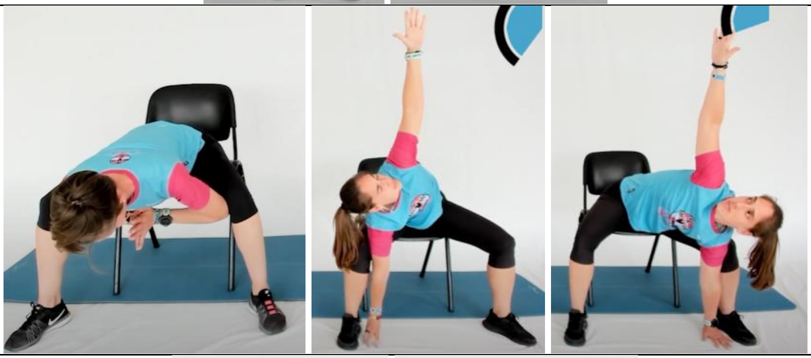 |
| Pyramidal stretch sitting in chair | 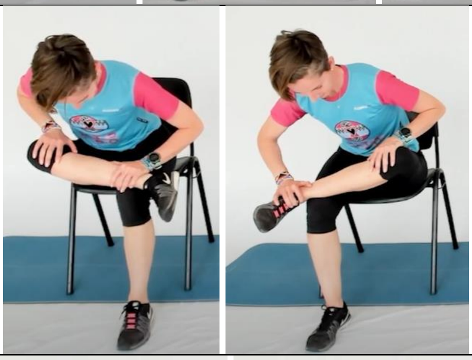 |
| Posterior chain stretch with trunk flexion front sitting in chair | 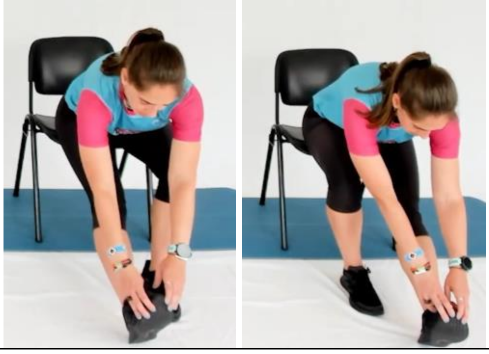 |

# Main part (4 rounds of 4 exercises with 1’ rest in between rounds) (40’’ Work – 15’’ Rest)

## *Week 1 - Session 1 (Link)*

| Exercise | Photo |
|---|---|
| Sprint + Sumo squat | 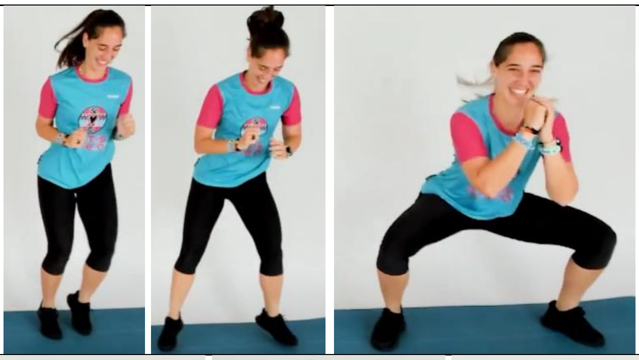 |
| Prisioner squat + lateral jump | 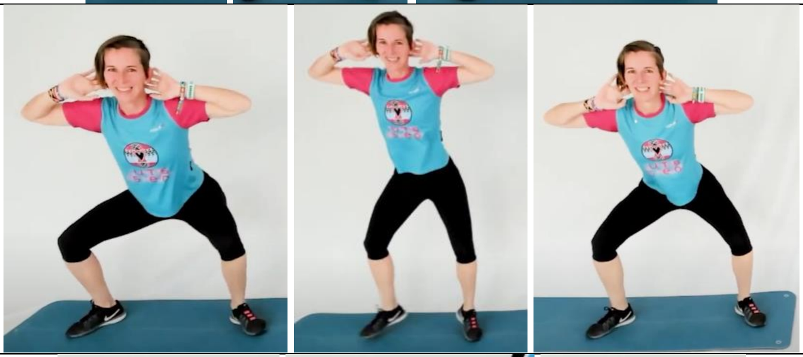 |
| Arm rotation with hands in front | 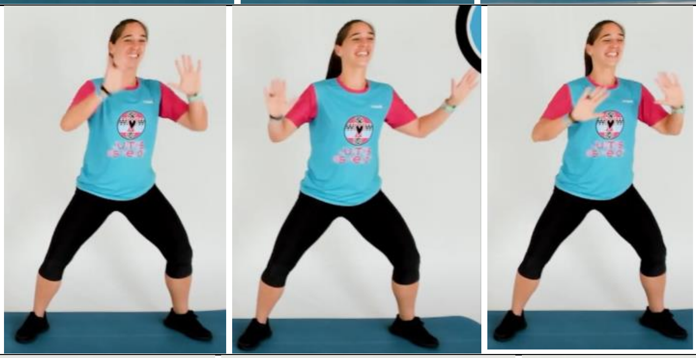 |
| Four-footed position + Knee lift + side to side movement | 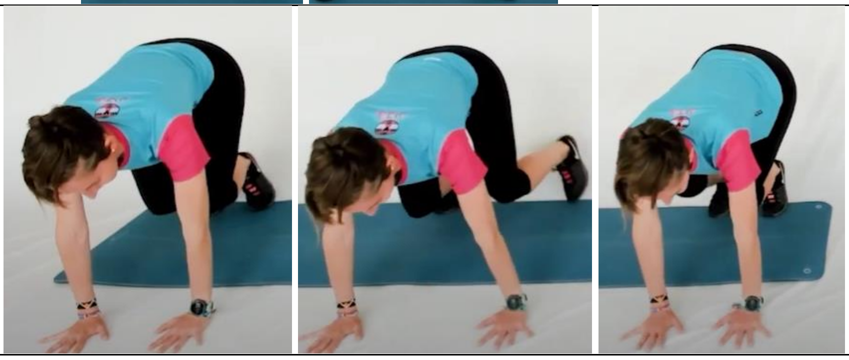 |

## *Week 1 - Session 2 (Link)*

| Exercise | Photo |
|---|---|
| Sprint + skipping | 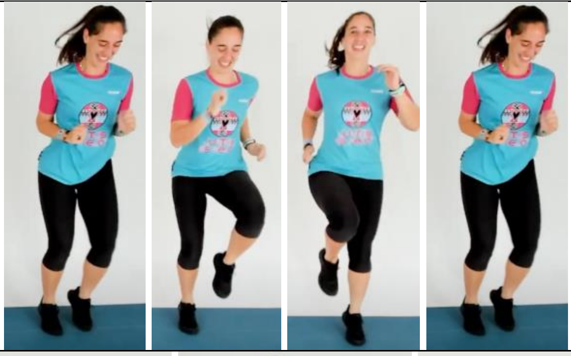 |
| 2x prisoner squat + lateral jump | 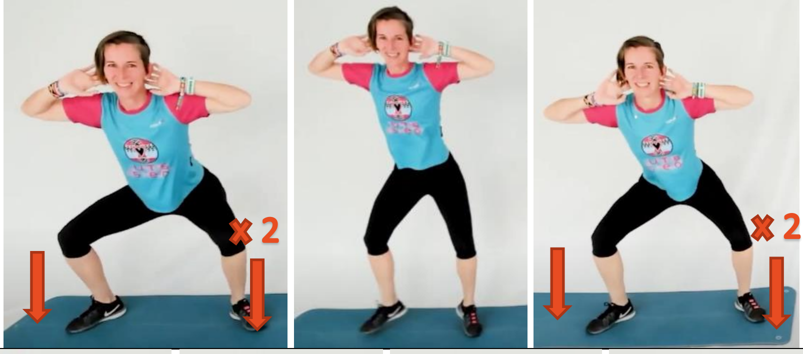 |
| Rotation + arms raise with hands in front | 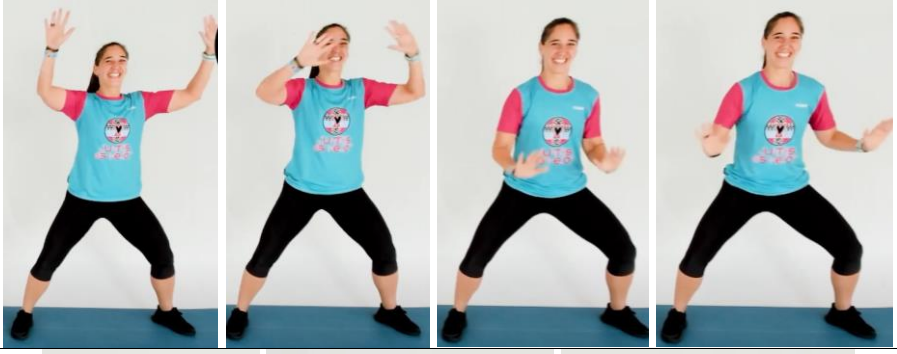 |
| Four-footed position + knee lift + side to side + downward facing dog | 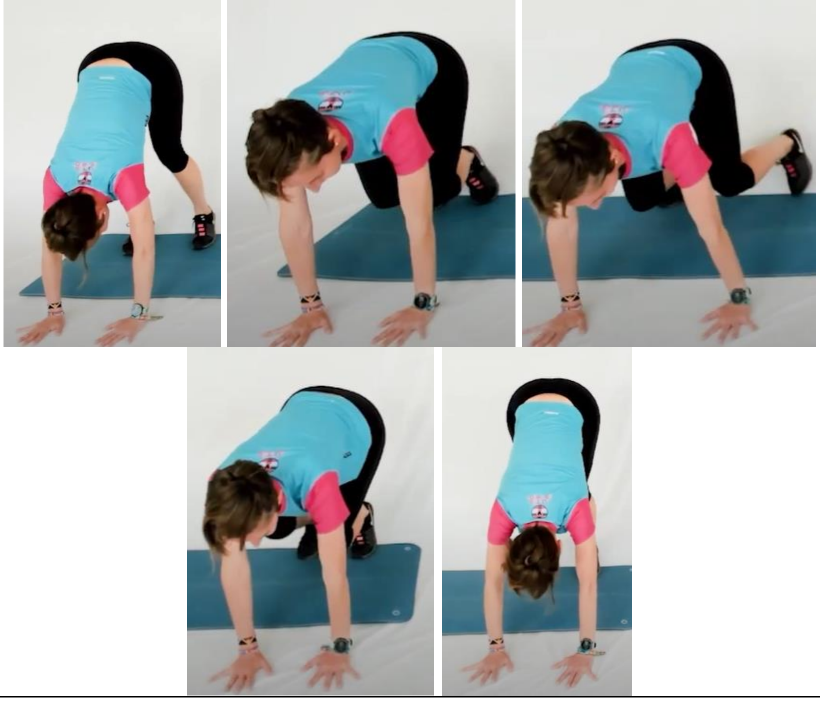 |

## *Week 2 - Session 1 (Link)*

| Exercise | Photo |
|---|---|
| Jumping + touch opposite foot | 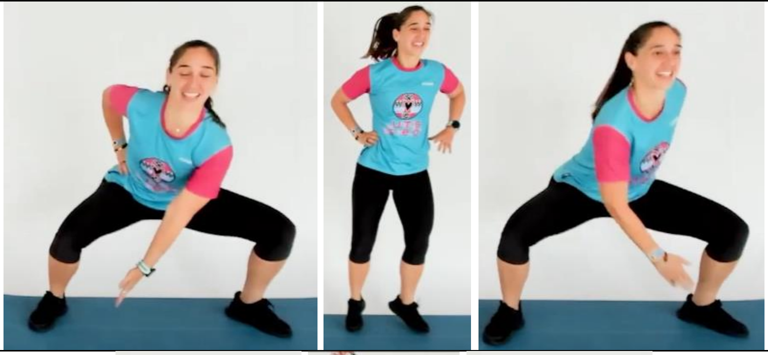 |
| Jump squat + beat both arms down center | 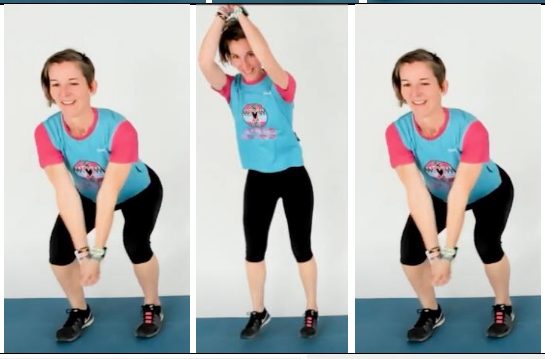 |
| Push up in four-footed position + side to side | 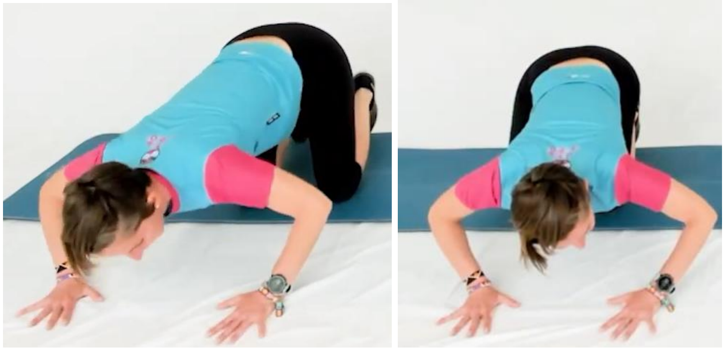 |
| Lateral plank up and down + arm | 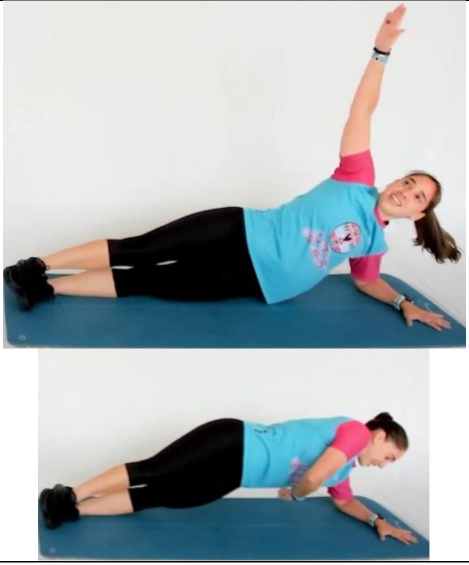 |

## *Week 2 - Session 2 (Link)*

| Exercise | Photo |
|---|---|
| Jumping + Diagonal arm elevation | 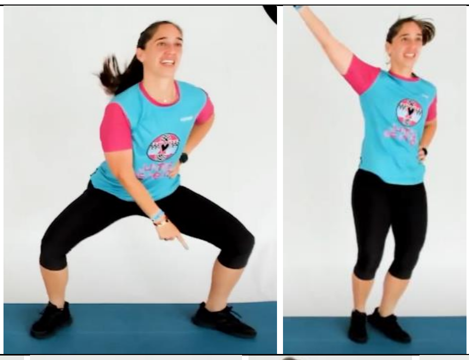 |
| Jump squad + beat both arms down side to side | 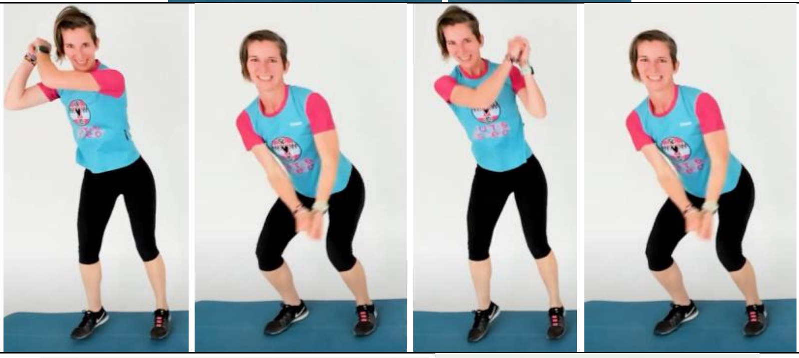 |
| Push up X2 in four-footed position + side to side | 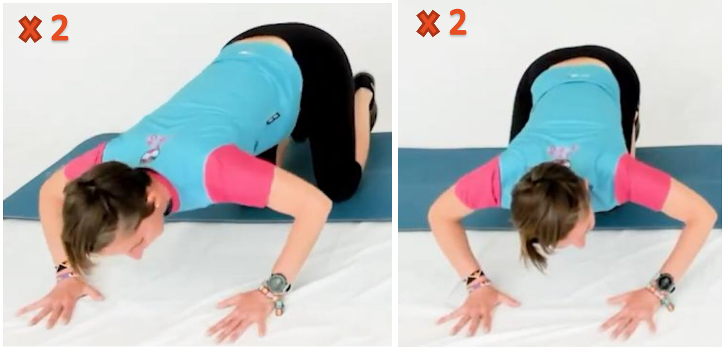 |
| Lateral plank + arm cross | 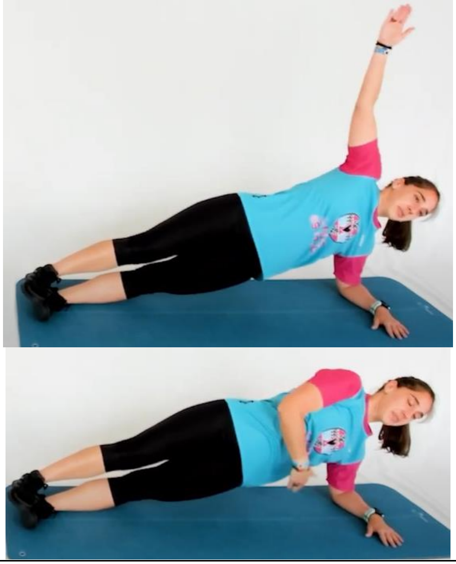 |

## *Week 3 - Session 1 (Link)*

| Exercise | Photo |
|---|---|
| 3X jump + 1 jumping close and open legs | 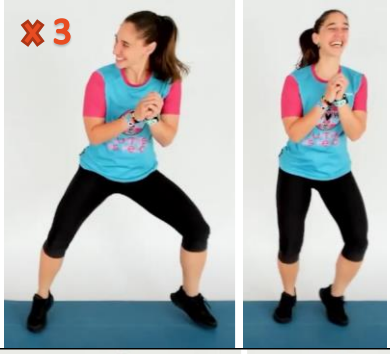 |
| Side squat + touch opposite knee | 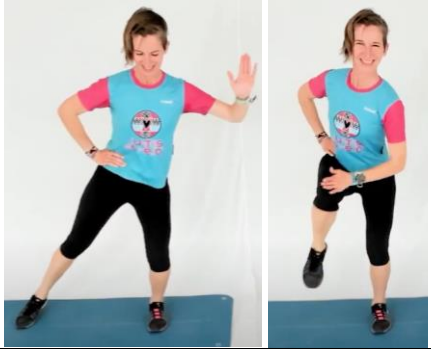 |
| Opening of arms with trunk flexion in front | **Level 1**<br>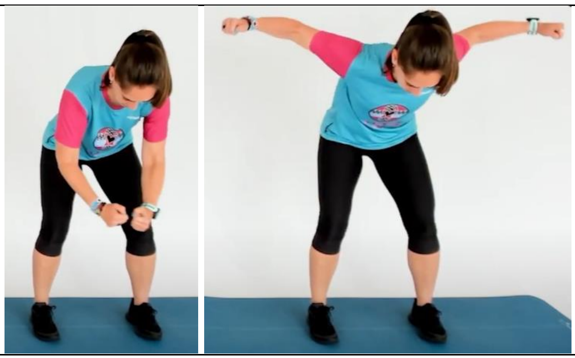<br>**Level 2**<br>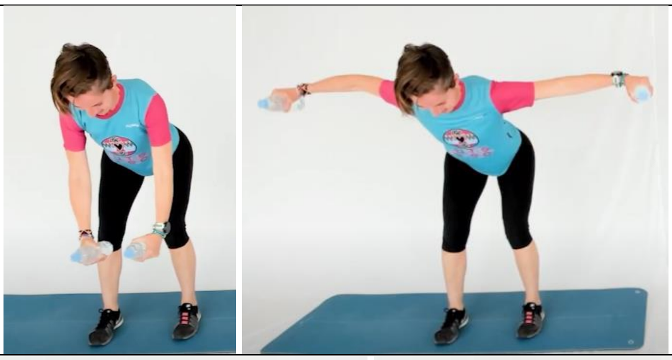 |
| Plank + downguard facing dog touching the opposite foot to foot | 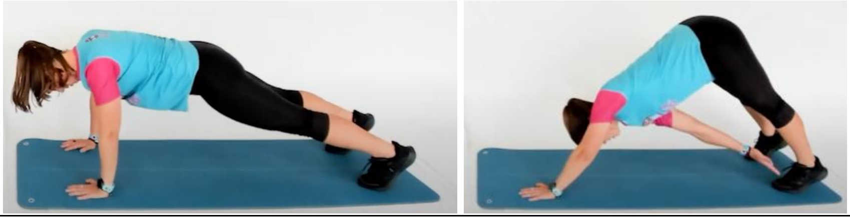 |

| | |
|---|---|
| | 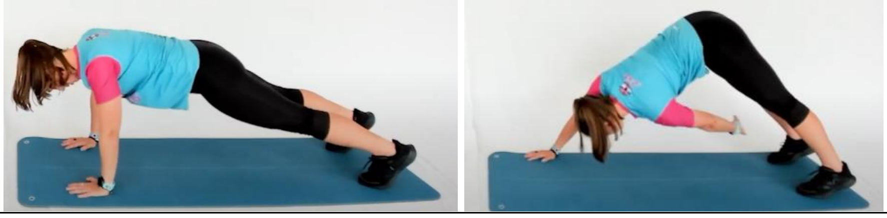 |

## *Week 3 - Session 2 (Link)*

| Exercise | Photo |
|---|---|
| 3x jumps + 3 jumps with knees up | **Level 1**<br>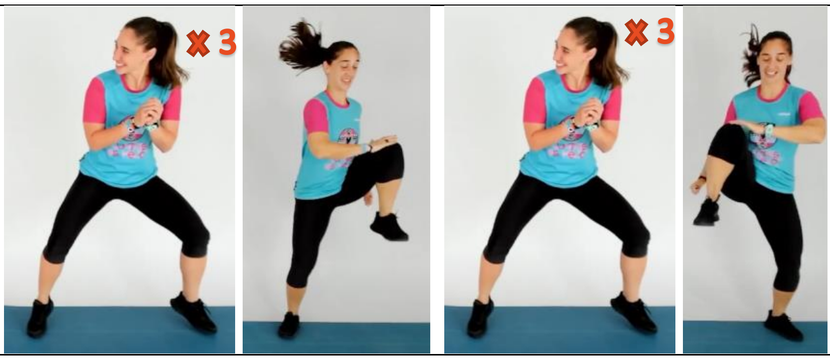<br><br>**Level 2**<br>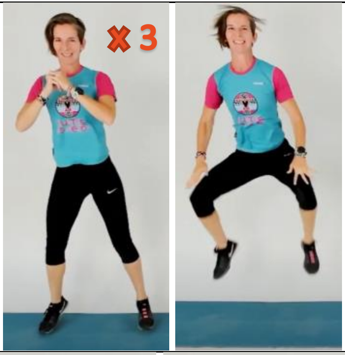<br> |
| Side squat + Touching opposite knee + foot closes | 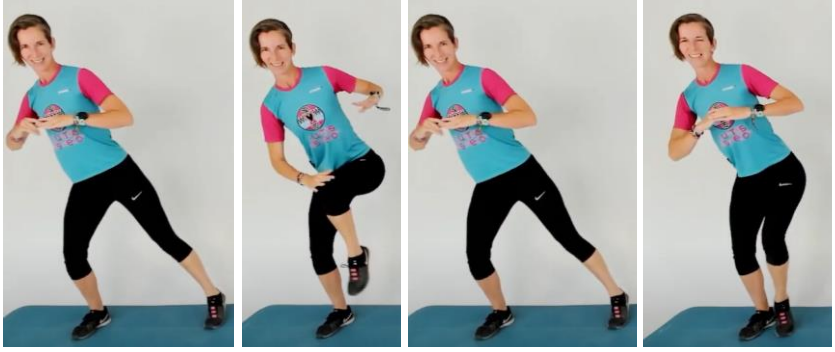 |

| | |
|---|---|
| Arm opening + front lifting of arms with front trunk flexion | **Level 1**<br>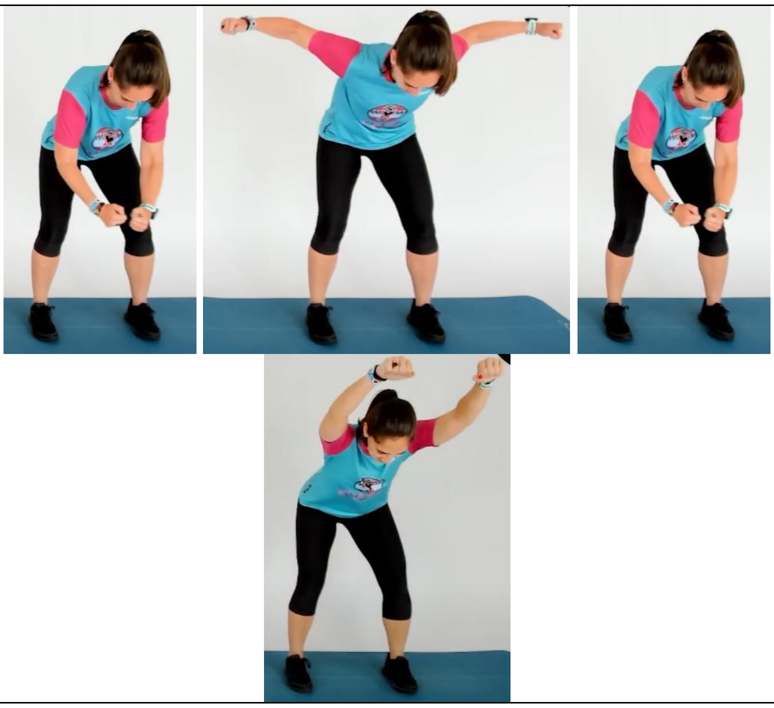<br>**Level 2**<br>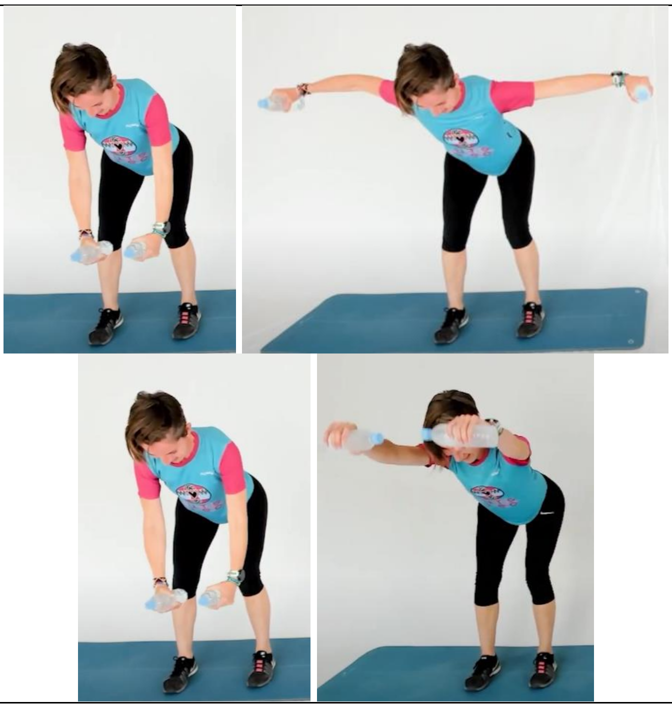 |
| | **Level 1**<br>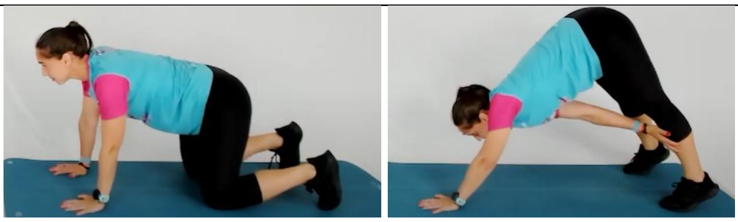 |

| | |
|---|---|
| Plank touching opposite shoulder + downguard facing dog touching opposite hand to foot | 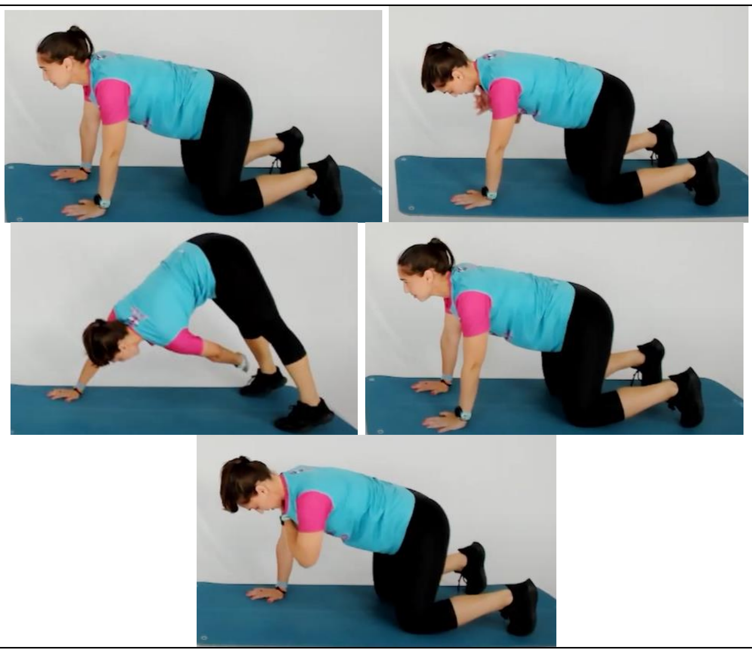 |
| | **Level 2** |
| | 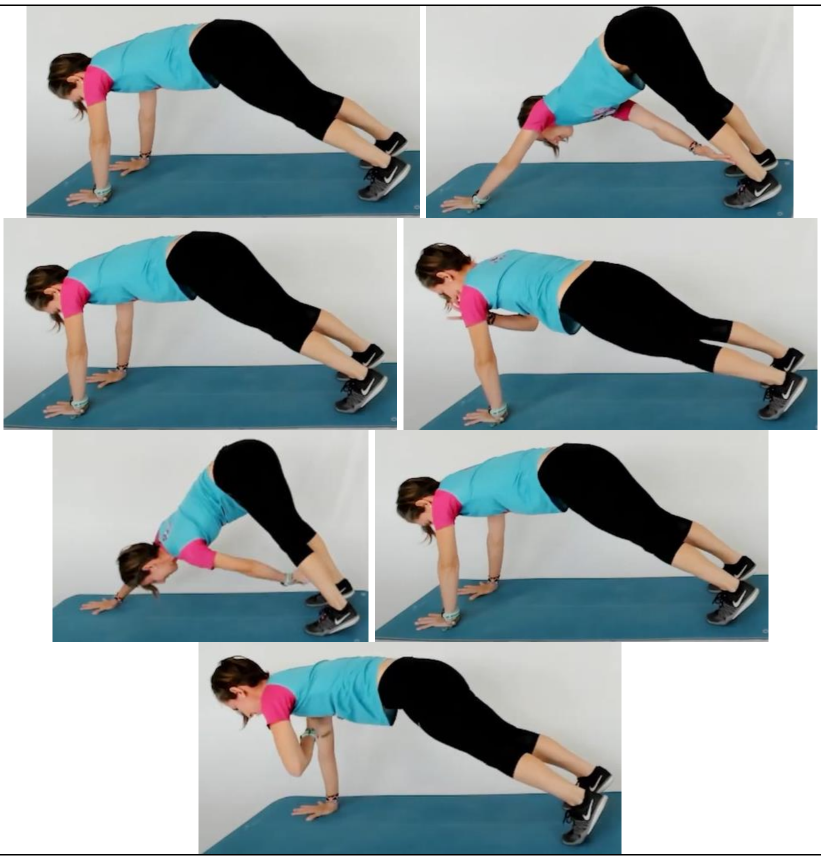 |

## *Week 4 - Session 1 (Link)*

| Exercise | Photo |
| --- | --- |
| | **Level 1** |
| Kicks with jump | 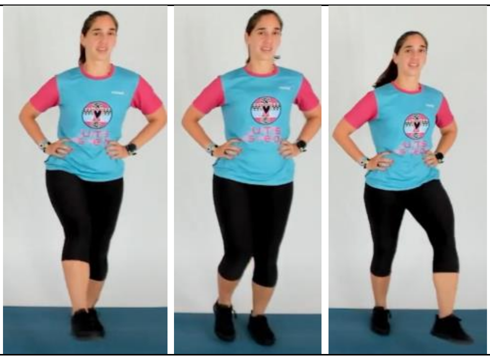 |
| | **Level 2** |
| | 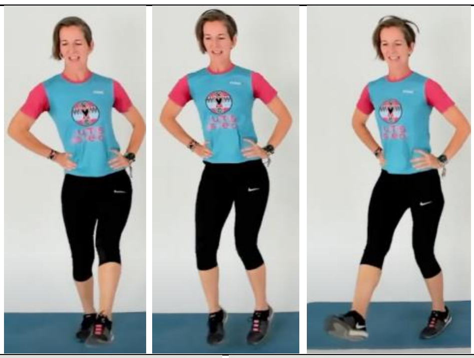 |
| Prisioner squat | 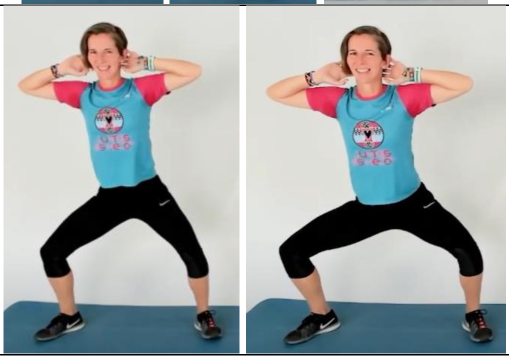 |
| | **Level 1** |
| Hook punch | 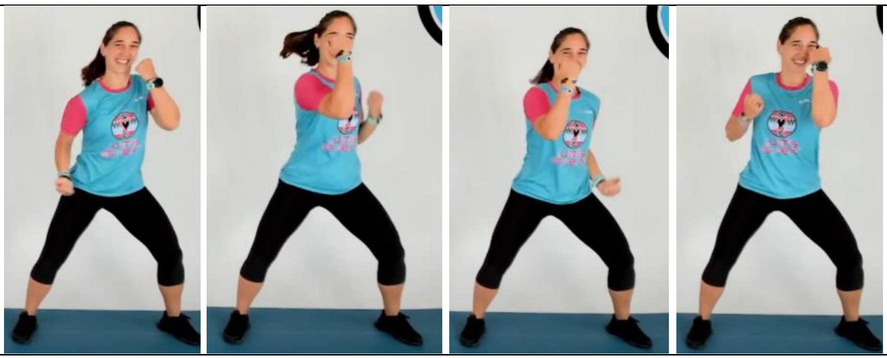 |
| | **Level 2** |

| | |
|---|---|
| | 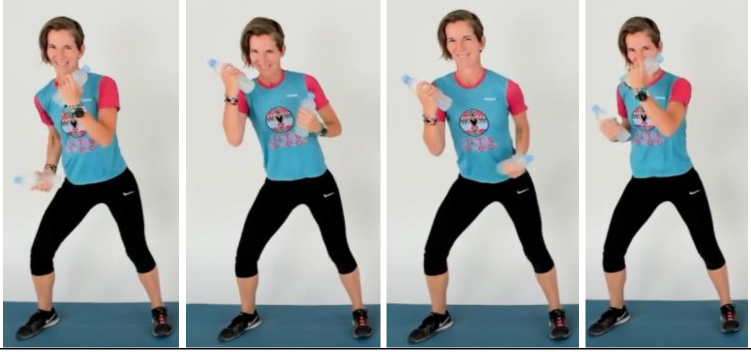 |
| Side to side push arms with intertwined hands | **Level 1** |
| | 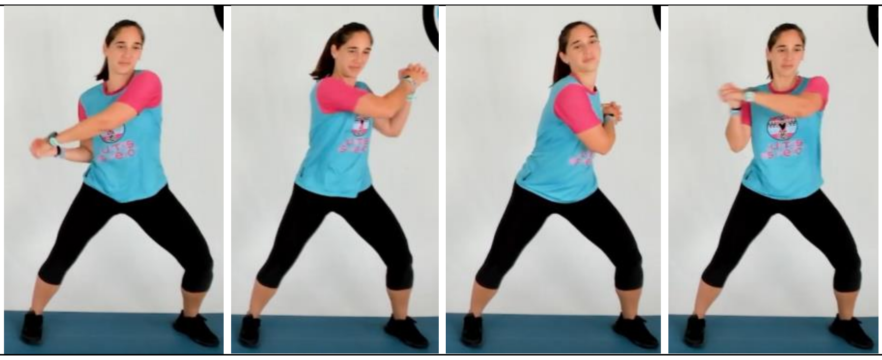 |
| | **Level 2** |
| | 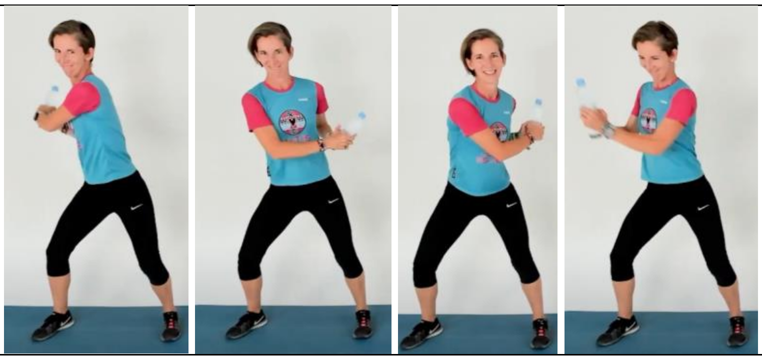 |

## *Week 4 - Session 2 (Link)*

| **Exercise** | **Photo** |
|---|---|
| Kicks + arm extension up | **Level 1** |
| | 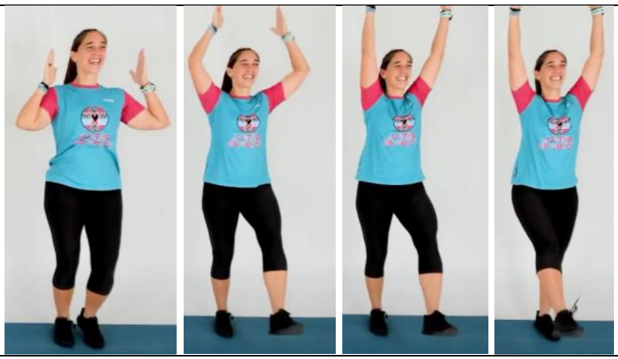 |
| | **Level 2** |

| | |
|---|---|
| | 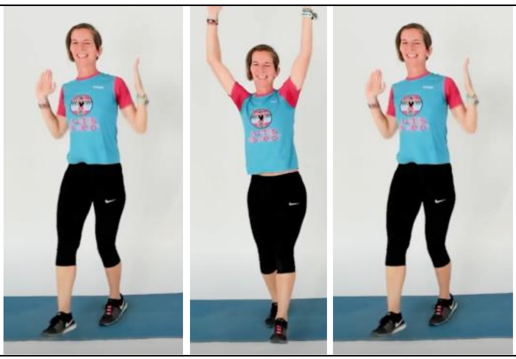 |
| Prisoner squat to side | **Level 1**<br>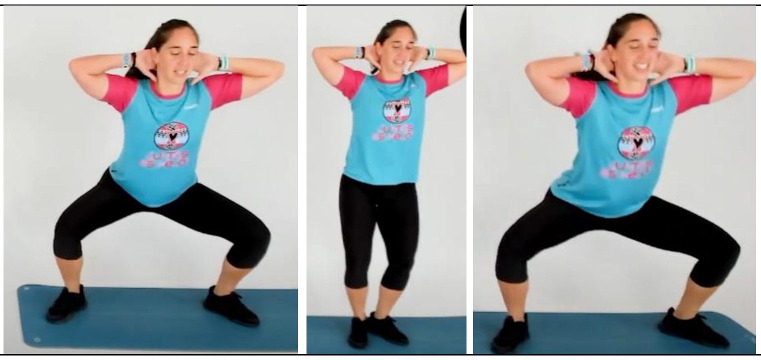<br>**Level 2**<br>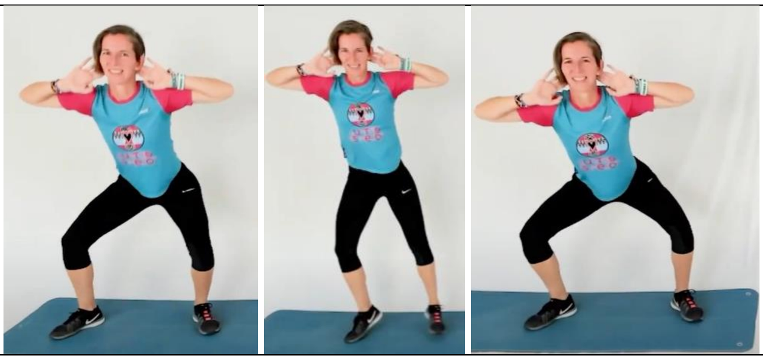 |
| Hook and punch | **Level 1**<br>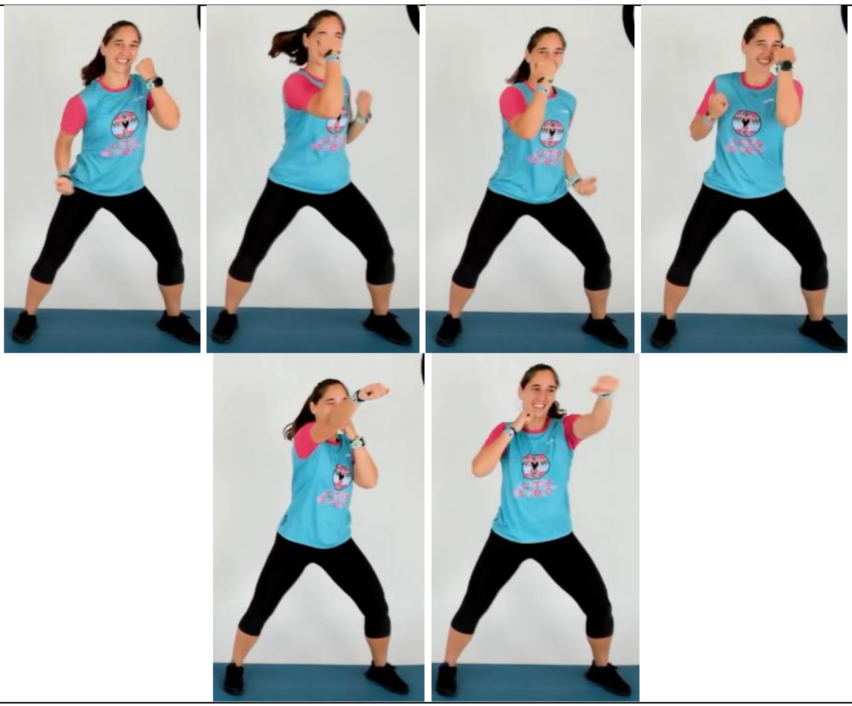 |

| | |
|---|---|
| | **Level 2**<br>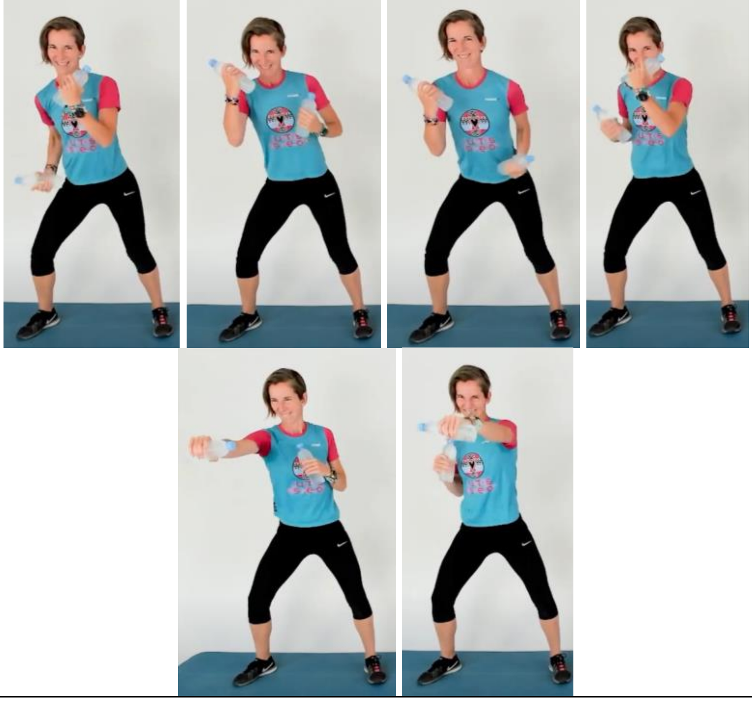 |
| Side to side arms push with intertwined hands and lateral movement | **Level 1**<br>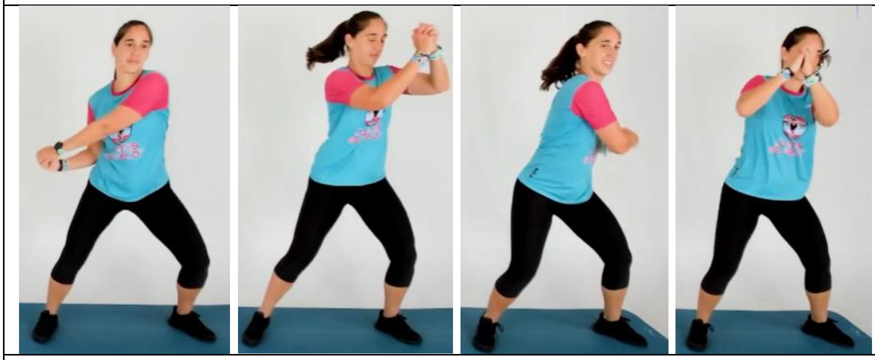<br>**Level 2**<br>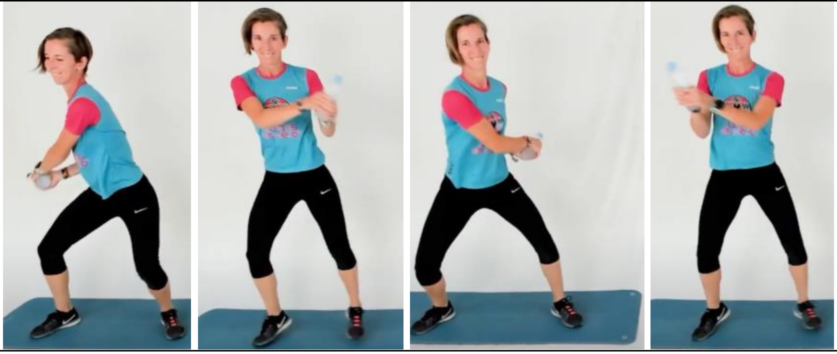 |

# Month 6 (40" of work - 15" of recovery)

## Common Part

*Dynamic Warm-up (At the beginning of each session before the main part) (Link)*

| Exercise | Photograph |
|---|---|
| Neck flexion and extension, neck torsion | 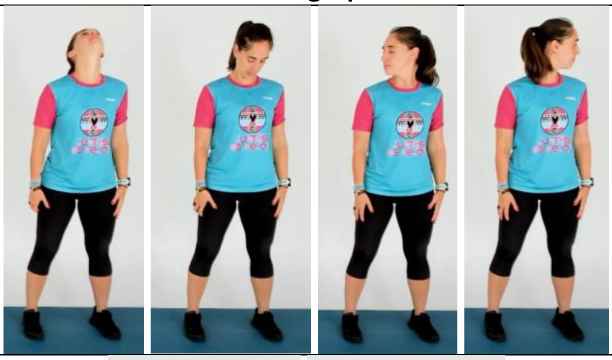 |
| Hands to opposite elbows above the head and lateral column tilt | 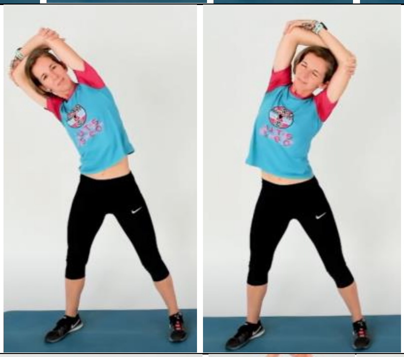 |
| Arms balance, down and side | 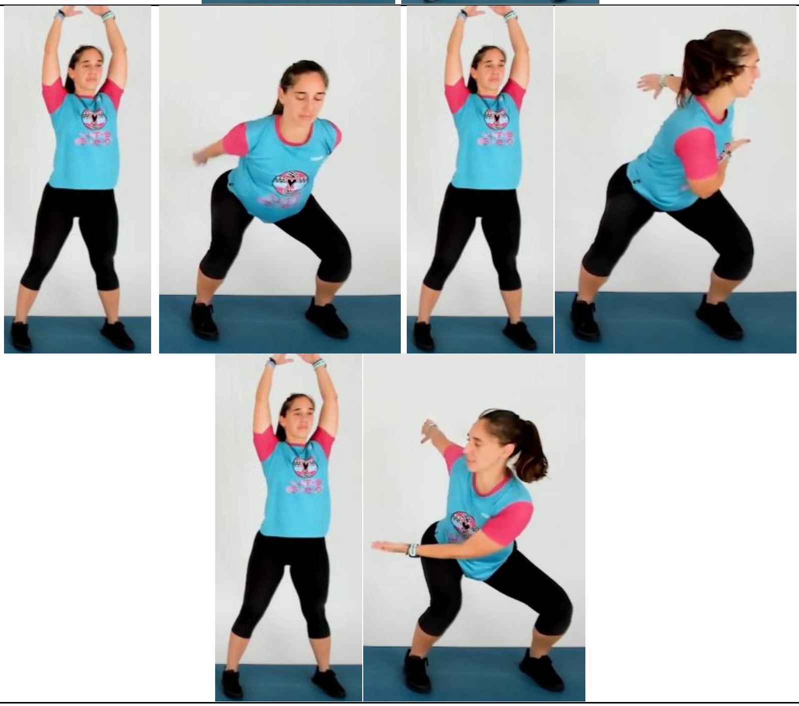 |

| | |
|---|---|
| One leg extension in front with arms down | 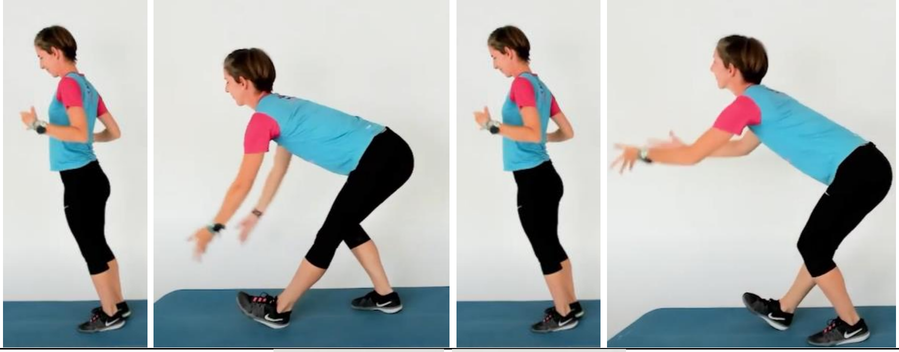 |
| Hip rotation | 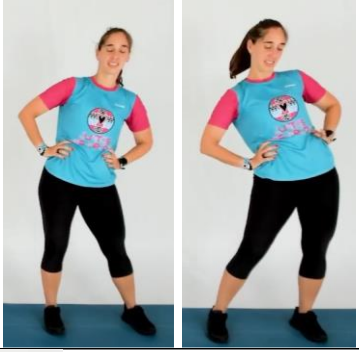 |
| Lateral movement + arms extension up and down | 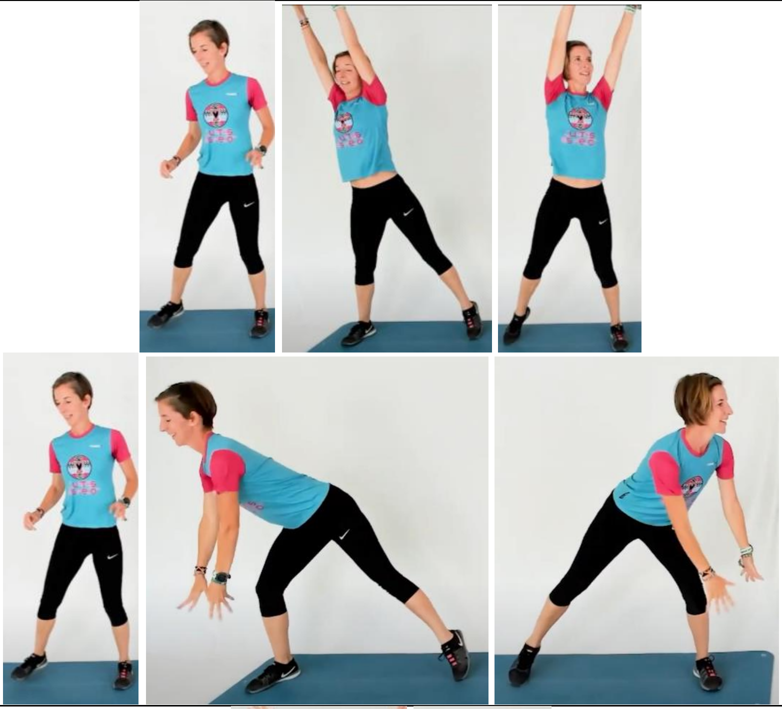 |
| Sprint + open and close legs + up and down arms | 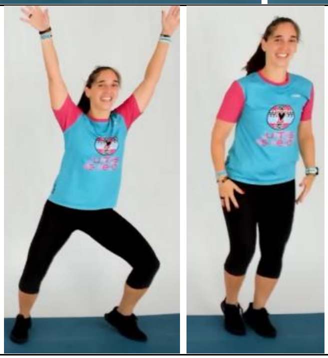 |

*Stretching (At the end of each session after the main part) (Link)*

| Stretch | Photograph |
|---|---|
| Supine position: arm extension | 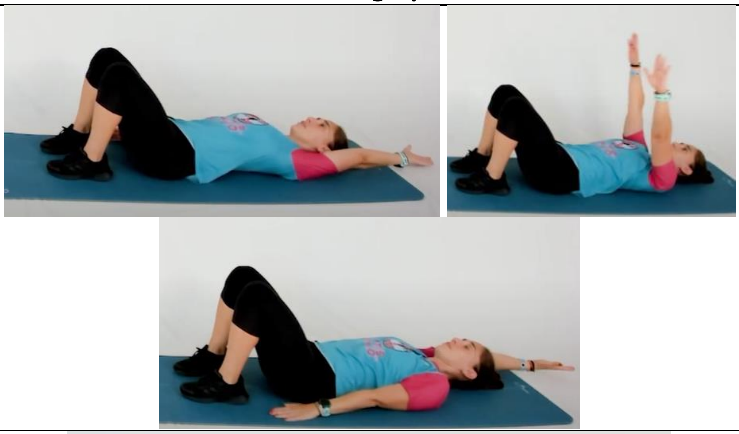 |
| Hand to opposite knee and column rotation | 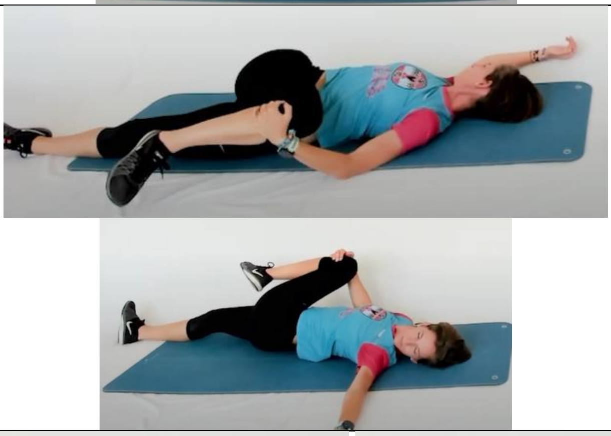 |
| Cross one leg and catch down leg to the chest | 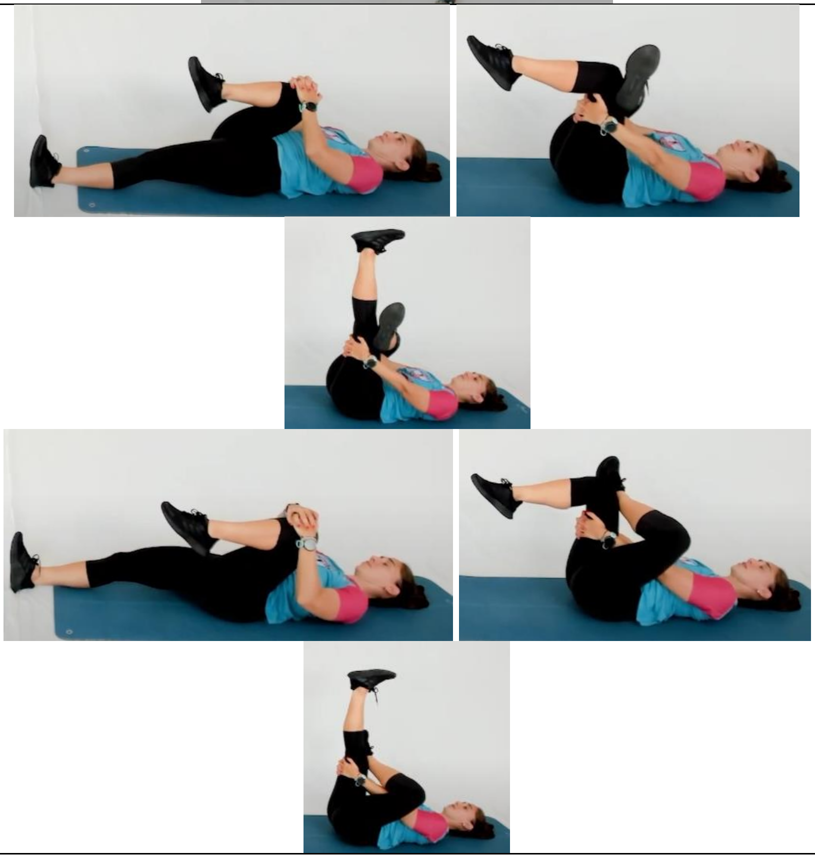 |

<table>
<tr><td>2 legs flexion to the chest catching with hands + 2 legs extension with arms extension</td><td>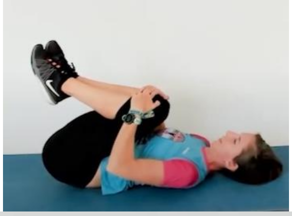<br>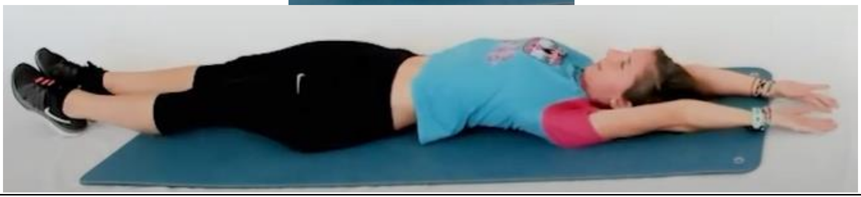</td></tr>
</table>

# Main part (4 rounds of 4 exercises with 1’ rest in between rounds) (40’’ Work – 15’’ Rest)

*Week 1 - Session 1 (Link)*

<table>
<tr><th>Exercise</th><th>Photo</th></tr>
<tr><td rowspan="4">Jump rope with foot taps</td><td>Level 1</td></tr>
<tr><td>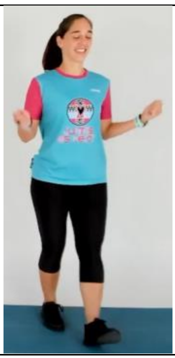 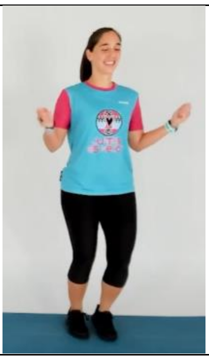 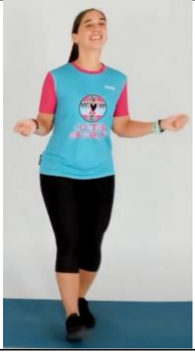</td></tr>
<tr><td>Level 2</td></tr>
<tr><td>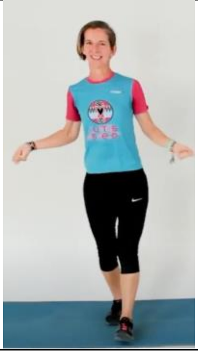 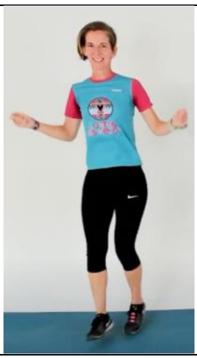 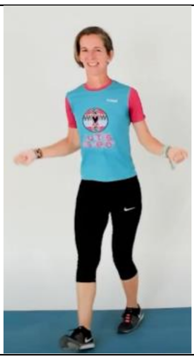</td></tr>
</table>

| | |
|---|---|
| | **Level 1** |
| Toe touch with opposite hand and knee touch with opposite hand | 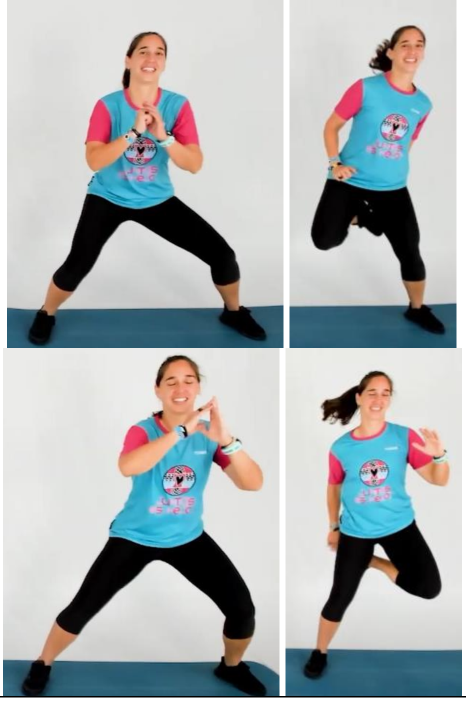 |
| | **Level 2** |
| | 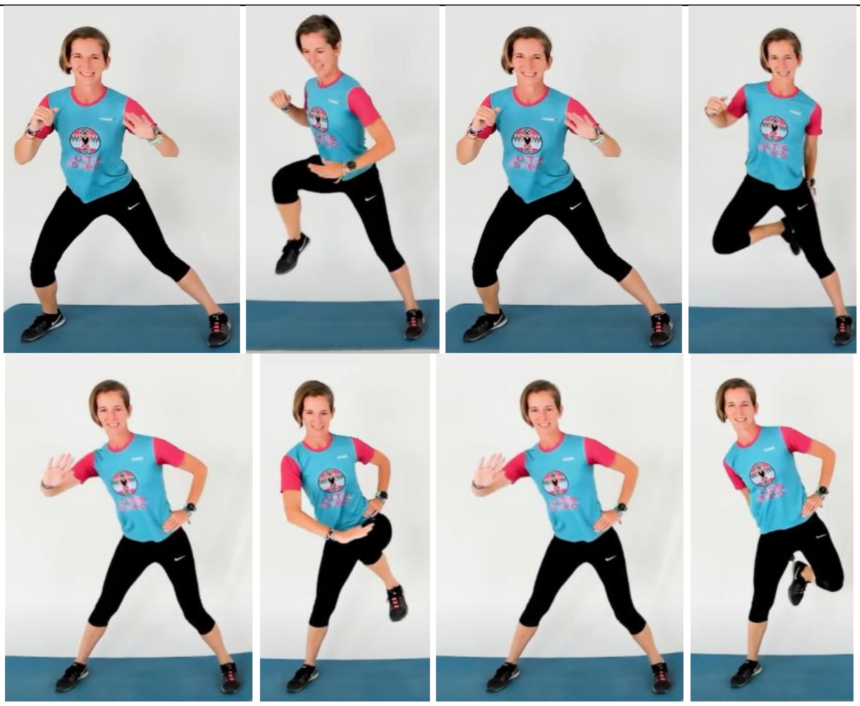 |

<table>
<tr><td rowspan="4">Arm rotation in abduction and other arm in straight angle</td><td>Level 1</td></tr>
<tr><td>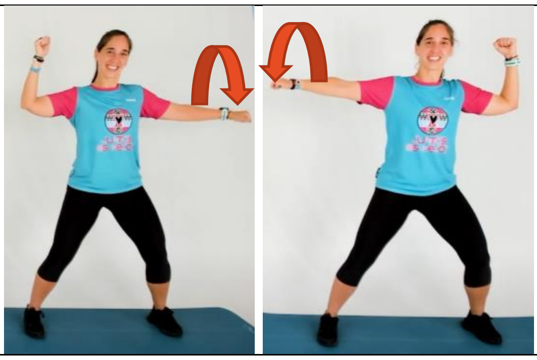</td></tr>
<tr><td>Level 2</td></tr>
<tr><td>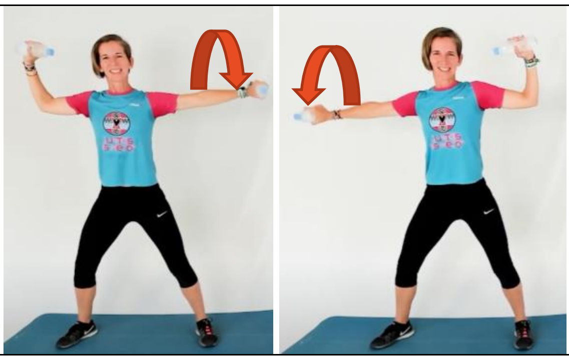</td></tr>
<tr><td rowspan="4">Sit down and punch</td><td>Level 1</td></tr>
<tr><td>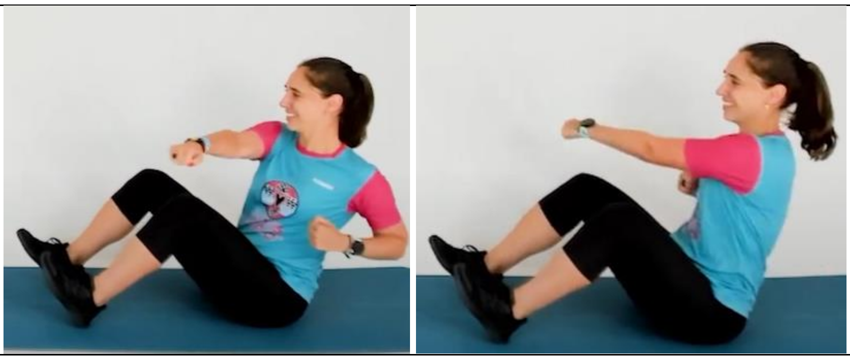</td></tr>
<tr><td>Level 2</td></tr>
<tr><td>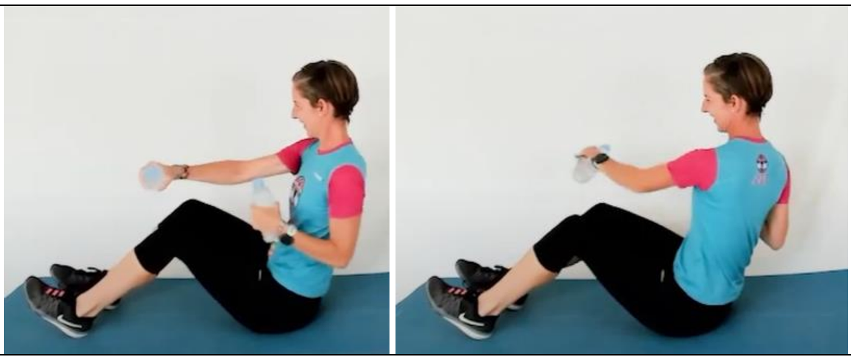</td></tr>
</table>

## *Week 1 - Session 2 (Link)*

| Exercise | Photo |
|---|---|
| Jump rope with tap and crossing arms | **Level 1** |
| | 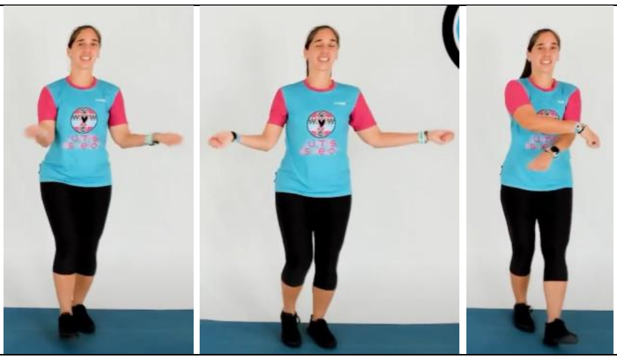 |
| | **Level 2** |
| | 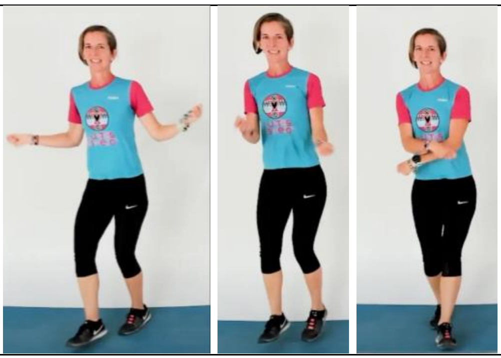 |
| Toe touch with opposite hand and lateral kick | **Level 1** |
| | 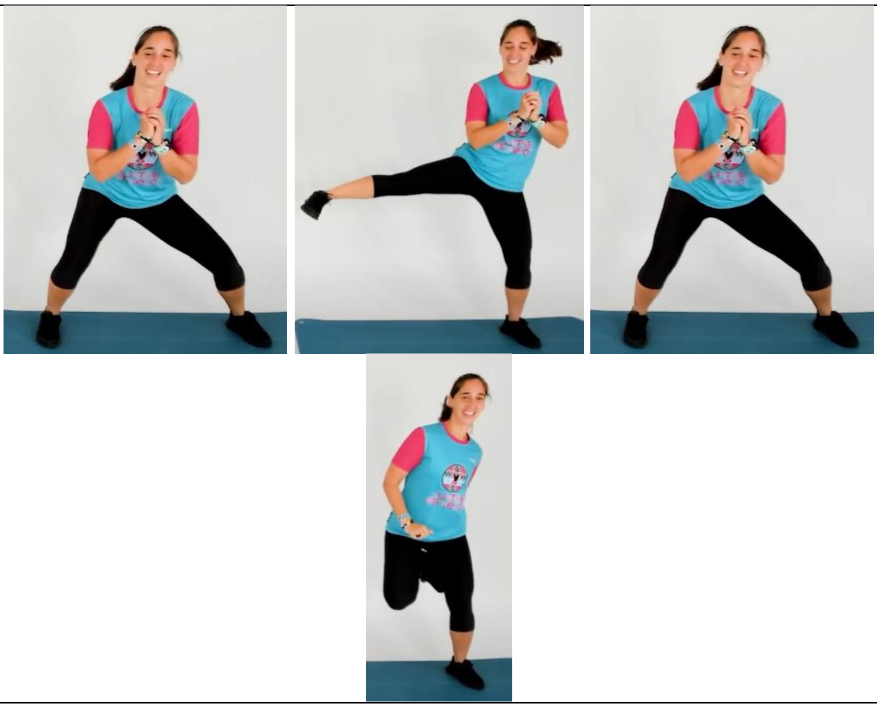 |

| | |
|---|---|
| | **Level 2**<br>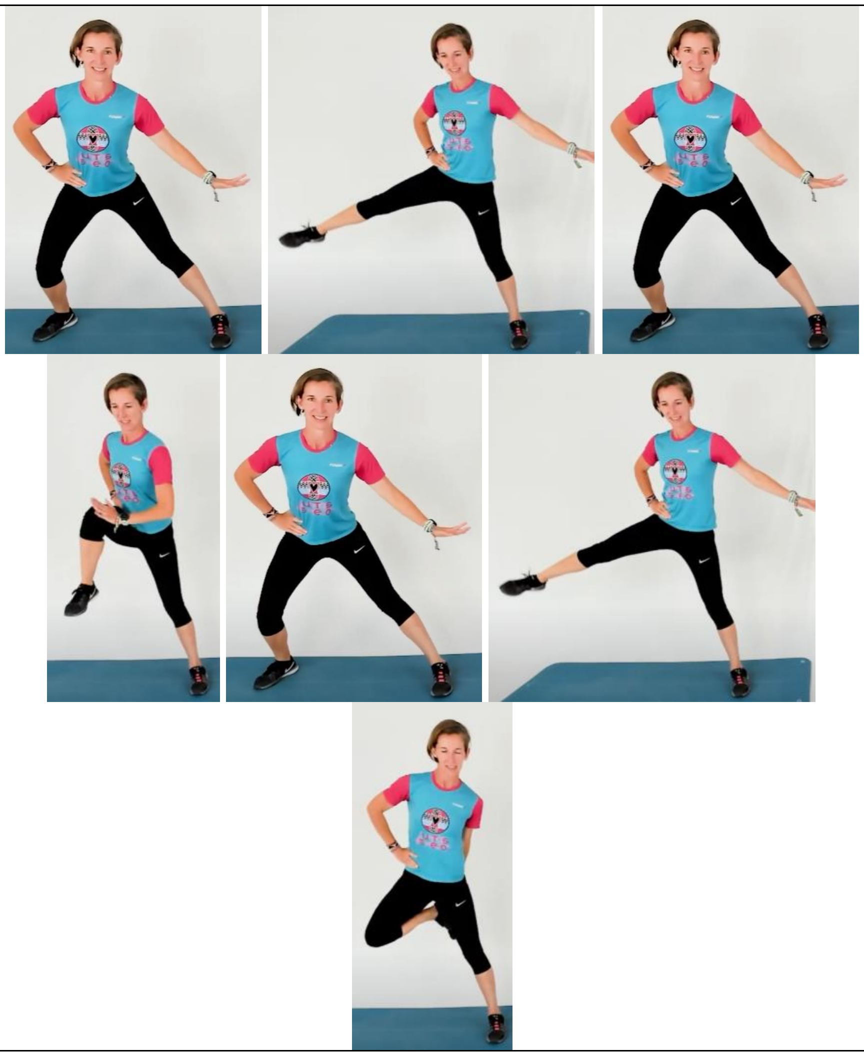 |
| Arm rotation in abduction and other arm in straight angle to up extension | **Level 1**<br>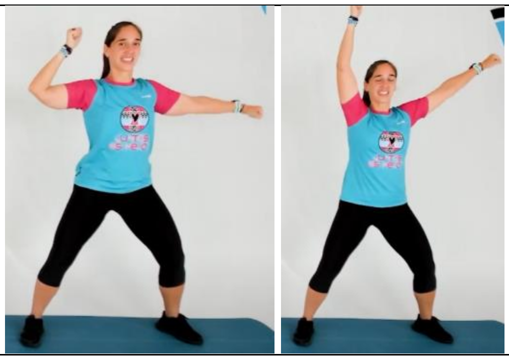<br>**Level 2**<br>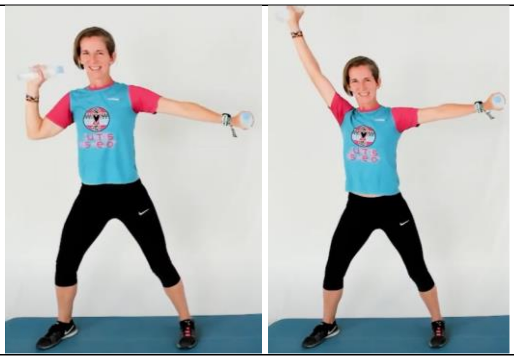 |

| | |
|---|---|
| Sit down and punch with feet without touching the ground | **Level 1** |
| | 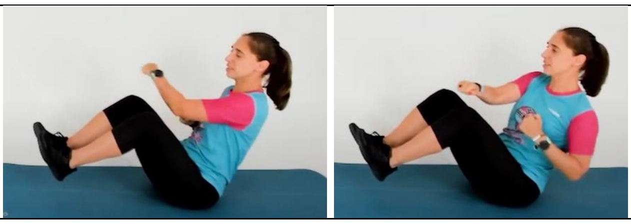 |
| | **Level 2** |
| | 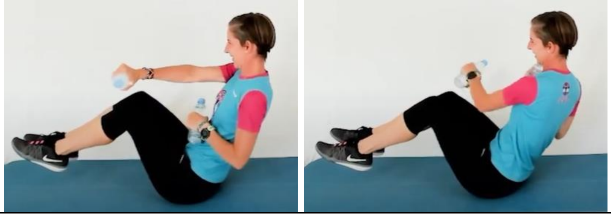 |

## *Week 2 - Session 1 (Link)*

| Exercise | Photo |
|---|---|
| Back tap in half squat + jump | **Level 1** |
| | 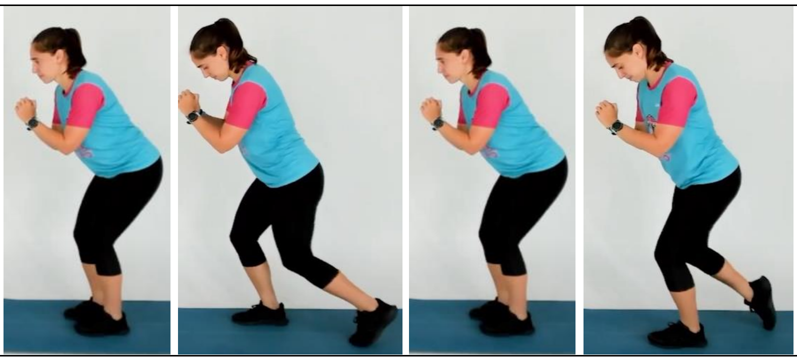 |
| | **Level 2** |
| | 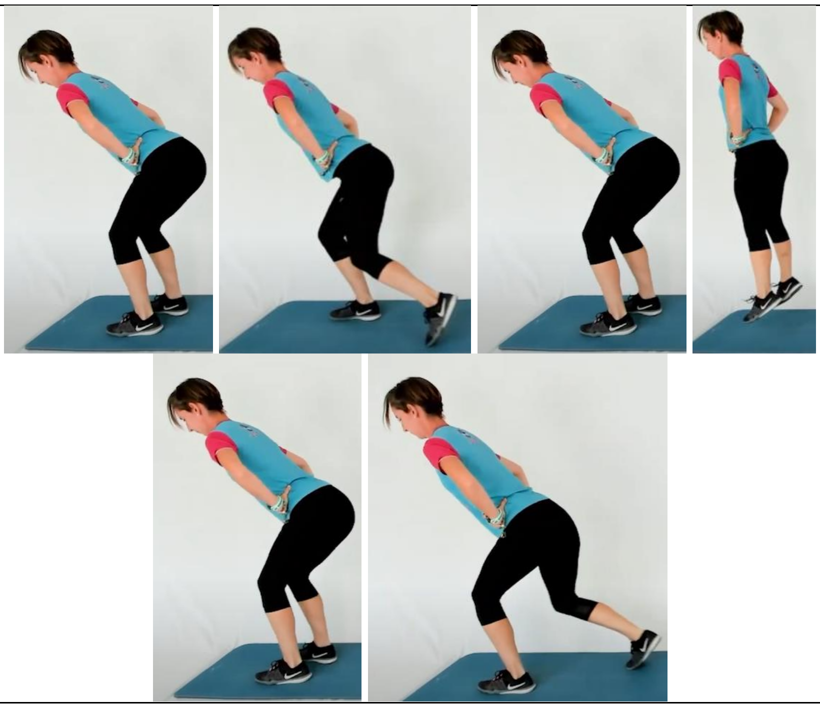 |

<table>
<tr><td rowspan="4">Lunge + squat jump + jump</td><td>Level 1</td></tr>
<tr><td>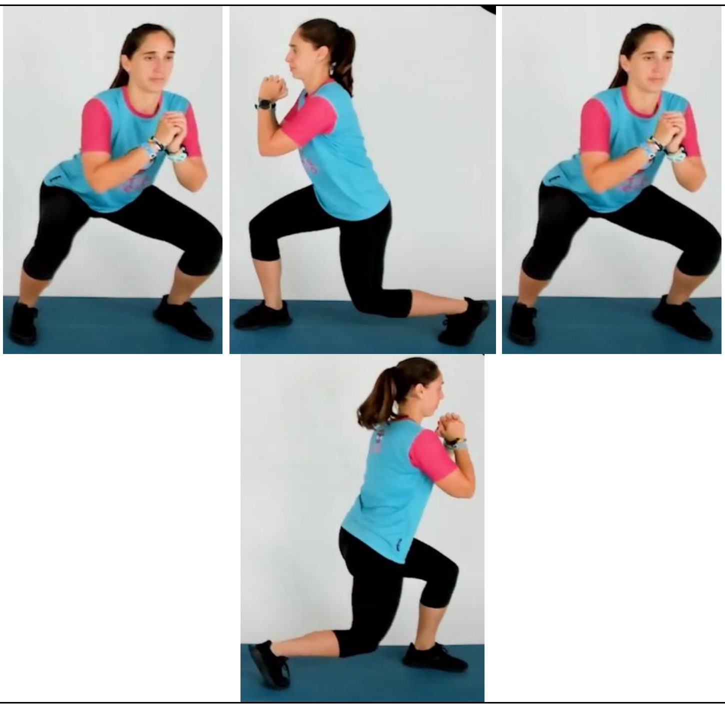</td></tr>
<tr><td>Level 2</td></tr>
<tr><td>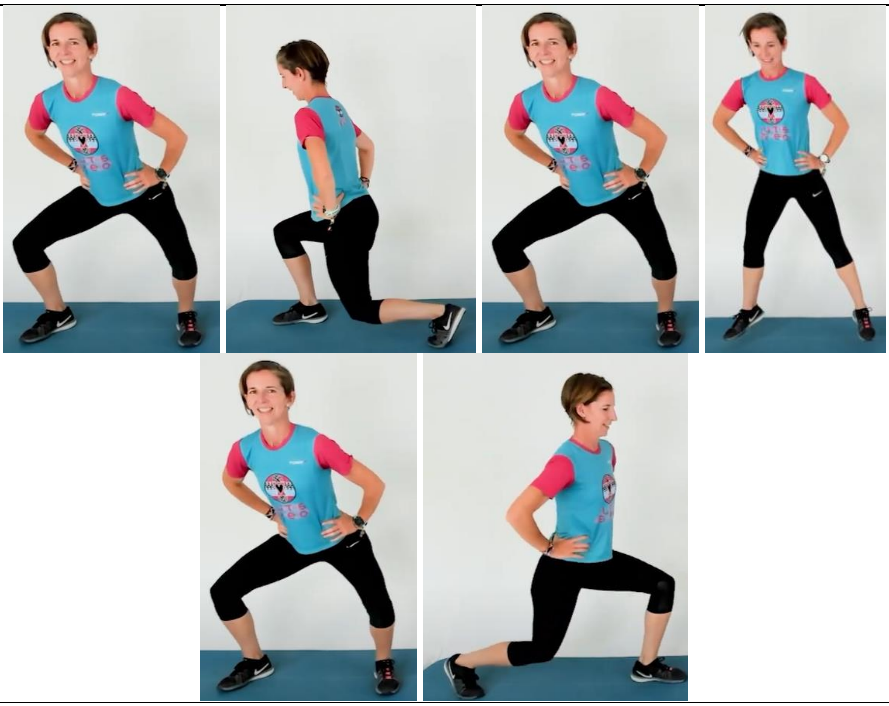</td></tr>
<tr><td rowspan="2">Arm rotation in straight angle</td><td>Level 1</td></tr>
<tr><td>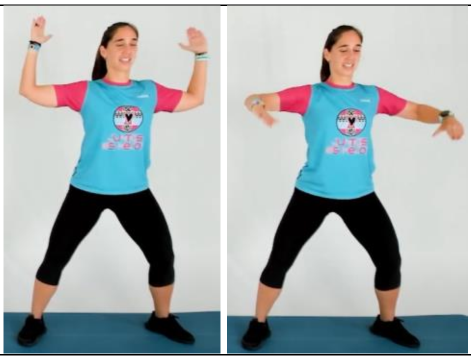</td></tr>
</table>

| | |
|---|---|
| | **Level 2** |
| | 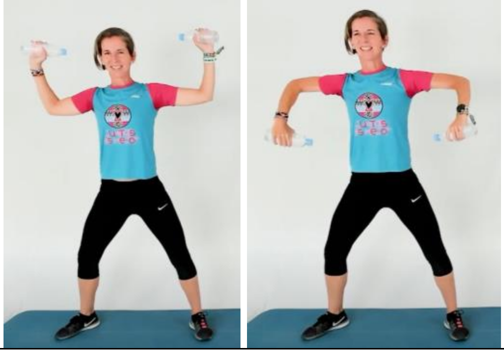 |
| Plank + 2 small steps + hip elevation | **Level 1** |
| | 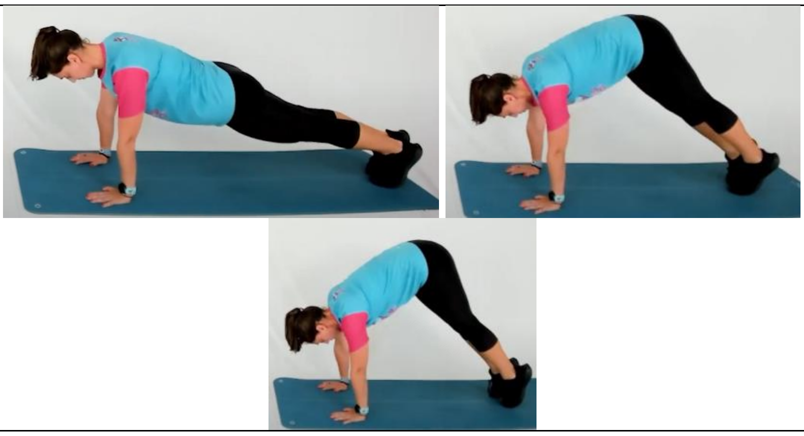 |
| | **Level 2** |
| | 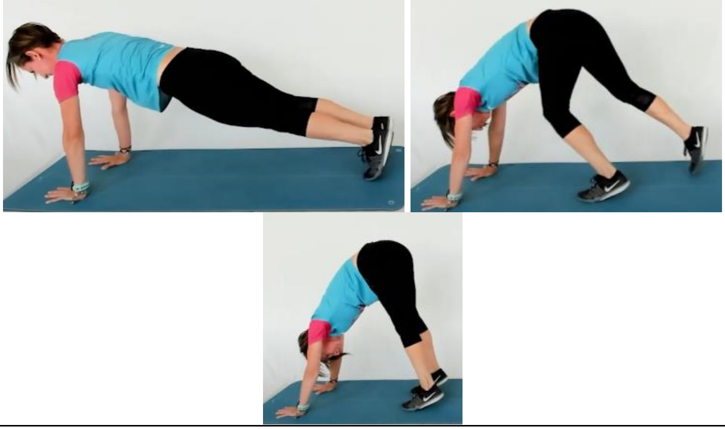 |

## *Week 2 - Session 2 (Link)*

| Exercise | Photo |
| --- | --- |
| Lunge jump + squat jump | **Level 1**<br>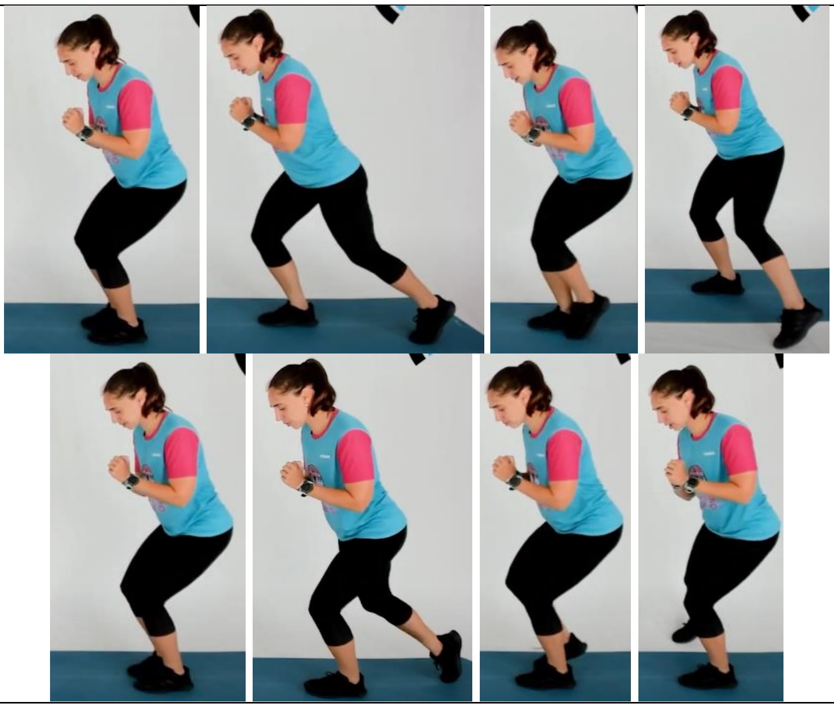<br>**Level 2**<br>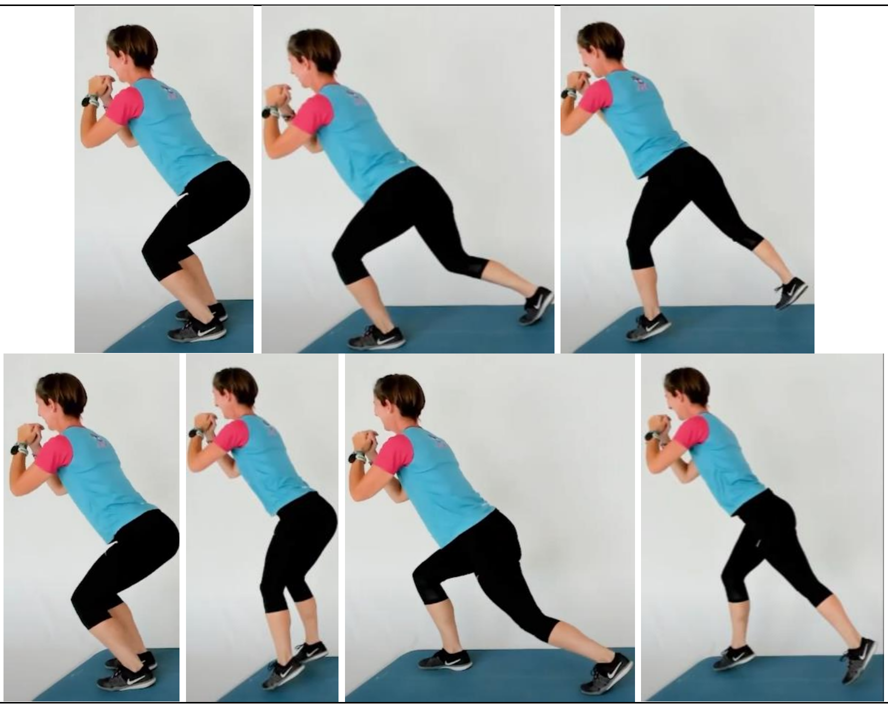 |

<table>
<tr><td rowspan="4">2 lunge jumps + 2 squat jumps</td><td>Level 1</td></tr>
<tr><td>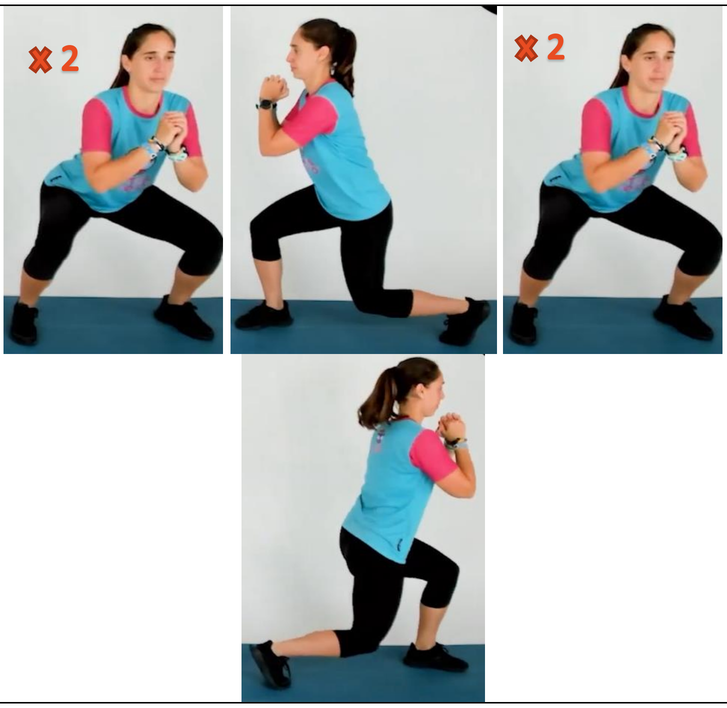
</td></tr>
<tr><td>Level 2</td></tr>
<tr><td>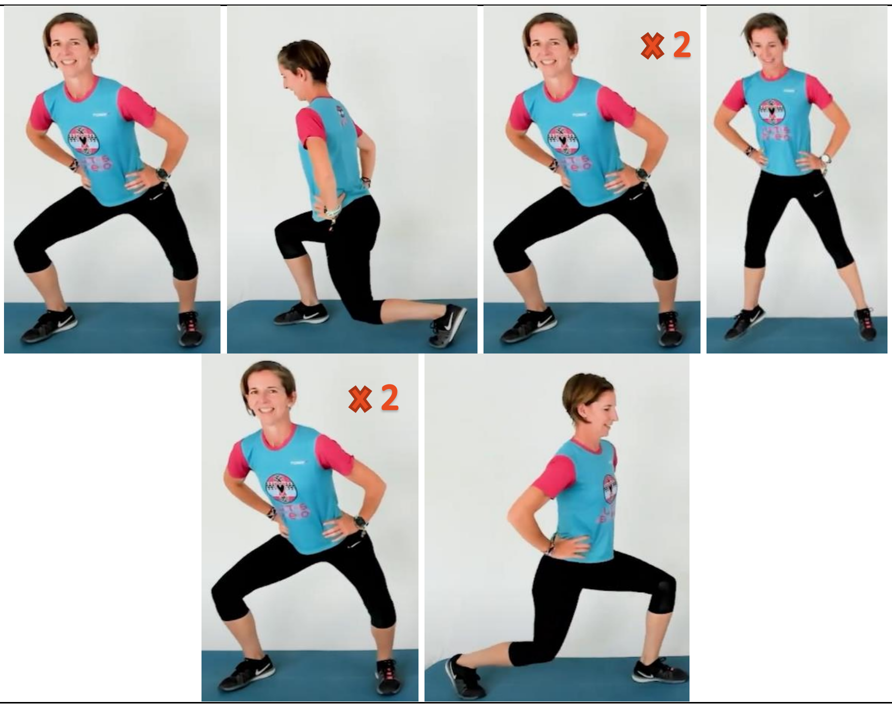
</td></tr>
<tr><td rowspan="2">Arm rotation in straight angle and up extension</td><td>Level 1</td></tr>
<tr><td>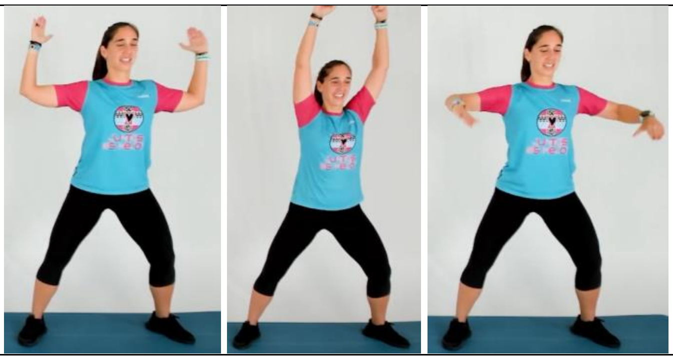</td></tr>
</table>

| | |
|---|---|
| | **Level 2**<br>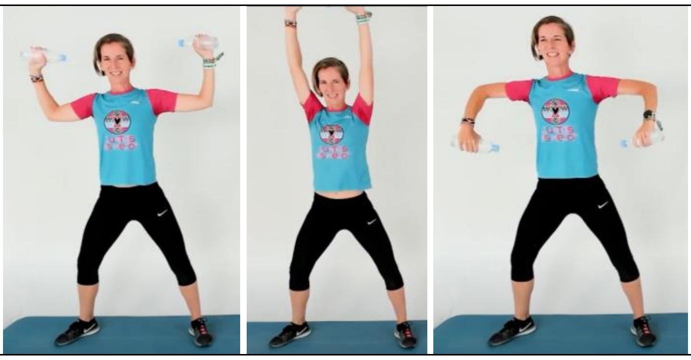 |
| Mountain climber + 2 steps + hip elevation | **Level 1**<br>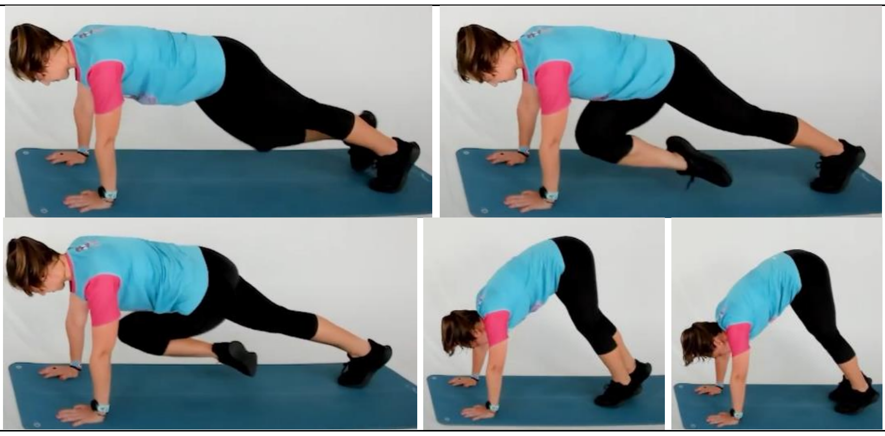<br>**Level 2**<br>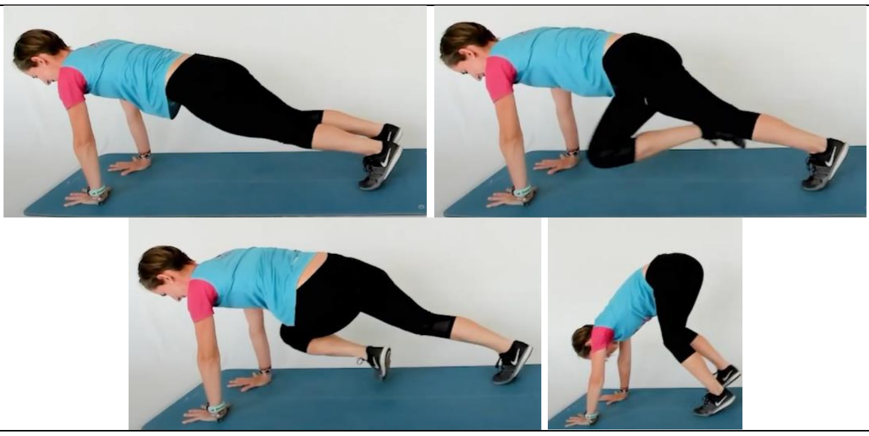 |

## *Week 3 - Session 1 (Link)*

| Exercise | Photo |
|---|---|
| | **Level 1** |
| Sumo squat jump and knee elevation to elbow touch | 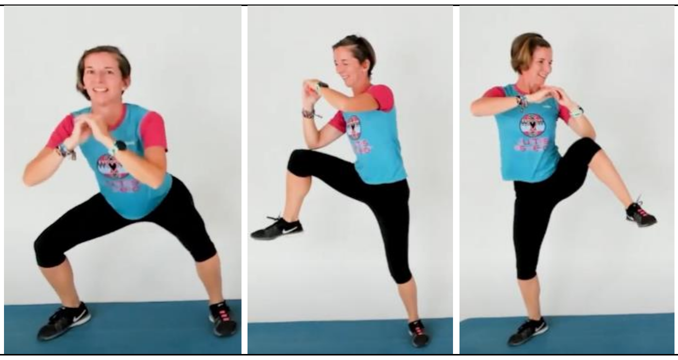 |
| | **Level 2** |
| | 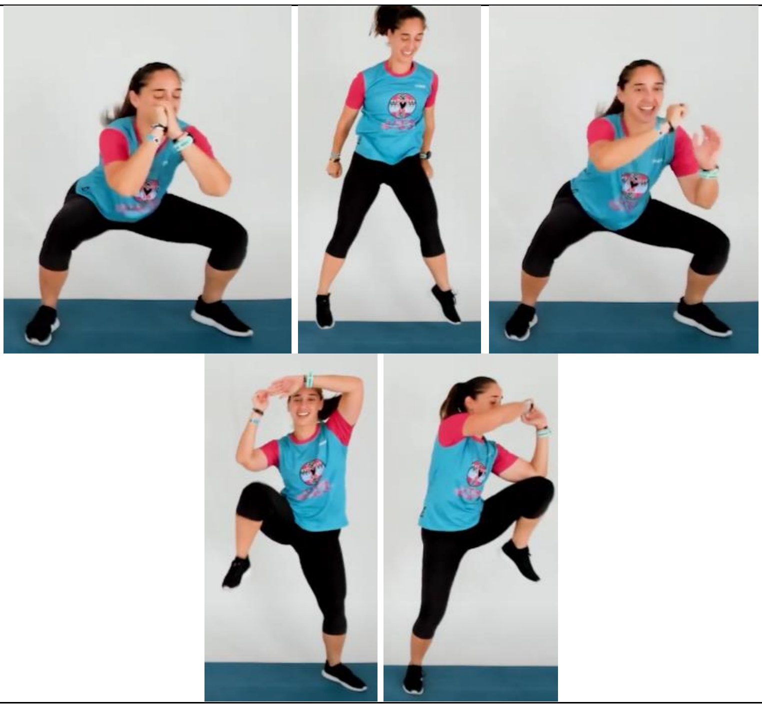 |
| | **Level 1** |
| Lateral lunge, step touching opposite toe | 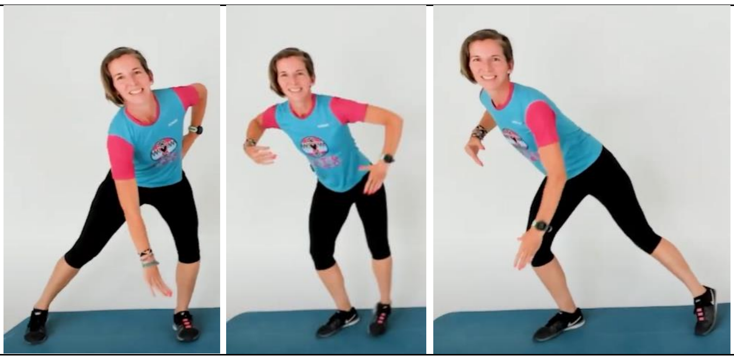 |

| | |
|---|---|
| | **Level 2** |
| | 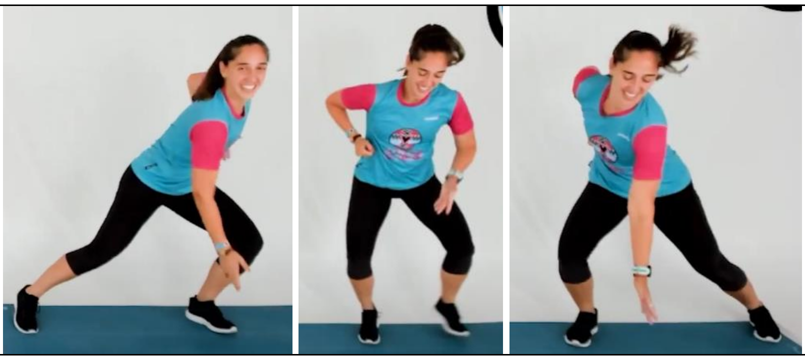 |
| Supine position: arms opening and closing | **Level 1** |
| | 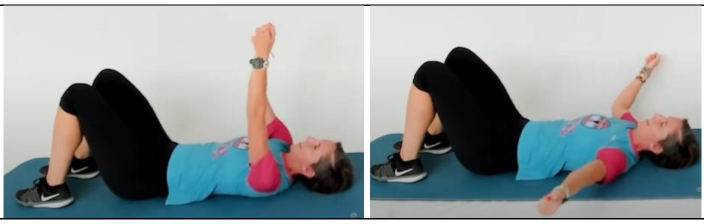 |
| | **Level 2** |
| | 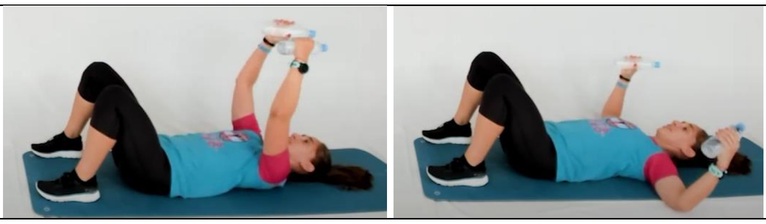 |
| Lateral plank + one leg touch front and back | **Level 1** |
| | 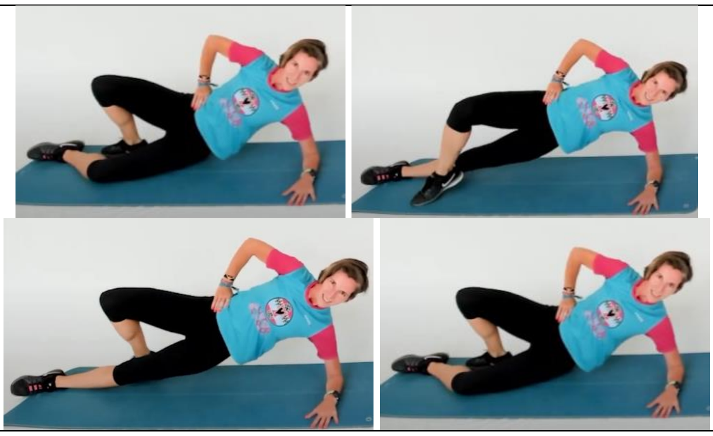 |
| | **Level 2** |
| | 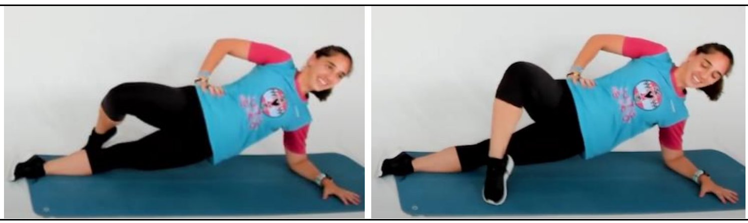 |

## *Week 3 - Session 2 (Link)*

| Exercise | Photo |
| --- | --- |
| | **Level 1** |
| Sumo squat jump side and knee elevation to touch elbow | 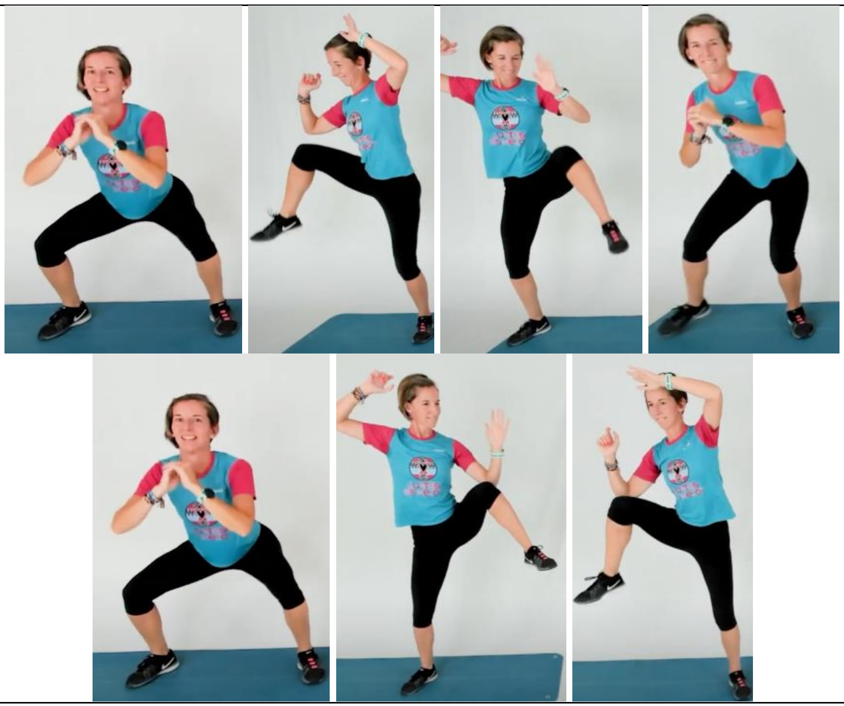 |
| | **Level 2** |
| | 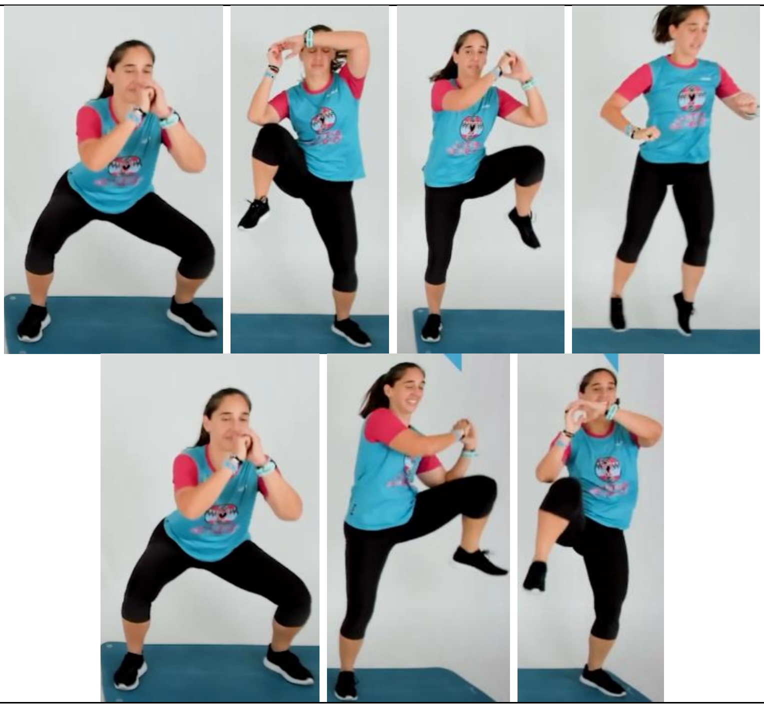 |

| | |
|---|---|
| Lateral lunge step touching opposite toe + knee elevation to opposite hand | **Level 1** |
| | 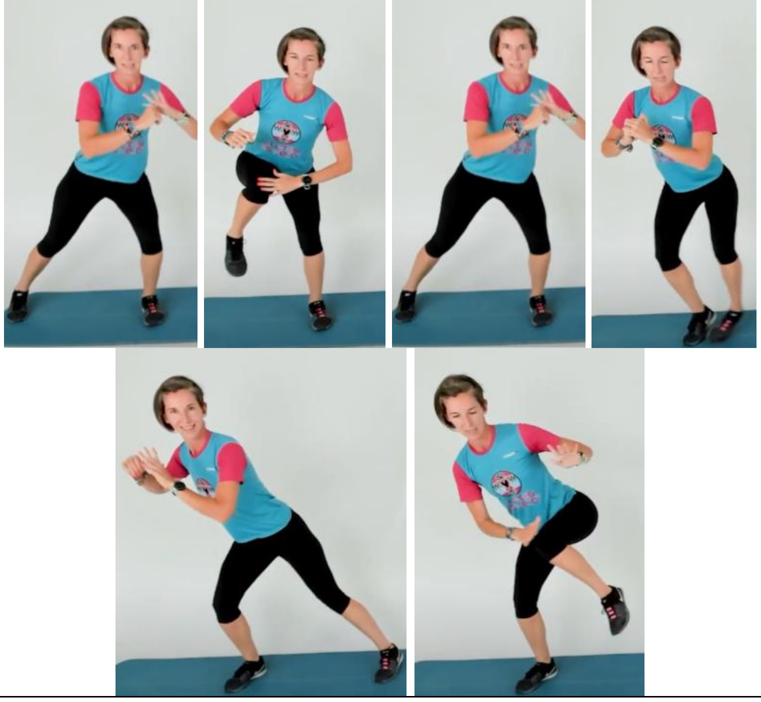 |
| | **Level 2** |
| | 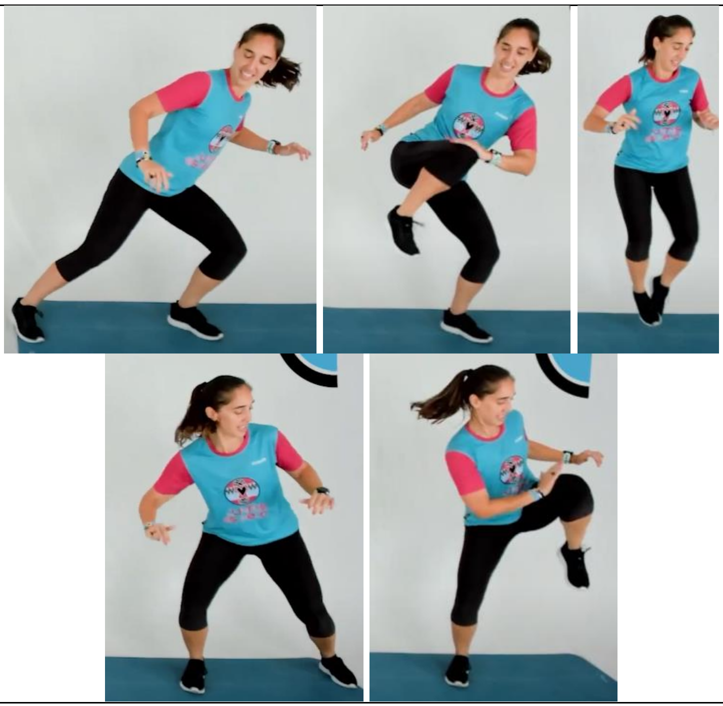 |
| Supine position: arms opening and closing + extension | **Level 1** |
| | 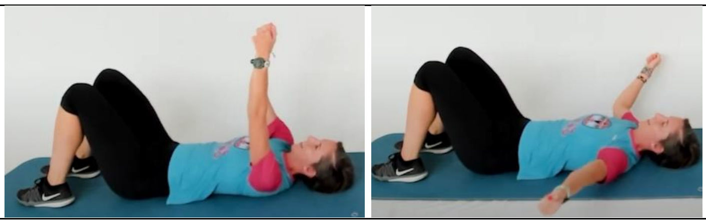 |

| | |
|---|---|
| | 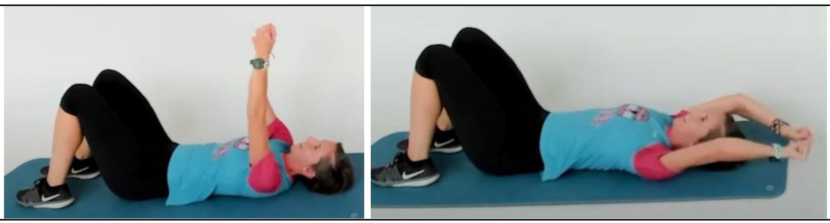 |
| | **Level 2** |
| | 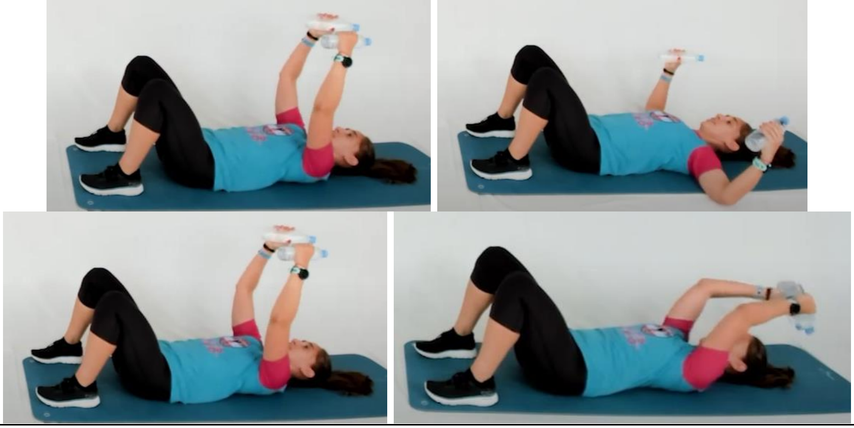 |
| Lateral plank + one leg touch front and back and toe touch with hand | **Level 1** |
| | 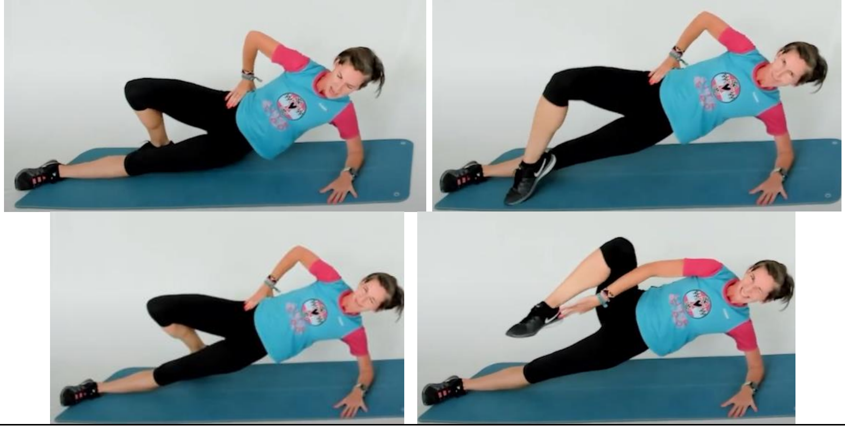 |
| | **Level 2** |
| | 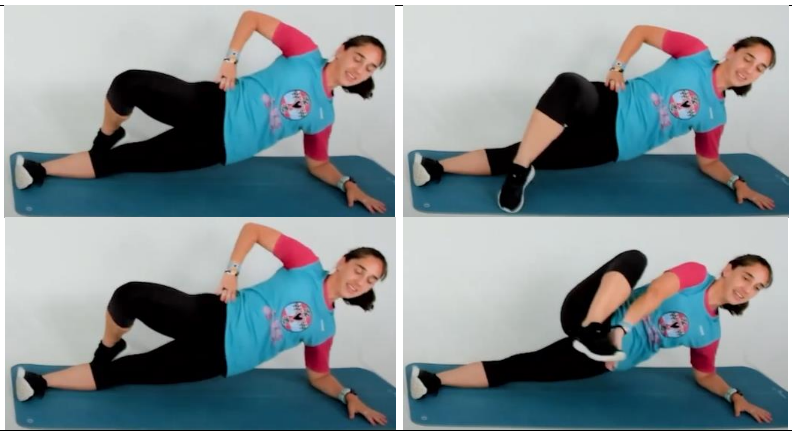 |

## *Week 4 - Session 1 (Link)*

| Exercise | Photo |
|---|---|
| Lateral movement + knee crossing | **Level 1** |
| | 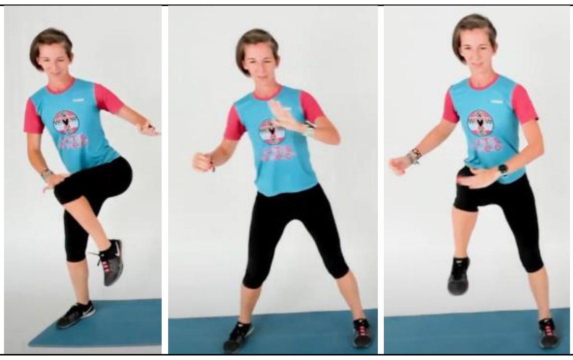 |
| | **Level 2** |
| | 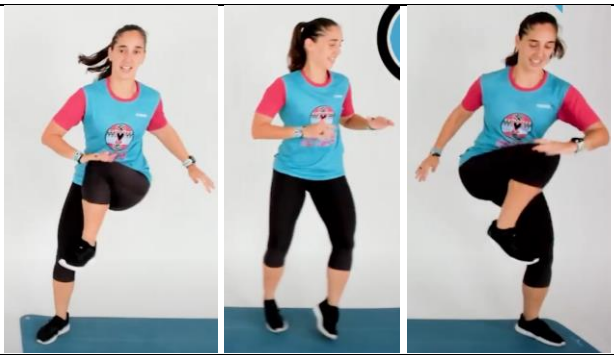 |
| Squat + kick | **Level 1** |
| | 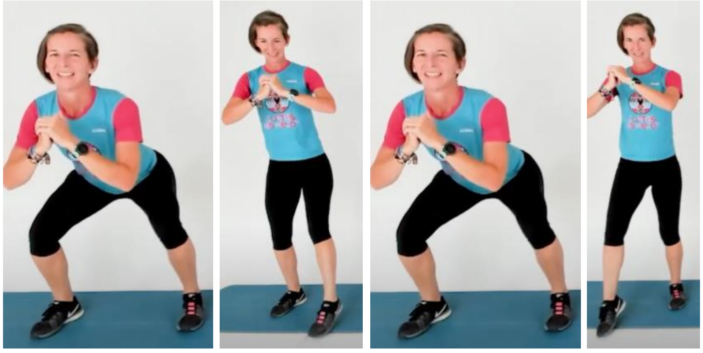 |
| | **Level 2** |
| | 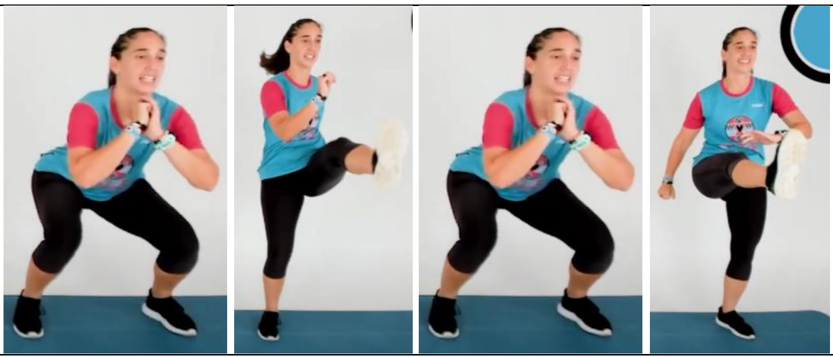 |

| | |
|---|---|
| | **Level 1** |
| Arm extension up and column inclination | 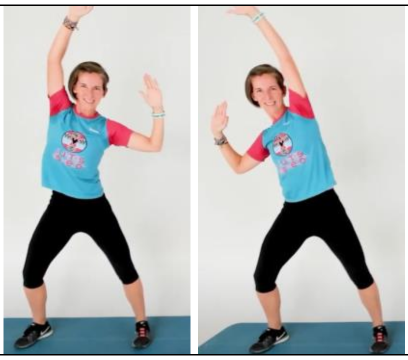 |
| | **Level 2** |
| | 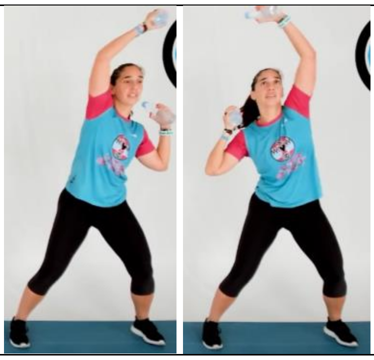 |
| | **Level 1** |
| Sit down and hip elevation | 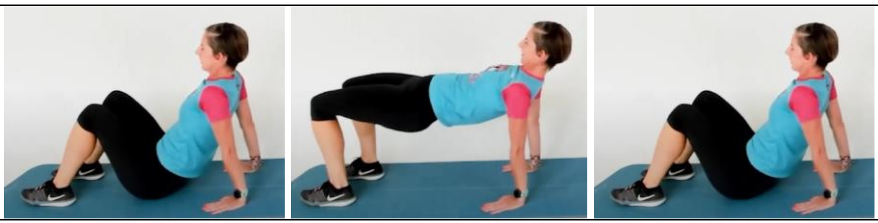 |
| | **Level 2** |
| | 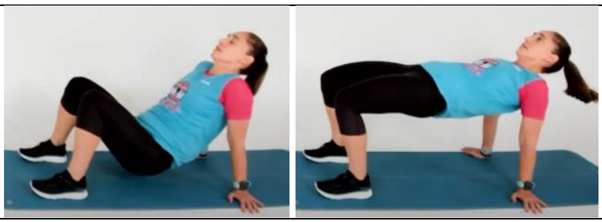 |

## *Week 4 - Session 2 (Link)*

| Exercise | Photo |
|---|---|
| | **Level 1** |
| Lateral movement + knee crossing and arms extension | 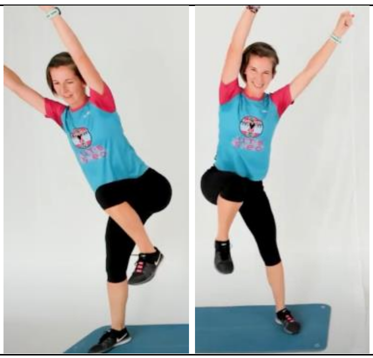 |

<table>
<tr><td></td><td>Level 2<br>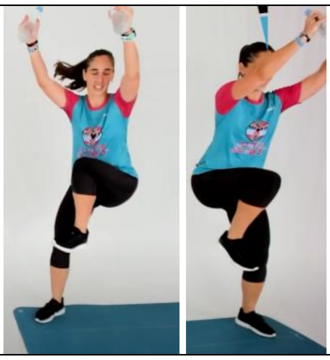</td></tr>
<tr><td rowspan="2">Squat + kick + sprint</td><td>Level 1<br>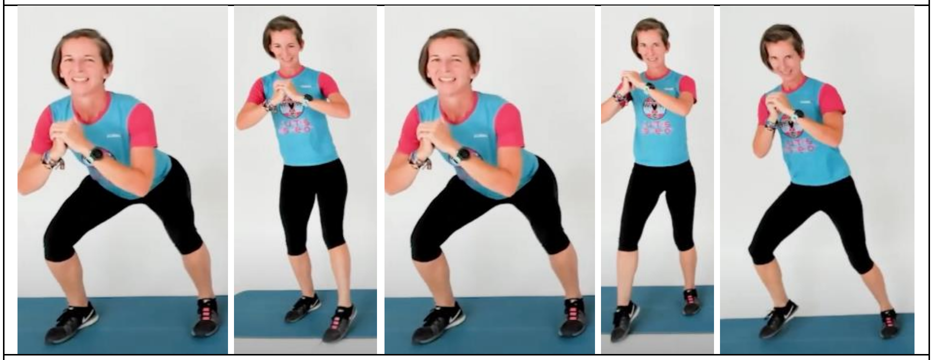</td></tr>
<tr><td>Level 2<br>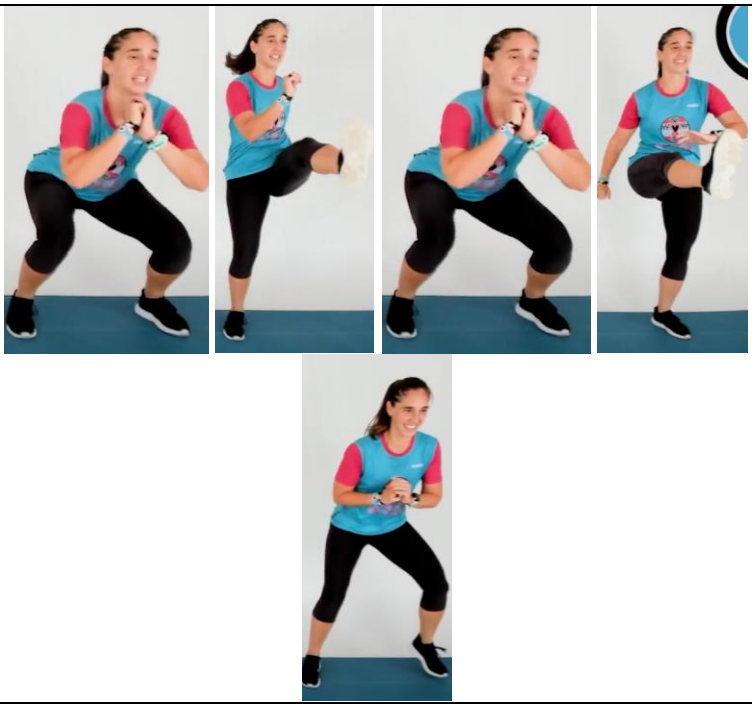</td></tr>
</table>

<table>
<tr><td rowspan="4">Arm extension up and column inclination + circle with the arms</td><td>Level 1</td></tr>
<tr><td>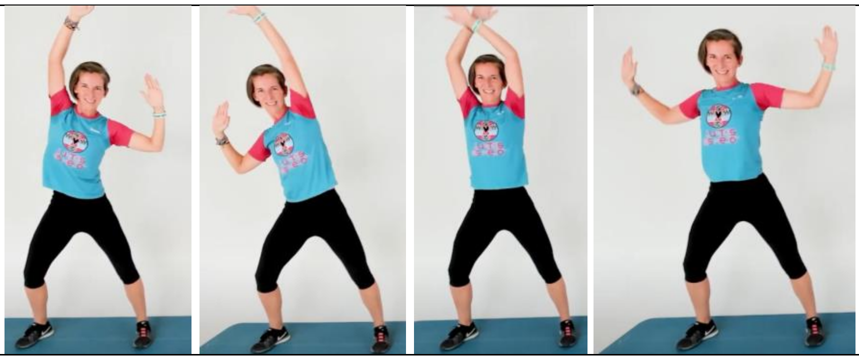</td></tr>
<tr><td>Level 2</td></tr>
<tr><td>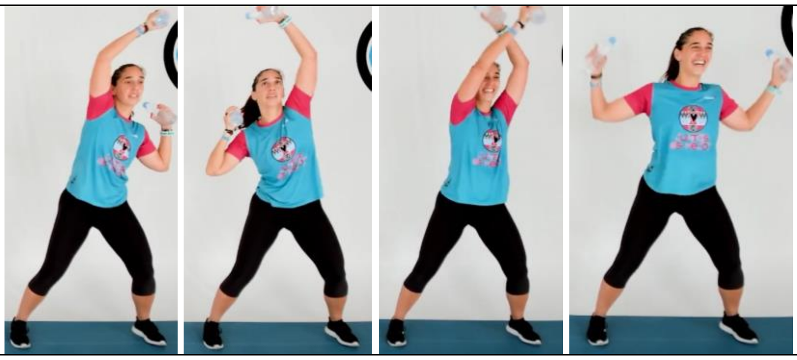</td></tr>
<tr><td rowspan="4">Sit down and hip elevation + one leg extension</td><td>Level 1</td></tr>
<tr><td>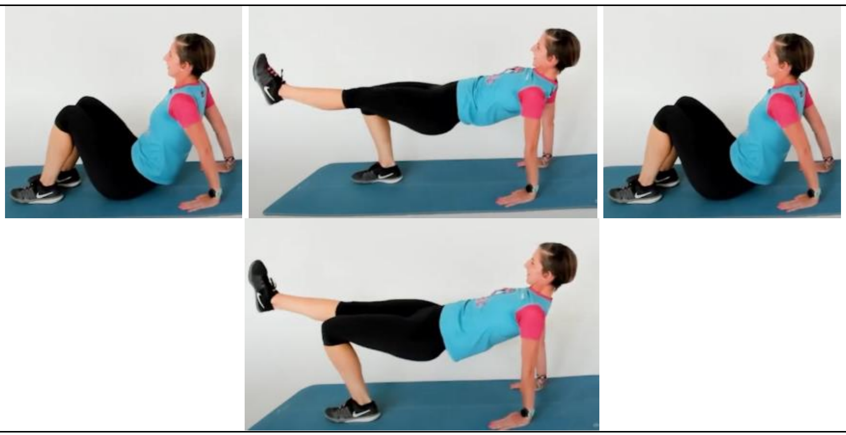</td></tr>
<tr><td>Level 2</td></tr>
<tr><td>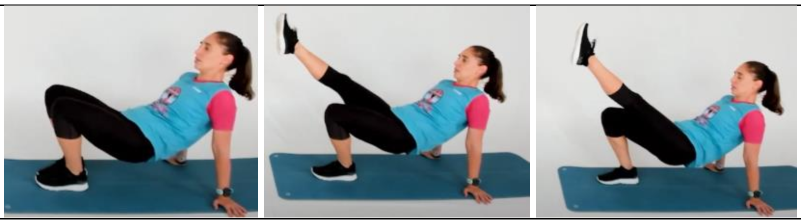</td></tr>
</table>

## *Week 5 - Session 1 (Link)*

| Exercise | Photo |
|---|---|
| Punch + squat + twist | **Level 1** |
| | 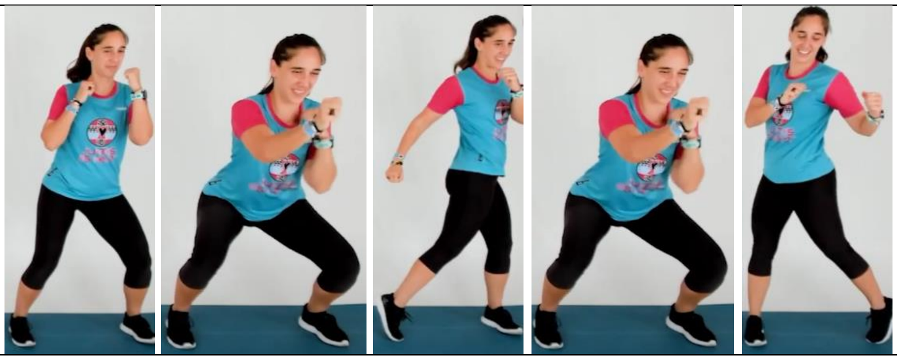 |
| | **Level 2** |
| | 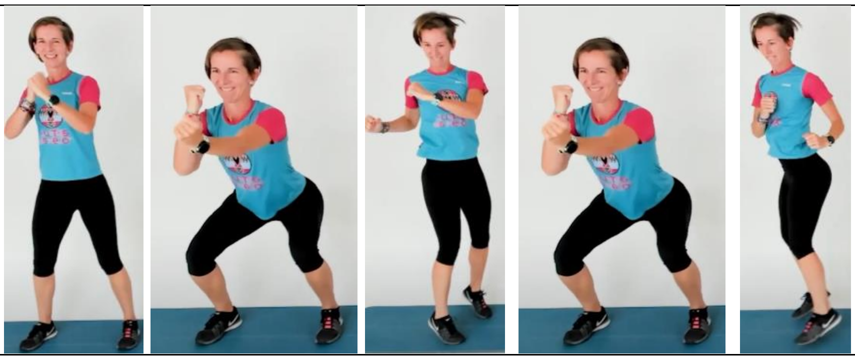 |
| Lateral kick | **Level 1** |
| | 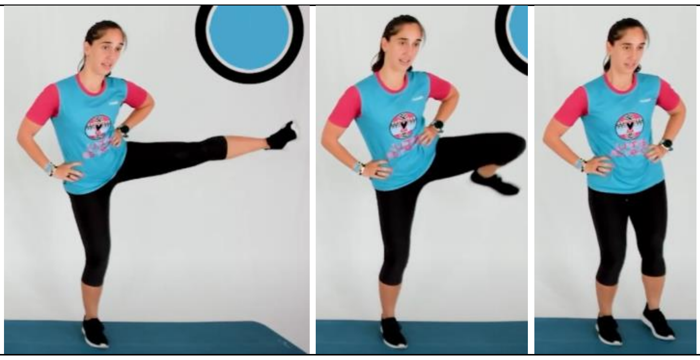 |
| | **Level 2** |
| | 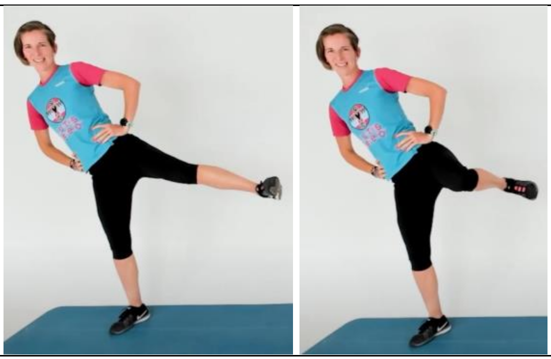 |

| | |
|---|---|
| Front step hands to plank + mountain climbing or front jump + back step with hands | **Level 1**<br>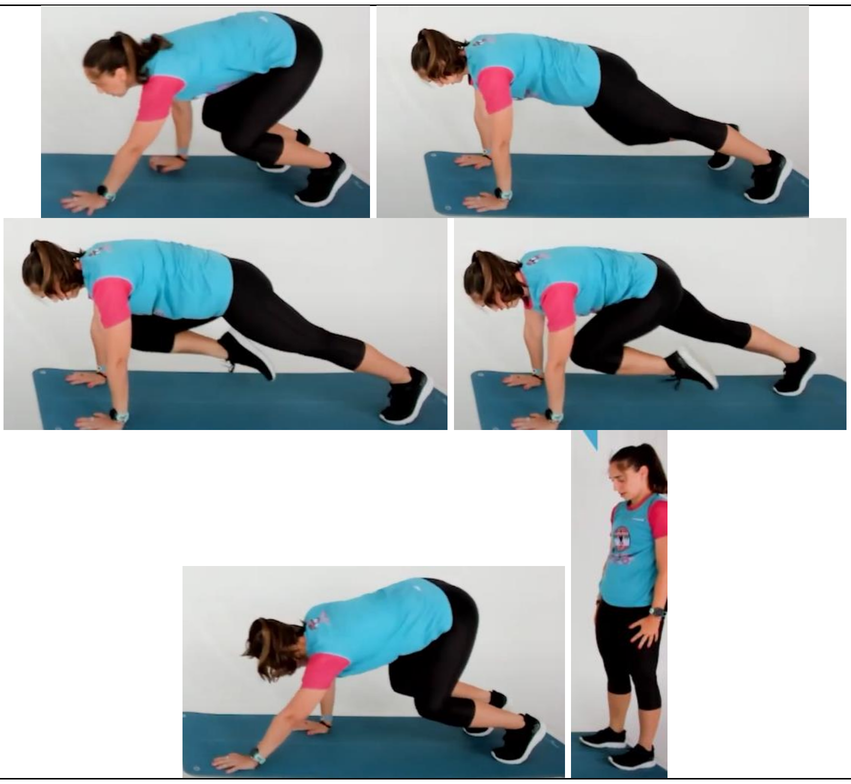<br>**Level 2**<br>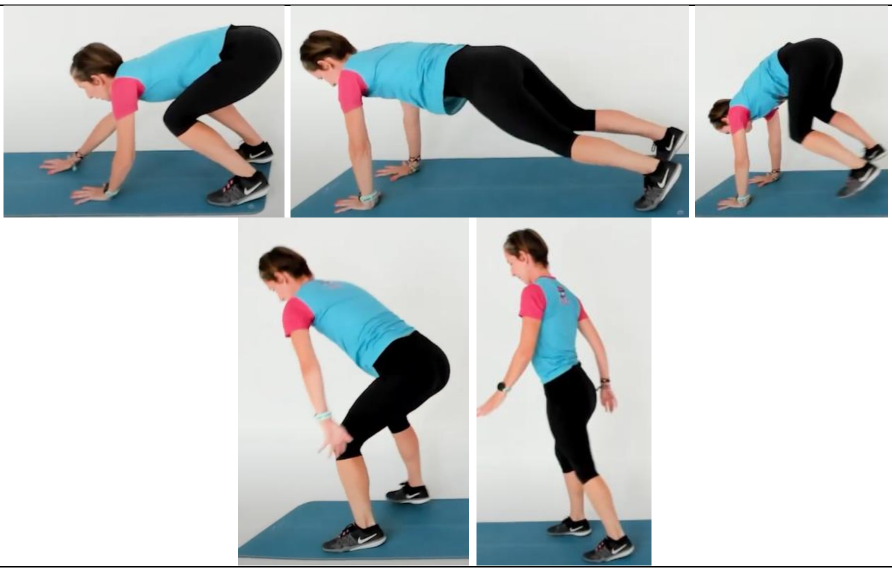 |
| Balasana to plank kneeling or not kneeling | **Level 1**<br>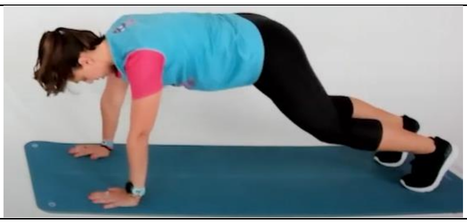 |

| | |
|---|---|
| | 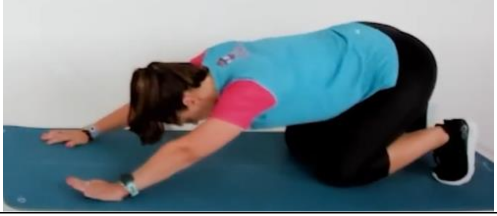 |
| | **Level 2** |
| | 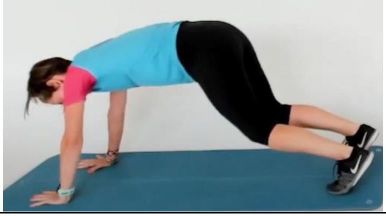 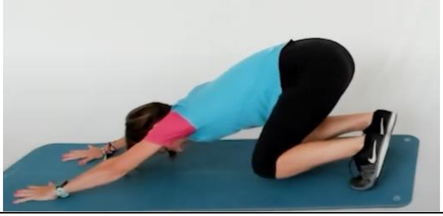 |

## *Week 5 - Session 2 (Link)*

| **Exercise** | **Photo** |
|---|---|
| Punch + squat + twist x4 | **Level 1** |
| | 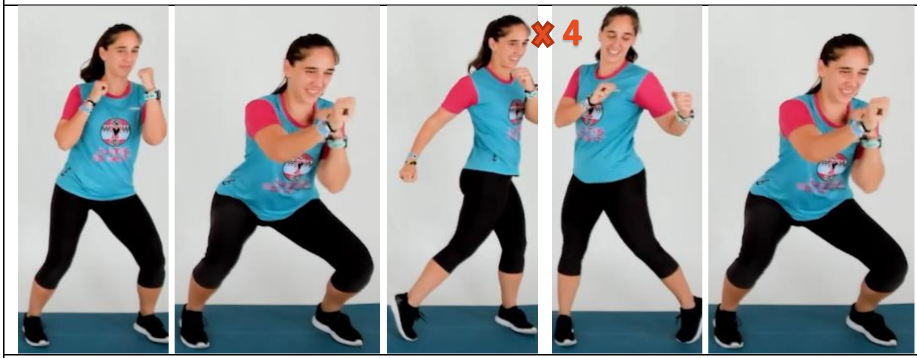  |
| | **Level 2** |
| | 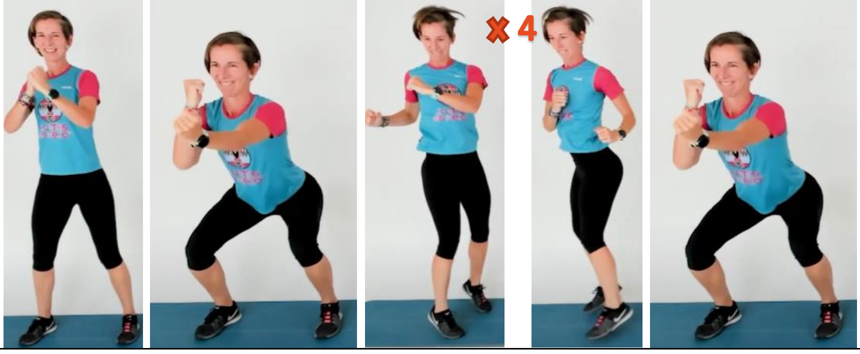  |
| Lateral kick + arm extension up | **Level 1** |
| | 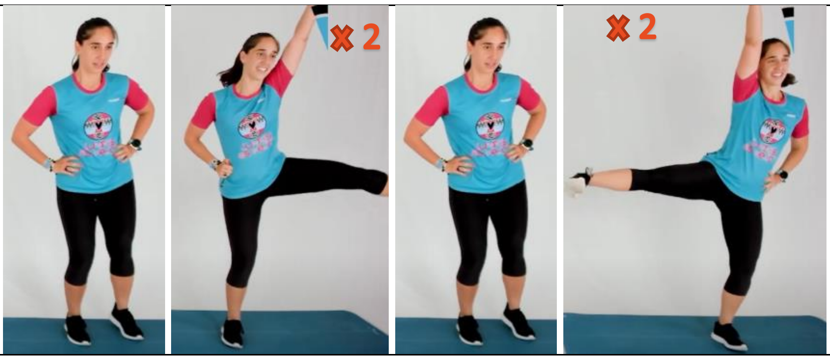  |
| | **Level 2** |

<table>
<tr><td></td><td>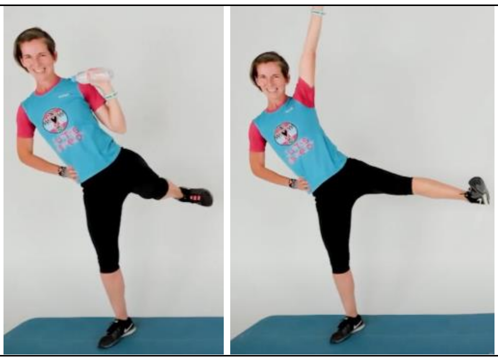</td></tr>
<tr><td rowspan="4">Front step hands to plank + mountain climbing or front jump + touch opposite shoulder + back step hands</td><td>Level 1</td></tr>
<tr><td>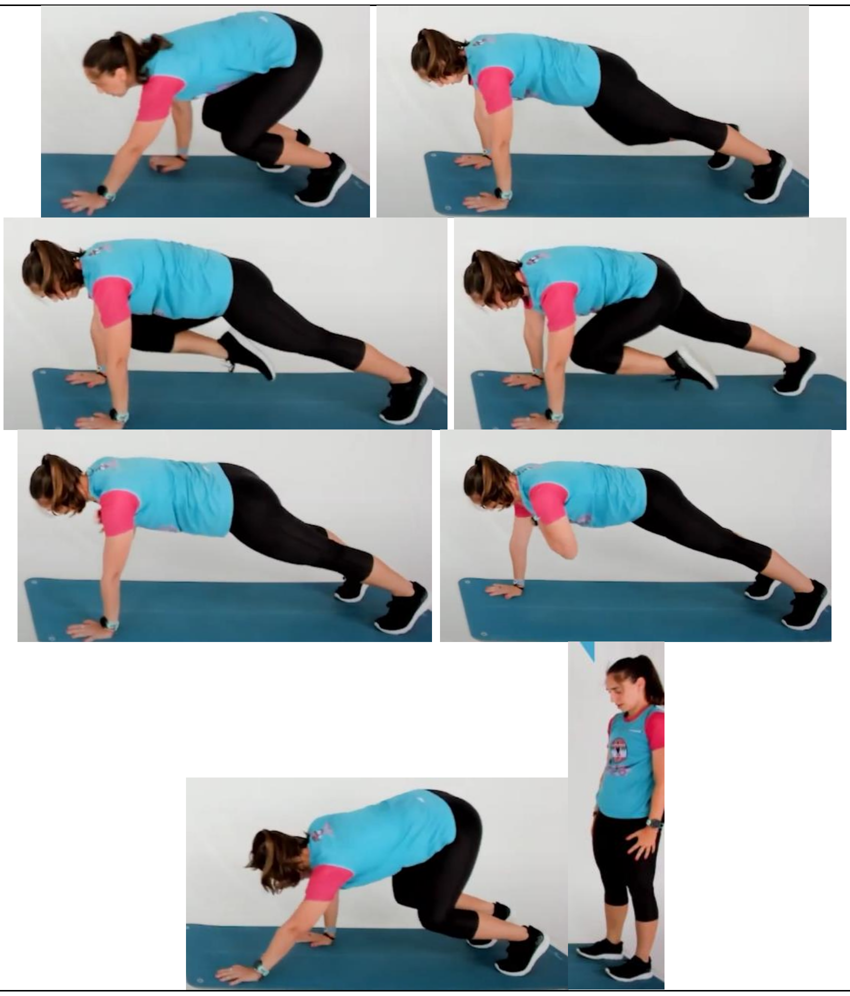</td></tr>
<tr><td>Level 2</td></tr>
<tr><td>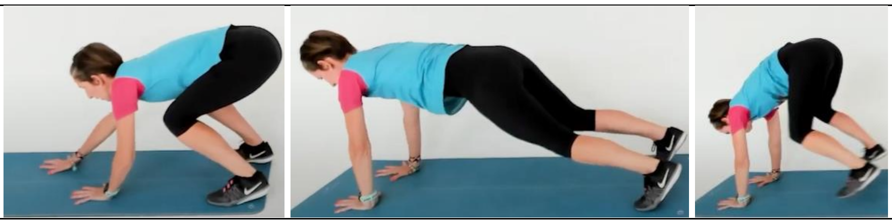</td></tr>
</table>

<table>
<tr><td></td><td>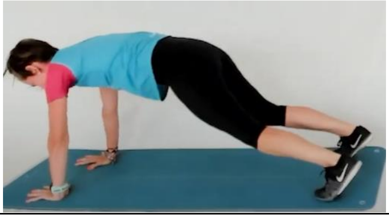 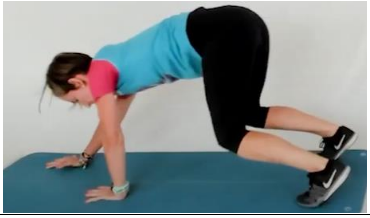 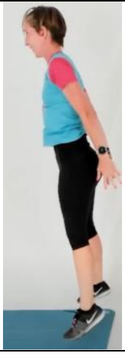</td></tr>
<tr><td rowspan="4">Balasana to plank kneeling or not kneeling + mountain climber</td><td>Level 1</td></tr>
<tr><td>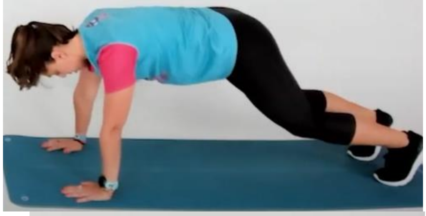 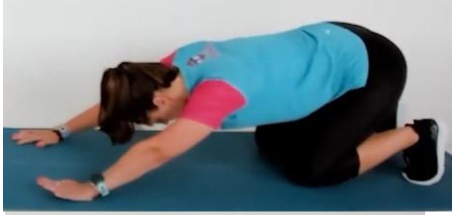<br>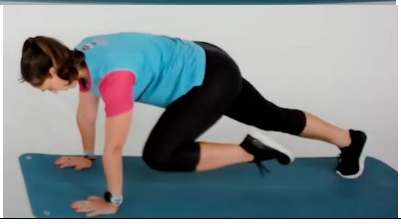 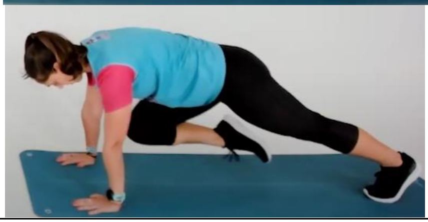</td></tr>
<tr><td>Level 2</td></tr>
<tr><td>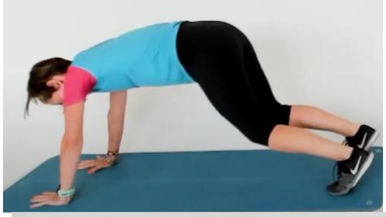 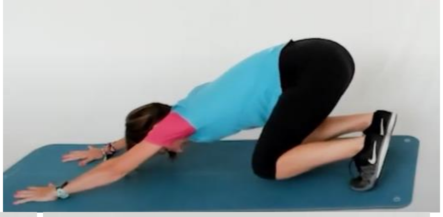<br>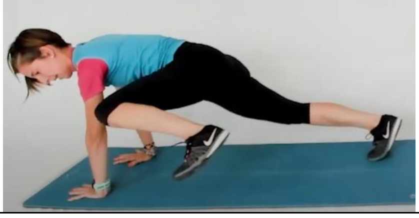 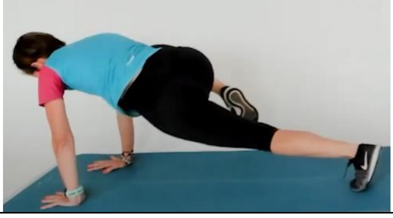</td></tr>
</table>
