# Supplementary material for: Women’s Involvement in Steady Exercise (WISE): Study Protocol for a Randomized Controlled Trial
Source: Healthcare (Basel). 2023 Apr 29;11(9):1279. doi: 10.3390/healthcare11091279 (PMC10177760; doi:10.3390/healthcare11091279)
Supplement: Supplementary file 1 [file healthcare-11-01279-s001.zip › Table S2_Table S2 Messages sent to the participants.pdf]

Table S2: Messages sent to the participants as readings about education in healthy habits.

Pop-up message sent at the beginning of the WISE program: ‘Hi WISE girls! Welcome to WISE program, and CONGRATULATIONS for being selected to be part of this group. We are absolutely sure that being WISE, you will learn, enjoy an experience positive changes in your body, mind and health. We will accompany you on this path. Close your eyes, take a breath and…LET’S GO!’
Pop-up message sent at the end of 1st week: ‘Hi girls! We've already done a week of HIIT workouts. We want you to know more about the type of exercise you are doing. Click on the following link and discover what HIIT is (*1st reading)’
Pop-up message sent before 1st session of each week, during the first two months: ‘Hi girls! remember that this is a HIIT session, and as such, you have to do the 20 seconds of activity to the FULLEST! 💪 🏃‍♀️ 🏃‍♀️🏃‍♀️💪🔝 🔝’
Pop-up message sent at the end of 2nd week: ‘Did you know that it takes 21 days for noticeable changes to occur in any habit you start? So, we ask you to be constant, and to hold out until at least the 21st, so you can decide if you have noticed any benefits since you started our program. Follow the link to know what are the main changes you will notice after 21 days of training. We assure you that they are worth it! (**2nd reading)’
Pop-up message sent at the beginning of 3rd week: ‘Hi girls! We start the third week of the program. If some week it is not possible for you to do the two sessions, you can recover the missed session at another time: but as long as you leave a rest day between two sessions, and that you do not do more than 3 sessions a week.
Pop-up message sent at the end of 2nd month: ‘Hi WISE girls! You have already completed the first two months of the program! CONGRATULATIONS!! Next week we will start with the next phase, in which the rest time is reduced to 10 seconds. If you have done all the sessions of the program, you can take a rest session, skipping one of the sessions this week, but replacing it with a 30-minute walk at a vigorous and constant pace.
Pop-up message sent at the beggining of 3th month: Hello girls! You have been active for 2 months now. CONGRATULATIONS!! But there is still something else you can do to make the program even more effective: dedicate a third day of the week to doing some kind of aerobic exercise: easy running, cycling, rowing or swimming are activities that perfectly complement our HIIT sessions. The WHO recommends doing 3 days of exercise a week: if you add one day of activity to our program you will already be achieving it! 🏃🚴🏊🚣👏 👏🔝 🔝’
Pop-up message sent at the end of 3rd month: ‘Hello girls! You have already completed 3 months of the program! CONGRATULATIONS!! The scientific literature considers that 3 months is the time that any exercise program has to last for significant changes to occur in your body. Do you notice any change? For sure yes! And if not, be constant, they will arrive very soon! If you have gone more than 10 sessions without missing a session, you can take a rest session as long as you replace it with a 30-minute walk at a vigorous and constant pace, or a swimming or cycling morning/afternoon.’
Pop-up message sent at the mid of 4th month: ‘Hello girls! Christmas holidays are coming soon. And possibly after them, some of you will be in exam periods. Remember that excess food or sitting for many hours to study can be compensated for with physical exercise: if you do our sessions you will feel much better both physically and mentally. So be constant! And remember that if some week it is not possible for you to do the two sessions, you can recover the missed session at another time: but as long as you leave a rest day between two sessions, and that you do not do more than 3 sessions a week. Also, if you have gone more than 10 sessions without missing a session, you can take a rest session as long as you replace it with a 30-minute walk at a vigorous and constant pace, or a swimming or cycling session.

(* 1st reading)

Do you know what HIIT is?

HIIT or High Intensity Interval Training is based on performing short but intense exercise sessions followed by intervals of active rests of lower intensity that guarantee recovery.

…………………

What is HIIT for?

Repeating these sessions promotes the achievement of healthy goals such as strengthening muscles, increasing energy expenditure with the consequent loss of fatty tissue, and improving heart and lung health. In addition, HIIT can be personalized, thereby guaranteeing access to these exercises for people who do not yet have a consolidated exercise routine.

…………………

How do I start?

If you have never practiced HIIT, it is recommended that you start with one or two weekly sessions lasting no more than 10-12 minutes/session, avoiding exercises that involve impact, such as jumping jacks. Remember to incorporate the recovery period to catch your breath before continuing with the next exercise. As you progress, the sessions can be extended over time both in frequency and intensity as well as in duration.

…………………

Tabata

Tabata is a very practical type of HIIT training for beginners. Dare to carry out this type of exercise that we propose and that we will send you through videos starting next week, and that consists of two simple steps:

1) 20 seconds of high intensity exercise, doing as many reps as you can
2) 10 seconds rest

Repeat this cycle for a total of 8 rounds and you will only have invested 4 minutes of your time!

Some exercises that you will do in our HIIT sessions are:

Cardio: jumping jacks, energetic dance, mountaineers, run on site, running high knees

Force: squats, lunges, push-ups, planks, good Morning

Remember: 20 seconds of HIIT and 10 seconds of rest. For success, repeat 4 times doing your best.

…………………

When is it advisable to do HIIT?

Since HIIT is a high-intensity workout, it is recommended to do it at any time of the day when you feel full of energy, but after a reasonable period after the last meal. Similarly, avoid doing HIIT before going to bed to ensure a good rest.

…………………

What will I need?

Comfortable clothes, sports shoes, and ¡the wish to disconnect from your worries and let yourself go!

Patri and Paula will help you with the rest. They will explain to you the postural corrections that will prevent you from getting injured (such as keeping your back straight at all times, your shoulders back, your knees aligned, and your abdomen and buttocks contracted).
You can meet friends to make the session more enjoyable, and to help you at the beginning, which is when it is most likely to be difficult to start.
And remember to hydrate well, before and after the session.
All sessions will start with a short warm-up and end with some gentle stretching. That will be repeated for a period of 8 sessions so that you can learn how to do them and that way you can incorporate them on the days that you decide to do some exercise on your own.
…………………

Will I feel stiff?

After sports practice when there is no routine, it is normal feeling stiff. However, the appearance of these is a good indicator that you are pushing yourself to the maximum, as HIIT training requires.
To avoid the discomfort that these present, try to introduce yourself to HIIT progressively and spacing training throughout the week. But it is very important that you do not stop training because if you do you will suffer them again when you return to exercise.

NOW YOU KNOW EVERYTHING YOU NEEDED TO BE ABLE TO START OUR SESSIONS. GET READY, THIS STARTS IN 3, 2,...!

(**2nd reading)

Did you know that it takes 21 days for noticeable changes to occur in any habit you start? So, we ask you to be constant, and to hold out until at least the 21st, so you can decide if you have noticed any benefits since you started our program.

These are the main changes you will notice after 21 days of training. We assure you that they are worth it!

1. Greater relaxation. During sports practice your body releases endorphins that will influence your physical and mental state.
2. Balanced energy expenditure. Carrying out physical activities will help to activate the catabolism of energy reserves, which will cause a mobilization and expenditure of body fat.
3. Cleaner and brighter skin. Sport stimulates blood circulation, which favors the removal of dirt from the skin.
4. Toned muscles. Sports practice has a direct impact on maintaining muscle tone and strengthening.
5. Less insomnia. Performing an energy expenditure favors falling asleep and ensures a good rest.
6. Lung capacity. Playing sports improves the oxygen transport capacity of the blood and also the absorption of oxygen when the air enters the lungs.
7. Healthy life. Along with diet, regular physical activity is essential to maintain optimal health and minimize the risk of cardiovascular disease.
8. Mood. Sport influences your mood in a positive way by releasing stress from day to day. You'll be in a better mood.
9. Mental performance. Sports practice has been positively correlated with long-term cognitive improvement.
10. Greater confidence. Your confidence and enthusiasm will be increased as you notice how you are achieving the objectives set in the short and medium term when it comes to adherence in sports practice.

Nutritional tip 1: We are composed of 60 percent water, and this figure alone should be enough to understand how important this element is to our bodies. Water covers countless functions including transporting nutrients, regulating body temperature, aiding digestion, aiding the removal of toxins and metabolic waste, and so much more.

Therefore, we can understand how important it is to drink and thus keep the body hydrated constantly.<br>But how much should we drink?<br>Three percent of our body weight or similarly, that is 30 milliliters of water for every kilogram of weight.<br>Let's make an example: a 50 kg girl will need to drink at least 1.5 liters of water per day; the amount will be even higher in case of physical activity.<br>In the latter case, depending on the outside temperature, the activity practiced and other factors, we can consider introducing 500 ml to 1 liter of water for every hour of physical activity performed.<br>And in the summertime, considering very high outside temperature, we can increase the amount of water even more.<br>Remember that we can go weeks without eating, but only a few days without drinking!
**Nutritional tip 2:** Start the day with a meal that will supply you with energy and help you stay focused, concentrated and in a good mood throughout the day. Adjusting calorie intake is a sure way to correct your weight, but at the same time it can affect your energy level.
**Nutritional tip 3:** The body needs a certain amount of calories for optimal functioning, and too little or too much intake can make you feel tired. Since food is the fuel that the body uses to produce energy, even when we are not moving, we need to consume a certain amount of food, or calories. How many calories it is depends on numerous factors such as body weight, age, gender, thermoregulation, overall health, etc. Foods that contain complex carbohydrates, as well as fiber, will help you maintain an optimal level of energy throughout the day. These include bananas, fatty fish, wholemeal rice, legumes, apples, quinoa and many others.
**Nutritional tip 4:** In addition to food, energy levels are affected by physical activity, hydration and sleep. Physical activity is associated with improved energy and reduced fatigue, even in the case of a sedentary lifestyle. Also, proper hydration during the day can provide you with energy and help fight fatigue. Insufficient fluid intake, especially water, can cause dehydration. Mild to moderate dehydration can lead to difficulty concentrating, headaches, irritability, lethargy and drowsiness. In addition, the body needs sleep to restore and preserve energy. Its deficiency leads to a drop in energy, focus and concentration, and can worsen overall health.
**Nutritional tip 5:** Why is it important to consume seasonal and local food? Regardless of the fact that food has become much more available globally nowadays, without any obstacles in delivering from one end of the world to the other, we advise you to choose seasonal and local foods whenever you have the opportunity because they contain all the nutrients the body needs during the season in progress. So, the food we eat in the period of the year in which it succeeds is the best choice for preserving the health of the whole organism.
**Nutritional tip 6:** It is a great misconception that healthy food is tasteless! Experiment with cooking. To avoid the feeling that you always eat the same dishes, find new recipes and combine foods in different ways. Come up with a recipe yourself, and share it with your WISE group. In this way you will encourage other women that are in the same process as you.
**Nutritional tip 7:** Read food labels. Try to avoid: trans fats (partially hydrogenated vegetable oil), monosodium glutamate, high-fructose corn syrup, sodium benzoate E211 and potassium benzoate E212, artificial sweeteners and artificial colors.
**Nutritional tip 8:** Drinking enough water each day is crucial for many reasons: to regulate body temperature, keep joints lubricated, prevent infections, deliver nutrients to cells, and keep organs functioning properly. Being well-hydrated also improves sleep quality, cognition, and mood. The amount of water you have to drink depends on your body weight. Around 3% of your body weight of water is a correct indication. The amount will increase during training

days. It is better to drink natural water and the recommended quantities can also drank with infusions or herbal teas. The best is to drink water far away from meals.
**Nutritional tip 9:** Simply waiting for the sensation of thirst is not a good enough sign of a need to drink. By the time we feel thirsty our body is already dehydrated and potentially suffering some of the effects. Equally, simply drinking to eliminate the feeling of thirst doesn't fully hydrate the body. The best indicator of good hydration is urine color, a pale straw colored urine being a reliable indicator of good hydration. Darker coloured urine is a sure sign that the body needs more fluid. Getting into the habit of drinking regularly is a great way of keeping hydrated.
**Sleeping tip 1:** Everybody needs a different amount of sleep. Studies show that most adults feel their best after getting seven hours of sleep. Children, teenagers and young adults need more sleep. So, age, genetics, environment and medical conditions all play a part in how much sleep our body needs.
**Sleeping tip 2:** If you feel relaxed and energised, you've had a good night's rest. If you haven't slept enough, you might feel tired.<br>Don't take off your smart band at night and keep track of your sleep duration.
**Wellbeing tip 1:** Measuring your breathing can give you an understanding of your body's wellbeing. It is essential to measure a person's respiratory rate at rest to determine whether it is normal. Exercise or even walking across a room can affect it. Respiratory rates change based on many health and activity factors. Normal respiratory rates also differ in adults and children. The respiratory rate is the number of breaths someone takes every minute. It is one of the main vital signs, along with blood pressure, pulse and temperature.
**Wellbeing tip 2:** When a person inhales, oxygen enters their lungs and travels to the organs. When they exhale, carbon dioxide leaves the body. A normal respiratory rate plays a critical role in keeping the balance of oxygen and carbon dioxide even in the body. The normal respiratory rate for healthy adults is between 12–20 breaths per minute. At this breathing rate, the carbon dioxide exits the lungs at the same rate that the body produces it. Breathing rates of below 12 or above 20 can mean a disruption in the normal breathing processes.
**Wellbeing tip 3:** Your heart rate is the number of times your heart beats per minute. Throughout the day your heart speeds up and slows down to accommodate your body's changing need for oxygen. Your heart rate is lower when you are at rest. When you exercise, you heart speeds up and pumps more blood, which allows oxygen-rich blood to flow easily and reach your muscles. To measure your heart rate, simply check your pulse.
**Wellbeing tip 4:** When your heart pumps blood through your body, it creates a pulse that you can feel on the arteries that are close to the skin's surface. Although you may be able to feel your blood pumping in a number of places—your neck, the inside of your elbow, and even the top of your foot—your wrist is probably the most convenient and reliable place to get a good pulse.
**Wellbeing tip 5:** To check your pulse at your wrist, place the tips of your index and middle finger on the palm side of your other wrist, below the fat pad of your thumb. Press lightly with your fingers until you feel the blood pulsing beneath your fingers. You may need to move your fingers around until you feel the pulsing. When you feel your pulse, count the number of beats in 15 seconds. Multiply this number by four to calculate the beats per minute to get your heart rate. See how your body responds in different situations by measuring your heart rate. You can do it in the morning to track your heart rate at rest, and do it after exercising or drinking coffee.
